# Market Competitiveness Evaluation of Mechanical Equipment with a Pairwise Comparisons Hierarchical Model

**DOI:** 10.1371/journal.pone.0146862

**Published:** 2016-01-19

**Authors:** Fujun Hou

**Affiliations:** School of Management and Economics, Beijing Institute of Technology, Beijing 100081, P.R. China; Southwest University, CHINA

## Abstract

This paper provides a description of how market competitiveness evaluations concerning mechanical equipment can be made in the context of multi-criteria decision environments. It is assumed that, when we are evaluating the market competitiveness, there are limited number of candidates with some required qualifications, and the alternatives will be pairwise compared on a ratio scale. The qualifications are depicted as criteria in hierarchical structure. A hierarchical decision model called PCbHDM was used in this study based on an analysis of its desirable traits. Illustration and comparison shows that the PCbHDM provides a convenient and effective tool for evaluating the market competitiveness of mechanical equipment. The researchers and practitioners might use findings of this paper in application of PCbHDM.

## 1 Introduction

The increasing complexity of the socio-economic environment makes it an important yet complicated task to evaluate the market competitiveness concerning mechanical equipment. The competitiveness evaluation of mechanical equipment includes many different objectives and reflects the wishes of wide-ranging interests, for example, in addition to the quality, the mechanical performance and the price, such influential factors as occupant comfort, ease of training and maintenance, as well as the environmental considerations have to be taken into account. The evaluation task comprises, therefore, a multi-criteria decision making problem involving some complicated even conflicting objectives or attributes.

During the past 35 years, the Analytic Hierarchy Process (AHP) developed by Saaty [1] has often been utilized to evaluate alternatives in multi-criteria decision environments (e.g., [2]). However, there have been a number of criticisms on Saaty’s AHP among which the rank reversal has attracted everlasting controversies among researchers. The rank reversal refers to a phenomenon that the ranking of alternatives produced by Saaty’s AHP may be altered by the addition or deletion of another independent alternative for consideration [3]. Dyer thus pointed out that the rankings produced by Saaty’s AHP is arbitrary [4]. Triantaphyllou also reported that the decision maker may never know the exact ranking of the alternatives in a given multi-criteria decision-making (MCDM, for short) problem when the original AHP, or its current additive variants, are used [5].

Considering these possible shortcomings, this paper would not use Saaty’s AHP, instead, we used another hierarchical decision-making technique, specifically the pairwise comparisons based hierarchical decision model (PCbHDM, for short) [6, 7], to evaluate the market competitiveness of mechanical equipment in a multi-criteria decision-making environment. The reason for our choice will be interpreted in more detail in Section 3.

The remainder of the paper is organized as follows. Section 2 provides a description of the problem. Section 3 describes how the decision-making method was selected. Section 4 includes illustration, comparison and discussion, and Section 5 contains our concluding remarks.

## 2 Description of the problem

Evaluation of market competitiveness of industrial products is fairly important in today’s highly competitive environment. Both the sellers and the buyers could benefit from the assessment based on which the sellers may improve their products and services for gaining the competitive advantage, and the buyers may purchase the product with highest degree of desirability. A careful market competitiveness evaluation system should cover all the criteria that buyers and sellers are interested in. In the development of such an evaluation system, the basic problem is to score and to rank a set of alternatives given some decision criteria. Therefore, the identification of criteria and integration of experts’ assessments are two components of the evaluation system.

Let {*A*_1_,*A*_2_,…,*A*_*m*_} be the set of alternatives and {*c*_1_,*c*_2_,…,*c*_*n*_} be the set of criteria according to which the desirability of an alternative is to be compared. The purpose of the evaluation process is a quantitative comparison among the available alternatives in the context of a multi-criteria decision environment. In the mechanical equipment evaluation system, the alternatives refer to the machines having same functions but coming from different producers. The criteria stand for the concerns both of the sellers and the buyers.

## 3 Selection of decision-making method

### 3.1 Pairwise comparison matrices

Denote by *X* = {*x*_1_,*x*_2_,…,*x*_*n*_} the object (alternative or criterion) set. A pairwise comparison matrix (PCM, for short) over the objective set is an useful tool for deriving object weights or priorities, where the component can be assumed of different meanings as described in the following.

**Multiplicative type:** [1] For a *n* by *n* PCM *M* = (*p*_*ij*_) with ∀*i*,*j*(*p*_*ij*_ ∈ (0,+∞)), the conditions of reciprocity and consistency are given by ∀*i*,*j*(*p*_*ij*_ *p*_*ji*_ = 1) and ∀*i*,*j*,*k*(*p*_*ij*_ *p*_*jk*_ = *p*_*ik*_), respectively. The component *p*_*ij*_ represents the preference ratio of object *x*_*i*_ over *x*_*j*_. For a consistent multiplicative PCM *M* = (*p*_*ij*_), a *n* ×1 vector *W* = (*ω*_*k*_) with *ω*_*k*_ > 0, is called a priority vector of *M* such that ∀*i*,*j* ∈ *I*,*p*_*ij*_ *ω*_*j*_ = *ω*_*i*_.
**Fuzzy type:** [8] For a *n* by *n* PCM *F* = (*f*_*ij*_) with ∀*i*,*j*(*f*_*ij*_ ∈ [0, 1]), the conditions of reciprocity and consistency are given by ∀*i*,*j*(*f*_*ij*_+*f*_*ji*_ = 1) and ∀*i*,*j*,*k*(*f*_*ij*_+*f*_*jk*_ = *f*_*ik*_ − 0.5), respectively. The component *f*_*ij*_ represents the preference degree of object *x*_*i*_ over *x*_*j*_. For a consistent fuzzy PCM *F* = (*f*_*ij*_), a *n* × 1 vector *W* = (*ω*_*k*_) is called a priority vector of *F* such that ∀*i*,*j* ∈ *I*,*f*_*ij*_ = *ω*_*i*_ − *ω*_*j*_+0.5.

A fuzzy PCM is also called a fuzzy preference relations in literatures. Moreover, there exists an isomorphism mapping between the set of fuzzy PCMs and the set of multiplicative PCMs [9–12].

### 3.2 Saaty’s AHP and related issues

Saaty’s AHP is one of the extensively used MCDM techniques. Application of the AHP involves the following steps [1]:

**Step 1:** Structure the decision problem as a hierarchy of elements (goal, criteria, and alternatives).
**Step 2:** Make pairwise comparison of elements at each level of the hierarchy with respect to each criterion on the preceding level and obtain the PCM.
**Step 3:** Compute the consistency ratio of the PCM to determine whether it satisfies a consistency test. If it does not have acceptable consistency, go back to Step 2 and redo the pairwise comparisons.
**Step 4:** Calculate the local priorities of the decision elements by means of the right eigenvector approach.
**Step 5:** Synthesize the local priorities across various levels by using an additive aggregation rule to obtain the final priorities of alternatives.

Saaty presented two modes of his AHP, one is called the original mode where the local priorities are all sum-normalized (by summing to unity), and the other is called the ideal mode where the alternative’s local priorities are max-normalized (by dividing each component by the largest component).

Though widely used, the AHP has also suffered from a number of controversies including, among others,

(1) Rank reversal: A phenomenon that the ranking of alternatives may be altered by the addition or deletion of another independent alternative for consideration [3, 4].
  We consider the Dyer’s example for illustration. There are 4 alternatives (say A,B,C,D) to be evaluated with respect to three criteria (say a,b,c) having equal importance weights. The data is provided as (ratio-scale measurements)
  $$\begin{array}{c} \begin{array}{c} \;\begin{array}{ccc} a & \;b & \;c \end{array}\\ \begin{array}{cc} \begin{array}{c} A\\ B\\ C\\ D \end{array} & \begin{array}{ccc} 1 & \;9 & \;8\\ 9 & \;1 & \;9\\ 1 & \;1 & \;1\\ 8 & \;1 & \;8 \end{array} \end{array} \end{array}. \end{array}$$
  Using the original AHP, when we just evaluate A, B and C, we obtain *A* ≺ *B*; while we evaluate A, B, C and D, the rank is reversed since we obtain *A* ≻ *B*.
  As pointed out in [3] and [13], neither the original AHP nor the ideal AHP has the property of rank preservation. As a result, the ranking produced by Saaty’s AHP is arbitrary [4], and we may never know the exact ranking if we use the original AHP or its current additive variants [5].
(2) The eigenvector approach: Saaty advocated the use of the eigenvector approach for deriving priorities from a given PCM. However, some problems have been observed including: the “Right and Left Eigenvector Inconsistency” which means that the eigenvector method may yield inconsistent results using the right and the left eigenvectors [14], and thus, the outcome may depend on the description of the problem [15, 16]; the derived priority vector can violate a condition of order preservation [17]; the eigenvector method does not have its isomorphic counterpart used for other type of preference information e.g. for the difference information [18].

Here we point out yet another one:

(3) The paradox of the consistency criterion: For a multiplicative PCM, Saaty defined the consistency index by *C*.*I*. = (*λ*_*max*_)/(*n* − 1), where *λ*_*max*_ is the principal eigenvalue of the considered PCM and *n* is its order. For checking acceptable consistency, Saaty proposed a 0.1 threshold for the consistency ratio, namely, *C*.*R*. ≤ 0.1, where *C*.*R*. = *C*.*I*./*R*.*I*. and *R*.*I*. is the average value of the *C*.*I*. obtained from 500 PCMs whose entries were randomly generated using the 1 to 9 scale. As a frequently used criterion of cardinal consistency, however, it may lead to a paradox: if a PCM of *C*.*R*. = 0.1 is acceptable, then, we have no reason to reject a PCM of *C*.*R*. = 0.1 + 10^−10^; and then, a PCM of *C*.*R*. = 0.1 + 2 × 10^−10^ is also acceptable; …; thus, any a PCM is acceptable, and thus we reach a paradox. This paradox resembles the paradox of the heap (also referred to as the ‘Sorites paradox’, sorites being the Greek word for heap). Moreover, Saaty’s criterion has no counterparts for other kinds of PCM.

### 3.3 The PCbHDM and desirable traits

The PCbHDM is another hierarchical decision model different from the Saaty’s AHP. In this subsection, we introduce the steps, the foundations and desirable traits of the PCbHDM [6, 7].

#### 3.3.1 Steps

The following steps are suggested for applying the PCbHDM:

**Step 1:** Break down the decision problem into a hierarchy of decision elements (goal as the top level, criteria and sub-criteria as the middle levels, and alternatives as the terminal level);
**Step 2:** Establish the multiplicative PCMs based on a ratio scale for the decision elements in each level of the hierarchy with respect to one decision element at a time in the immediate upper level.
**Step 3:** Determine whether or not the PCMs have acceptable consistency by
  $$\begin{array}{c} \text{If}\;a_{ik}<a_{jk},\;\text{then},\;\forall l\left(a_{il}\leq a_{jl}\right). \end{array}$$ (1)
  If not, go back to Step 2 and redo the pairwise comparisons.
**Step 4:** Derive the normalized local weight vectors from the PCMs using the row geometric mean method
  $$\begin{array}{c} \omega_{i}={\left(\prod_{j=1}^{n}a_{ij}\right)}^{\frac{1}{n}}. \end{array}$$ (2)
  If a local weight vector is of the criteria(sub-criteria) in a same level with respect to a specific decision element in the immediate upper level, it is then to be sum-normalized; if it is of the alternatives with respect to a terminal criterion, it is then to be min-normalized (by dividing each component by the smallest component).
**Step 5:** Compute the terminal sub-criteria (criteria) weights with respect to the total goal using the Hierarchy Composition Rule
  $$\begin{array}{c} \beta_{j}^{\left(l+1\right)}=\beta_{k}^{\left(l\right)}{\bar{\beta }}_{j}^{\left(l+1\right)}, \end{array}$$ (3)
  where
  - ${\bar{\beta }}_{j}^{\left(l+1\right)}$ denotes the local weight of a sub-criterion in level *l*+1 w.r.t. its immediately preceding criterion/sub-criterion in level *l*(called the father-criterion);
  - $\beta_{j}^{\left(l+1\right)}$ denotes the global weight of the sub-criterion w.r.t. the total goal;
  - $\beta_{k}^{\left(l\right)}$ denotes the father-criterion’s weight w.r.t. the total goal.
**Step 6:** To get an overall priority for each alternative, synthesize the alternative’s local weights using the weighted geometric mean aggregation rule
  $$\begin{array}{c} r_{j}=\prod_{l=1}^{m}{\bar{u}}_{jl}^{\beta_{l}} \end{array}$$ (4)
  where, ${\bar{u}}_{jl}$ is the alternative’s local weight (has been min-normalized in Step 4) w.r.t. the terminal sub-criterion, and, *β*_*l*_ is the terminal sub-criterion’s weight w.r.t. the total goal.

#### 3.3.2 Foundations

Weighted means are frequently used as aggregation rules in MCDM problems. When the weighted mean is used in a MCDM problem, the problem can be decomposed or incorporated, e.g.,

$$\begin{array}{c} 3^{0.2}5^{0.3}9^{0.5}⇌{\left(3\right)}^{0.2}{\left(5^{0.3/0.8}9^{0.5/0.8}\right)}^{0.8}, \end{array}$$

and

$$\begin{array}{c} 0.2\times 3+0.3\times 5+0.5\times 9⇌0.2\times (3)+0.8\times (0.3/0.8\times 5+0.5/0.8\times 9). \end{array}$$

We show this below by two theorems.

**Theorem 3.1** Suppose that the decision matrix of a MCDM problem is $D={\left({\bar{u}}_{ij}\right)}_{n\times m}$ where $\forall i,j({\bar{u}}_{ij}>0)$, and that the criteria weight vector is *B* = (*β*_*l*_)_*m*×1_ where *β*_*l*_ > 0 and $\sum_{l=1}^{m}\beta_{l}=1$. If the weighted geometric mean is used for the aggregation, we have

$$\begin{array}{c} \begin{array}{cc} \prod_{l=1}^{m}{\left({\bar{u}}_{jl}\right)}^{\beta_{l}} & ⇌{\left(\prod_{l=1}^{t}{\left({\bar{u}}_{jl}\right)}^{\frac{\beta_{l}}{\sum_{i=1}^{t}\beta_{i}}}\right)}^{\sum_{i=1}^{t}\beta_{i}}{\left(\prod_{l=t+1}^{m}{\left({\bar{u}}_{jl}\right)}^{\frac{\beta_{l}}{\sum_{i=t+1}^{m}\beta_{i}}}\right)}^{\sum_{i=t+1}^{m}\beta_{i}},\\ & j=1,2,\ldots ,n. \end{array} \end{array}$$

**Theorem 3.2** Suppose that the decision matrix of a MCDM problem is $D={\left({\bar{u}}_{ij}\right)}_{n\times m}$ where $\forall i,j({\bar{u}}_{ij}\geq 0)$, and that the criteria weight vector is *B* = (*β*_*l*_)_*m*×1_ where *β*_*l*_ > 0 and $\sum_{l=1}^{m}\beta_{l}=1$. If the weighted arithmetic mean is used for the aggregation, we have

$$\begin{array}{ll} \sum_{l\;=1}^{m}\beta_{l}\;\left({\bar{u}}_{jl}\right) & \;⇌\left(\sum {}_{i\;=\;1}^{t}\;\beta_{i}\right)\;\sum_{l\;=\;1}^{t}\frac{\beta_{l}}{\sum {}_{i\;=\;1}^{t}}\left({\bar{u}}_{jl}\right)\;+\;\left(\sum {}_{i\;=\;t\;+\;1\;}^{m}\beta_{i}\right)\;\sum_{l\;=\;t\;+\;1}^{m}\frac{\beta_{1}}{\sum {}_{i\;t\;+\;1\;}^{m}\beta_{i}}\;\left({\bar{u}}_{jl}\right),\\ & j\;=\;1,\;2,...,\;n. \end{array}$$

The above two theorems do hold since we just rewrite the weighted means in other forms. However, these two theorems provide a foundation for hierarchical decision models in that they tell WHEN and HOW a MCDM problem can be decomposed or incorporated:

- If the criteria’ weights w.r.t. the total goal are sum-normalized, then, the MCDA problem can be decomposed (the left-to-right means decomposition, i.e., one level to multi-level).
- If the weights of the same level sub-criteria dominated by the same immediate upper level criterion are sum-normalized, then, the decision problem can be incorporated (the right-to-left means incorporation, i.e., multi-level to one level).
- A hierarchy composition rule is implied which tells how to aggregate the criteria weights between hierarchies:
  ∘ Let ${\bar{\beta }}_{j}^{\left(l+1\right)}$ denote the local weight of a sub-criterion in level *l*+1 w.r.t. its immediately preceding criterion/sub-criterion in level *l* (called the father-criterion);
  ∘ Let $\beta_{j}^{\left(l+1\right)}$ denote the global weight of the sub-criterion w.r.t. the total goal;
  ∘ Let $\beta_{k}^{\left(l\right)}$ denote the father-criterion’s weight w.r.t. the total goal.
  Then, the Hierarchy Composition Rule is
  $$\begin{array}{c} \beta_{j}^{\left(l+1\right)}=\beta_{k}^{\left(l\right)}{\bar{\beta }}_{j}^{\left(l+1\right)}. \end{array}$$

Specifically, the Theorem 3.1 presents a foundation for the PCbHDM in that the weighted geometric mean is used as the aggregation rule.

#### 3.3.3 Desirable traits

The PCbHDM is a hierarchical model that overcomes the aforementioned issues related to the AHP:

1. About the rank reversal: It can be easily proved that the weighted geometric mean aggregation rule used in the PCbHDM is a rank preserved aggregation rule (see [19] or [20]).
  For a quick understanding, we again consider the Dyer’s example but in a more general way:
  - the alternatives A,B,C,D are renamed as *A*_1_,*A*_2_,*A*_3_,*A*_4_, respectively;
  - the criteria a,b,c are renamed as *C*_1_,*C*_2_,*C*_3_, respectively;
  - let *ω*_1_,*ω*_2_,*ω*_3_ denote the criteria weights;
  - let *y*_*ij*_ denote the alternative *A*_*i*_’s local priority under criterion *C*_*j*_, where *i* = 1,2,3,4, and, *j* = 1,2,3 (We’ll give an interpretation on *y*_*ij*_ just below);
  - the *A*_*i*_’s global priorities provided by Saaty’s AHP before and after *A*_4_’s addition are denoted by *AHP*(*A*_*i*_) and *AHP*^+^(*A*_*i*_), respectively;
  - the *A*_*i*_’s global priorities provided by the PCbHDM before and after *A*_4_’s addition are denoted by *PCbHDM*(*A*_*i*_) and *PCbHDM*^+^(*A*_*i*_), respectively.
  Here we give a further interpretation on the notation *y*_*ij*_, namely, the local priority of alternative *A*_*i*_ under criterion *C*_*j*_. In Dyer’s example [4] as well as in Belton-Gear’s example [3], the local priorities are derived from consistent PCMs. It is known that, a consistent PCM corresponds to numerous prority vectors, which are unique up to multiplication by a positive constant. Moreover, any column e.g. the first column of a consistent PCM can be taken as one of its priority vectors, since *ω*_*i*_ = *ω*_*j*_ *p*_*ij*_ [12]. We denote by ${\left(p_{ik}^{j}\right)}_{3\times 3}$ the consistent PCM of alternatives with respect to criterion *C*_*j*_ before the addition of alternative *A*_4_, and by ${\left({\underline{p}}_{ik}^{j}\right)}_{4\times 4}$ the consistent PCM of alternatives with respect to criterion *C*_*j*_ after the addition. Because *A*_4_ is added as an independent alternative, we thus have $p_{ik}^{j}={\underline{p}}_{ik}^{j}$ for *i*,*j*,*k* = 1,2,3. As already mentioned, we can take any column as the local priority vector to be derived from a consistent PCM. For the convenience of discussion, we will take the first column for our purpose, since in Dyer’s example, the first column of ${\left(p_{ik}^{j}\right)}_{3\times 3}$ is included also in the firsrt column of ${\left({\underline{p}}_{ik}^{j}\right)}_{4\times 4}$. Therefore we have: $y_{ij}=p_{i1}^{j}={\underline{p}}_{i1}^{j},i=1,2,3$; and $y_{ij}={\underline{p}}_{i1}^{j},i=4$. For conciseness we just use the notation *y*_*ij*_ to indicate the alternatives’ local priorities in the following illustration.
  Following Saaty’s AHP, we obtain the global priority ratios of *A*_1_ and *A*_2_ before and after *A*_4_ is added:
  $$\begin{array}{c} \frac{AHP(A_{1})}{AHP(A_{2})}=\frac{\omega_{1}\left(y_{11}/\sum_{i=1}^{3}y_{i1}\right)+\omega_{2}\left(y_{12}/\sum_{i=1}^{3}y_{i2}\right)+\omega_{3}\left(y_{13}/\sum_{i=1}^{3}y_{i3}\right)}{\omega_{1}\left(y_{21}/\sum_{i=1}^{3}y_{i1}\right)+\omega_{2}\left(y_{22}/\sum_{i=1}^{3}y_{i2}\right)+\omega_{3}\left(y_{23}/\sum_{i=1}^{3}y_{i3}\right)} \end{array}$$
  and
  $$\begin{array}{c} \frac{AHP^{+}\left(A_{1}\right)}{AHP^{+}\left(A_{2}\right)}=\frac{\omega_{1}\left(y_{11}/\sum_{i=1}^{4}y_{i1}\right)+\omega_{2}\left(y_{12}/\sum_{i=1}^{4}y_{i2}\right)+\omega_{3}\left(y_{13}/\sum_{i=1}^{4}y_{i3}\right)}{\omega_{1}\left(y_{21}/\sum_{i=1}^{4}y_{i1}\right)+\omega_{2}\left(y_{22}/\sum_{i=1}^{4}y_{i2}\right)+\omega_{3}\left(y_{23}/\sum_{i=1}^{4}y_{i3}\right)}. \end{array}$$
  Because Saaty’s AHP uses the above sum-normalization and additive aggregation, when we have $\frac{AHP(A_{1})}{AHP(A_{2})}<1$ we do not definitely have $\frac{AHP^{+}\left(A_{1}\right)}{AHP^{+}\left(A_{2}\right)}<1$, and thus the rank reversal may happen as shown by Dyer’s example.
  Comparatively, if we use the PCbHDM, the ratios are provided as:
  $$\begin{array}{c} \begin{array}{cc} & \frac{PCbHDM(A_{1})}{PCbHDM(A_{2})}=\\ & \;\frac{{\left(y_{11}/\min\left\{y_{i1}|i=1,2,3\right\}\right)}^{\omega_{1}}{\left(y_{12}/\min\left\{y_{i2}|i=1,2,3\right\}\right)}^{\omega_{2}}{\left(y_{13}/\min\left\{y_{i3}|i=1,2,3\right\}\right)}^{\omega_{3}}}{{\left(y_{21}/\min\left\{y_{i1}|i=1,2,3\right\}\right)}^{\omega_{1}}{\left(y_{22}/\min\left\{y_{i2}|i=1,2,3\right\}\right)}^{\omega_{2}}{\left(y_{23}/\min\left\{y_{i3}|i=1,2,3\right\}\right)}^{\omega_{3}}} \end{array} \end{array}$$
  and
  $$\begin{array}{c} \begin{array}{cc} & \frac{PCbHDM^{+}\left(A_{1}\right)}{PCbHDM^{+}\left(A_{2}\right)}=\\ & \;\frac{{\left(y_{11}/\min\left\{y_{i1}|i=1,2,3,4\right\}\right)}^{\omega_{1}}{\left(y_{12}/\min\left\{y_{i2}|i=1,2,3,4\right\}\right)}^{\omega_{2}}{\left(y_{13}/\min\left\{y_{i3}|i=1,2,3,4\right\}\right)}^{\omega_{3}}}{{\left(y_{21}/\min\left\{y_{i1}|i=1,2,3,4\right\}\right)}^{\omega_{1}}{\left(y_{22}/\min\left\{y_{i2}|i=1,2,3,4\right\}\right)}^{\omega_{2}}{\left(y_{23}/\min\left\{y_{i3}|i=1,2,3,4\right\}\right)}^{\omega_{3}}} \end{array}. \end{array}$$
  Clearly, we have $\frac{PCbHDM(A_{1})}{PCbHDM(A_{2})}=\frac{PCbHDM^{+}\left(A_{1}\right)}{PCbHDM^{+}\left(A_{2}\right)}=\frac{{\left(y_{11}\right)}^{\omega_{1}}{\left(y_{12}\right)}^{\omega_{2}}{\left(y_{13}\right)}^{\omega_{3}}}{{\left(y_{21}\right)}^{\omega_{1}}{\left(y_{22}\right)}^{\omega_{2}}{\left(y_{23}\right)}^{\omega_{3}}}$. Therefore, the PCbHDM preserves not only the rank but also the ratio. It is a hierarchical decision model immune to rank reversal.
  It is worth noticing that: (1) Although the above discussion is carried out on a hierarchy with just one criterion-level involved, the verdict that the PCbHDM is a hierarchical decision model immune to rank reversal is also suitable to multiple levels, since the multi-level can be transformed into one-level according to Theorem 3.1; (2) The rank reversal is discussed in the context that, as done in Belton-Gear’s example as well as in Dyer’s example, the involved PCMs are all consistent. Otherwise, rank preservation may not be expected, since inconsistency means judgment contradiction and anything may happen.; (3) The AHP’s rank reversal, which is observed even in perfect consistency cases, is one of the main reasons why many researchers think the AHP as a doubtful method. Comparatively, the PCbHDM can be proven mathematically that the rank reversal is avoided provided that all the involved PCMs are consistent; (4) Rank preservation is not always guaranteed by the PCbHDM when inconsistent PCMs are involved. We shall show this in the illustration section.
2. About the paradox of Saaty’s consistency criterion: Since the acceptable consistency criterion used in the PCbHDM is an ordinal transitivity rather than a cardinal one, thus, the paradox is avoided. Moreover, it has isomorphic counterparts in other cases of PCM [6, 7].
3. About the isomorphic counterparts: The criterion for acceptable consistency, the approach for deriving weights, the hierarchical composition and aggregation rule used in the PCbHDM have their isomorphic counterparts in a fuzzy case [6] or an additive case [7].

In accordance with the above considerations, the present work selects the PCbHDM as the decision making method. The PCbHDM’s desirable traits assure its suitability to the application of this study.

We should note that the main features of PCbHDM are based on previous work of many researchers including, not limited to: the pairwise comparison technique can be traced to at least as early as Thurstone’s work [21]; the hierarchical MCDM model can be traced to Miller’s work [22]; the min-normalization of the multiplicative PCM was suggested in [23]; the weighted geometric aggregation rule was supported by [19, 24] and many others; using the geometric mean to derive weights from a multiplicative PCM was supported by [19], [24, 25], also by many others; the mapping between a multiplicative PCM and a fuzzy PCM was first discussed in [10] and successive studies were also conducted in [9] and [11]; PCM’s isomorphic correspondence from the prospective of abstract algebra was discussed by some researchers as can be seen in [12, 20, 26, 27].

## 4 Illustration, comparison and discussion

This section includes case studies aiming at (1) illustrating the evaluation process using the selected method; (2) showing that the PCbHDM does not guarantee rank preservation when inconsistent PCMs are involved; and (3) showing that the PCbHDM preserves the rank when the involved PCMs are all consistent, while the AHP does not. The example is modified from a real-world example presented in [28]. A graphical software tool has been developed to allow an easy data input and a direct results output. There are 5 alternatives (denoted by *A*_1_, *A*_2_, *A*_3_, *A*_4_ and *A*_5_) to be assessed by five experts comprising a decision group responsible for constructing the criteria structure and determining the input data through a Delphi process. We shall make a comparision between the results provided by the PCbHDM and the AHP. To permit a contrast comparison to be analyzed, we shall list the results calculated by the two methods in same tables.

### 4.1 Illustration

#### 4.1.1 Criteria hierarchy

**Fig 1:**
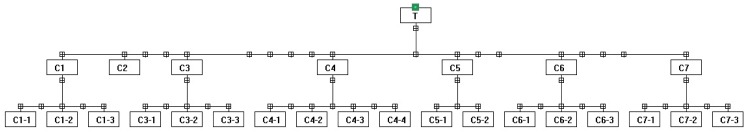
The criteria hierarchy.

Market competitiveness is related to intangible benefits and indirect costs. The evaluation process may involve social, technical, economic, service, and environmental factors (criteria). The decision group identifies seven influential factors including mechanical behavior, price, use cost, operating comfort, service quality, quality and reputation, and safety and environment, which include 18 sub-factors. Table 1 shows the criteria selected for this analysis, and Fig 1 shows the hierarchy structure.

**Table 1:** Criteria and sub-criteria.

Criteria	Sub-criteria
C1: Mechanical behavior	C1-1: Productivity
	C1-2: Exactitude
	C1-3: Versatility
C2: Price	
C3: Use cost	C3-1: Durability
	C3-2: Maintenance cost
	C3-3: Fuel consumption
C4: Operating comfort	C4-1: Operability
	C4-2: Ride comfort
	C4-3: Cab noise
	C4-4: Visibility
C5: Service quality	C5-1: Pre-sales & Sales training
	C5-2: Service convenience
C6: Quality & reputation	C6-1: Lead-edge technologies
	C6-2: Quality
	C6-3: Product appearance
C7: Safety & environment	C7-1: Safety
	C7-2: Noise
	C7-3: Emission

#### 4.1.2 Pairwise comparison data

The input data are presented by the decision group as multiplicative PCMs as shown by the ‘PCM’ columns of Tables 2, 3 and 4.

**Table 2:** PCM of criteria with respect to the goal and local weights.

PCM							Local weights			
							PCbHDM		AHP	
							Consistency	Weights	Consistency	Weights
1	3/2	4/3	8/5	2	3/2	3	acceptable	0.220561	C.R. = 0.000579	0.220497
2/3	1	8/9	9/8	4/3	1	2		0.148163		0.148095
3/4	9/8	1	6/5	3/2	9/8	9/4		0.165421		0.165373
5/8	8/9	5/6	1	5/4	8/9	3/2		0.131510		0.131686
1/2	3/4	2/3	4/5	1	3/4	3/2		0.110281		0.110249
2/3	1	8/9	9/8	4/3	1	2		0.148163		0.148095
1/3	1/2	4/9	2/3	2/3	1/2	1		0.075902		0.076006

**Table 3:** PCMs of sub-criteria with respect to criteria and local weights.

PCM	Local weights			
	PCbHDM		AHP	
	Consistency	Weights	Consistency	Weights
$\begin{array}{c} \text{With}\;\text{respect}\;\text{to}\;\text{C1:}\\ \begin{array}{cccc} & \text{C1-1} & \text{C1-2} & \text{C1-3}\\ \text{C1-1} & 1 & 1 & 2\\ \text{C1-2} & 1 & 1 & 2\\ \text{C1-3} & 1/2 & 1/2 & 1 \end{array} \end{array}$	consistent	$\begin{array}{c} 0.400000\\ 0.400000\\ 0.200000 \end{array}$	consistent	$\begin{array}{c} 0.400000\\ 0.400000\\ 0.200000 \end{array}$
$\begin{array}{c} \text{With}\;\text{respect}\;\text{to}\;\text{C3:}\\ \begin{array}{cccc} & \text{C3-1} & \text{C3-2} & \text{C3-3}\\ \text{C3-1} & 1 & 1 & 3/4\\ \text{C3-2} & 1 & 1 & 3/4\\ \text{C3-3} & 4/3 & 4/3 & 1 \end{array} \end{array}$	consistent	$\begin{array}{c} 0.300000\\ 0.300000\\ 0.400000 \end{array}$	consistent	$\begin{array}{c} 0.300000\\ 0.300000\\ 0.400000 \end{array}$
$\begin{array}{c} \text{With}\;\text{respect}\;\text{to}\;\text{C4:}\\ \begin{array}{ccccc} & \text{C4-1} & \text{C4-2} & \text{C4-3} & \text{C4-3}\\ \text{C4-1} & 1 & 3/2 & 3/2 & 1\\ \text{C4-2} & 2/3 & 1 & 1 & 2/3\\ \text{C4-3} & 2/3 & 1 & 1 & 2/3\\ \text{C4-4} & 1 & 3/2 & 3/2 & 1 \end{array} \end{array}$	consistent	$\begin{array}{c} 0.300000\\ 0.200000\\ 0.200000\\ 0.300000 \end{array}$	consistent	$\begin{array}{c} 0.300000\\ 0.200000\\ 0.200000\\ 0.300000 \end{array}$
$\begin{array}{c} \text{With}\;\text{respect}\;\text{to}\;\text{C5:}\\ \begin{array}{ccc} & \text{C5-1} & \text{C5-2}\\ \text{C5-1} & 1 & 1/4\\ \text{C5-2} & 4 & 1 \end{array} \end{array}$	consistent	$\begin{array}{c} 0.200000\\ 0.800000 \end{array}$	consistent	$\begin{array}{c} 0.200000\\ 0.800000 \end{array}$
$\begin{array}{c} \text{With}\;\text{respect}\;\text{to}\;\text{C6:}\\ \begin{array}{cccc} & \text{C6-1} & \text{C6-2} & \text{C6-3}\\ \text{C6-1} & 1 & 3/5 & 3/2\\ \text{C6-2} & 5/3 & 1 & 5/2\\ \text{C6-3} & 2/3 & 2/5 & 1 \end{array} \end{array}$	consistent	$\begin{array}{c} 0.300000\\ 0.500000\\ 0.200000 \end{array}$	consistent	$\begin{array}{c} 0.300000\\ 0.500000\\ 0.200000 \end{array}$
$\begin{array}{c} \text{With}\;\text{respect}\;\text{to}\;\text{C7:}\\ \begin{array}{cccc} & \text{C7-1} & \text{C7-2} & \text{C7-3}\\ \text{C7-1} & 1 & 4/3 & 4/3\\ \text{C7-2} & 3/4 & 1 & 1\\ \text{C7-3} & 3/4 & 1 & 1 \end{array} \end{array}$	consistent	$\begin{array}{c} 0.400000\\ 0.300000\\ 0.300000 \end{array}$	consistent	$\begin{array}{c} 0.400000\\ 0.300000\\ 0.300000 \end{array}$

**Table 4:** PCMs of alternatives with respect to terminal criteria and local weights.

PCM	Local weights			
	PCbHDM		AHP	
	Consistency	Weights	Consistency	Weights
$\begin{array}{c} \text{With}\;\text{respect}\;\text{to}\;\text{C1-1:}\\ \begin{array}{cccccc} & x_{1} & x_{2} & x_{3} & x_{4} & x_{5}\\ x_{1} & 1 & 1/2 & 1/3 & 1/4 & 1/4\\ x_{2} & 2 & 1 & 2/3 & 1/2 & 1/2\\ x_{3} & 3 & 3/2 & 1 & 3/4 & 3/4\\ x_{4} & 4 & 2 & 4/3 & 1 & 1\\ x_{5} & 4 & 2 & 4/3 & 1 & 1 \end{array} \end{array}$	consistent	$\begin{array}{c} 1.000000\\ 2.000000\\ 2.999998\\ 3.999998\\ 3.999998 \end{array}$	consistent	$\begin{array}{c} 0.071429\\ 0.142857\\ 0.214286\\ 0.285714\\ 0.285714 \end{array}$
$\begin{array}{c} \text{With}\;\text{respect}\;\text{to}\;\text{C1-2:}\\ \begin{array}{cccccc} & x_{1} & x_{2} & x_{3} & x_{4} & x_{5}\\ x_{1} & 1 & 1 & 2/3 & 5/4 & 5/3\\ x_{2} & 1 & 1 & 2/3 & 5/4 & 5/3\\ x_{3} & 3/2 & 3/2 & 1 & 2 & 5/2\\ x_{4} & 4/5 & 4/5 & 1/2 & 1 & 4/3\\ x_{5} & 3/5 & 3/5 & 2/5 & 3/4 & 1 \end{array} \end{array}$	acceptable	$\begin{array}{c} 1.666667\\ 1.666667\\ 2.532479\\ 1.316234\\ 1.000000 \end{array}$	C.R. = 0.000112	$\begin{array}{c} 0.203682\\ 0.203682\\ 0.309545\\ 0.160882\\ 0.122209 \end{array}$
$\begin{array}{c} \text{With}\;\text{respect}\;\text{to}\;\text{C1-3:}\\ \begin{array}{cccccc} & x_{1} & x_{2} & x_{3} & x_{4} & x_{5}\\ x_{1} & 1 & 2 & 3 & 4 & 5\\ x_{2} & 1/2 & 1 & 3/2 & 2 & 5/2\\ x_{3} & 1/3 & 2/3 & 1 & 4/3 & 5/3\\ x_{4} & 1/4 & 1/2 & 3/4 & 1 & 5/4\\ x_{5} & 1/5 & 2/5 & 3/5 & 4/5 & 1 \end{array} \end{array}$	consistent	$\begin{array}{c} 5.000002\\ 2.500002\\ 1.666667\\ 1.250001\\ 1.000000 \end{array}$	consistent	$\begin{array}{c} 0.437956\\ 0.218978\\ 0.145985\\ 0.109489\\ 0.087591 \end{array}$
$\begin{array}{c} \text{With}\;\text{respect}\;\text{to}\;\text{C2:}\\ \begin{array}{cccccc} & x_{1} & x_{2} & x_{3} & x_{4} & x_{5}\\ x_{1} & 1 & 3/2 & 1/2 & 2 & 5/4\\ x_{2} & 2/3 & 1 & 1/3 & 4/3 & 5/6\\ x_{3} & 2 & 3 & 1 & 4 & 5/2\\ x_{4} & 1/2 & 3/4 & 1/4 & 1 & 5/8\\ x_{5} & 4/5 & 6/5 & 2/5 & 8/5 & 1 \end{array} \end{array}$	consistent	$\begin{array}{c} 2.000002\\ 1.333335\\ 4.000002\\ 1.000000\\ 1.600000 \end{array}$	consistent	$\begin{array}{c} 0.201342\\ 0.134228\\ 0.402685\\ 0.100671\\ 0.161074 \end{array}$
$\begin{array}{c} \text{With}\;\text{respect}\;\text{to}\;\text{C3-1:}\\ \begin{array}{cccccc} & x_{1} & x_{2} & x_{3} & x_{4} & x_{5}\\ x_{1} & 1 & 2 & 1/3 & 1/4 & 1/2\\ x_{2} & 1/2 & 1 & 1/6 & 1/8 & 1/4\\ x_{3} & 3 & 6 & 1 & 3/4 & 3/2\\ x_{4} & 4 & 8 & 4/3 & 1 & 2\\ x_{5} & 2 & 4 & 2/3 & 1/2 & 1 \end{array} \end{array}$	consistent	$\begin{array}{c} 2.000000\\ 1.000000\\ 6.000003\\ 8.000003\\ 4.000003 \end{array}$	consistent	$\begin{array}{c} 0.095238\\ 0.047619\\ 0.285714\\ 0.380952\\ 0.190476 \end{array}$
$\begin{array}{c} \text{With}\;\text{respect}\;\text{to}\;\text{C3-2:}\\ \begin{array}{cccccc} & x_{1} & x_{2} & x_{3} & x_{4} & x_{5}\\ x_{1} & 1 & 1/4 & 1/2 & 4 & 1/4\\ x_{2} & 4 & 1 & 2 & 9 & 1\\ x_{3} & 2 & 1/2 & 1 & 8 & 1/2\\ x_{4} & 1/4 & 1/9 & 1/8 & 1 & 1/9\\ x_{5} & 4 & 1 & 2 & 9 & 1 \end{array} \end{array}$	acceptable	$\begin{array}{c} 3.177669\\ 11.329040\\ 6.355338\\ 1.000000\\ 11.329040 \end{array}$	C.R. = 0.011898	$\begin{array}{c} 0.095821\\ 0.341011\\ 0.191641\\ 0.030516\\ 0.341011 \end{array}$
$\begin{array}{c} \text{With}\;\text{respect}\;\text{to}\;\text{C3-3:}\\ \begin{array}{cccccc} & x_{1} & x_{2} & x_{3} & x_{4} & x_{5}\\ x_{1} & 1 & 5 & 5/2 & 1/3 & 1\\ x_{2} & 1/5 & 1 & 1/2 & 1/9 & 1/5\\ x_{3} & 2/5 & 2 & 1 & 1/8 & 2/5\\ x_{4} & 3 & 9 & 8 & 1 & 3\\ x_{5} & 1 & 5 & 5/2 & 1/3 & 1 \end{array} \end{array}$	acceptable	$\begin{array}{c} 4.514397\\ 1.000000\\ 1.782602\\ 12.386738\\ 4.514397 \end{array}$	C.R. = 0.007766	$\begin{array}{c} 0.185472\\ 0.041632\\ 0.073338\\ 0.514086\\ 0.185472 \end{array}$
$\begin{array}{c} \text{With}\;\text{respect}\;\text{to}\;\text{C4-1:}\\ \begin{array}{cccccc} & x_{1} & x_{2} & x_{3} & x_{4} & x_{5}\\ x_{1} & 1 & 4 & 4 & 1/2 & 5\\ x_{2} & 1/4 & 1 & 1 & 1/8 & 5/4\\ x_{3} & 1/4 & 1 & 1 & 1/8 & 5/4\\ x_{4} & 2 & 8 & 8 & 1 & 9\\ x_{5} & 1/5 & 4/5 & 4/5 & 1/9 & 1 \end{array} \end{array}$	acceptable	$\begin{array}{c} 4.895740\\ 1.223936\\ 1.223936\\ 9.587312\\ 1.000000 \end{array}$	C.R. = 0.000298	$\begin{array}{c} 0.272964\\ 0.068241\\ 0.068241\\ 0.534774\\ 0.055781 \end{array}$
$\begin{array}{c} \text{With}\;\text{respect}\;\text{to}\;\text{C4-2:}\\ \begin{array}{cccccc} & x_{1} & x_{2} & x_{3} & x_{4} & x_{5}\\ x_{1} & 1 & 1/4 & 1/2 & 2 & 1/5\\ x_{2} & 4 & 1 & 2 & 8 & 4/5\\ x_{3} & 2 & 1/2 & 1 & 4 & 2/5\\ x_{4} & 1/2 & 1/8 & 1/4 & 1 & 1/9\\ x_{5} & 5 & 5/4 & 5/2 & 9 & 1 \end{array} \end{array}$	acceptable	$\begin{array}{c} 1.958294\\ 7.833181\\ 3.916592\\ 1.000000\\ 9.587306 \end{array}$	C.R. = 0.000298	$\begin{array}{c} 0.080588\\ 0.322354\\ 0.161177\\ 0.041171\\ 0.394710 \end{array}$
$\begin{array}{c} \text{With}\;\text{respect}\;\text{to}\;\text{C4-3:}\\ \begin{array}{cccccc} & x_{1} & x_{2} & x_{3} & x_{4} & x_{5}\\ x_{1} & 1 & 1/2 & 1/5 & 1/4 & 2\\ x_{2} & 2 & 1 & 2/5 & 1/2 & 4\\ x_{3} & 5 & 5/2 & 1 & 5/4 & 9\\ x_{4} & 4 & 2 & 4/5 & 1 & 8\\ x_{5} & 1/2 & 1/4 & 1/9 & 1/8 & 1 \end{array} \end{array}$	acceptable	$\begin{array}{c} 1.958294\\ 3.916592\\ 9.587306\\ 7.833181\\ 1.000000 \end{array}$	C.R. = 0.000298	$\begin{array}{c} 0.080588\\ 0.161177\\ 0.394710\\ 0.322354\\ 0.041171 \end{array}$
$\begin{array}{c} \text{With}\;\text{respect}\;\text{to}\;\text{C4-4:}\\ \begin{array}{cccccc} & x_{1} & x_{2} & x_{3} & x_{4} & x_{5}\\ x_{1} & 1 & 8 & 6 & 1/2 & 4\\ x_{2} & 1/8 & 1 & 3/4 & 1/9 & 1/2\\ x_{3} & 1/6 & 4/3 & 1 & 1/8 & 2/3\\ x_{4} & 2 & 9 & 8 & 1 & 8\\ x_{5} & 1/4 & 2 & 3/2 & 1/8 & 1 \end{array} \end{array}$	acceptable	$\begin{array}{c} 7.130401\\ 1.000000\\ 1.288786\\ 11.720609\\ 1.782599 \end{array}$	C.R. = 0.009159	$\begin{array}{c} 0.309362\\ 0.043626\\ 0.055808\\ 0.513864\\ 0.077341 \end{array}$
$\begin{array}{c} \text{With}\;\text{respect}\;\text{to}\;\text{C5-1:}\\ \begin{array}{cccccc} & x_{1} & x_{2} & x_{3} & x_{4} & x_{5}\\ x_{1} & 1 & 5 & 3 & 2 & 8\\ x_{2} & 1/5 & 1 & 3/5 & 2/5 & 8/5\\ x_{3} & 1/3 & 5/3 & 1 & 2/3 & 8/3\\ x_{4} & 1/2 & 5/2 & 3/2 & 1 & 4\\ x_{5} & 1/8 & 5/8 & 3/8 & 1/4 & 1 \end{array} \end{array}$	consistent	$\begin{array}{c} 8.000011\\ 1.600002\\ 2.666671\\ 4.000005\\ 1.000000 \end{array}$	consistent	$\begin{array}{c} 0.463320\\ 0.092664\\ 0.154440\\ 0.231660\\ 0.057915 \end{array}$
$\begin{array}{c} \text{With}\;\text{respect}\;\text{to}\;\text{C5-2:}\\ \begin{array}{cccccc} & x_{1} & x_{2} & x_{3} & x_{4} & x_{5}\\ x_{1} & 1 & 1/5 & 1/2 & 4 & 1/4\\ x_{2} & 5 & 1 & 5/2 & 9 & 5/4\\ x_{3} & 2 & 2/5 & 1 & 8 & 1/2\\ x_{4} & 1/4 & 1/9 & 1/8 & 1 & 1/8\\ x_{5} & 4 & 4/5 & 2 & 8 & 1 \end{array} \end{array}$	acceptable	$\begin{array}{c} 2.968218\\ 12.650521\\ 5.936440\\ 1.000000\\ 10.335940 \end{array}$	C.R. = 0.020287	$\begin{array}{c} 0.090406\\ 0.384911\\ 0.180811\\ 0.031062\\ 0.312810 \end{array}$
$\begin{array}{c} \text{With}\;\text{respect}\;\text{to}\;\text{C6-1:}\\ \begin{array}{cccccc} & x_{1} & x_{2} & x_{3} & x_{4} & x_{5}\\ x_{1} & 1 & 1/2 & 3 & 4 & 1\\ x_{2} & 2 & 1 & 6 & 8 & 2\\ x_{3} & 1/3 & 1/6 & 1 & 4/3 & 1/3\\ x_{4} & 1/4 & 1/8 & 3/4 & 1 & 1/4\\ x_{5} & 1 & 1/2 & 3 & 4 & 1 \end{array} \end{array}$	consistent	$\begin{array}{c} 4.000003\\ 8.000006\\ 1.333335\\ 1.000000\\ 4.000003 \end{array}$	consistent	$\begin{array}{c} 0.218182\\ 0.436364\\ 0.072727\\ 0.054545\\ 0.218182 \end{array}$
$\begin{array}{c} \text{With}\;\text{respect}\;\text{to}\;\text{C6-2:}\\ \begin{array}{cccccc} & x_{1} & x_{2} & x_{3} & x_{4} & x_{5}\\ x_{1} & 1 & 4 & 4 & 1/4 & 1/2\\ x_{2} & 1/4 & 1 & 1 & 1/9 & 1/8\\ x_{3} & 1/4 & 1 & 1 & 1/9 & 1/8\\ x_{4} & 4 & 9 & 9 & 1 & 2\\ x_{5} & 2 & 8 & 8 & 1/2 & 1 \end{array} \end{array}$	acceptable	$\begin{array}{c} 3.565204\\ 1.000000\\ 1.000000\\ 11.329050\\ 7.130411 \end{array}$	C.R. = 0.011898	$\begin{array}{c} 0.147416\\ 0.041423\\ 0.041423\\ 0.474906\\ 0.294833 \end{array}$
$\begin{array}{c} \text{With}\;\text{respect}\;\text{to}\;\text{C6-3:}\\ \begin{array}{cccccc} & x_{1} & x_{2} & x_{3} & x_{4} & x_{5}\\ x_{1} & 1 & 1/4 & 1/2 & 1/4 & 1/2\\ x_{2} & 4 & 1 & 2 & 1 & 2\\ x_{3} & 2 & 1/2 & 1 & 1/2 & 1\\ x_{4} & 4 & 1 & 2 & 1 & 2\\ x_{5} & 2 & 1/2 & 1 & 1/2 & 1 \end{array} \end{array}$	consistent	$\begin{array}{c} 1.000000\\ 4.000002\\ 2.000002\\ 4.000002\\ 2.000002 \end{array}$	consistent	$\begin{array}{c} 0.076923\\ 0.307692\\ 0.153846\\ 0.307692\\ 0.153846 \end{array}$
$\begin{array}{c} \text{With}\;\text{respect}\;\text{to}\;\text{C7-1:}\\ \begin{array}{cccccc} & x_{1} & x_{2} & x_{3} & x_{4} & x_{5}\\ x_{1} & 1 & 1/5 & 1/2 & 2 & 1/4\\ x_{2} & 5 & 1 & 5/2 & 9 & 5/4\\ x_{3} & 2 & 2/5 & 1 & 4 & 1/2\\ x_{4} & 1/2 & 1/9 & 1/4 & 1 & 1/8\\ x_{5} & 4 & 4/5 & 2 & 8 & 1 \end{array} \end{array}$	acceptable	$\begin{array}{c} 1.958294\\ 9.587306\\ 3.916592\\ 1.000000\\ 7.833181 \end{array}$	C.R. = 0.000298	$\begin{array}{c} 0.080588\\ 0.394710\\ 0.161177\\ 0.041171\\ 0.322354 \end{array}$
$\begin{array}{c} \text{With}\;\text{respect}\;\text{to}\;\text{C7-2:}\\ \begin{array}{cccccc} & x_{1} & x_{2} & x_{3} & x_{4} & x_{5}\\ x_{1} & 1 & 2 & 1/2 & 1 & 2\\ x_{2} & 1/2 & 1 & 1/4 & 1/2 & 1\\ x_{3} & 2 & 4 & 1 & 2 & 4\\ x_{4} & 1 & 2 & 1/2 & 1 & 2\\ x_{5} & 1/2 & 1 & 1/4 & 1/2 & 1 \end{array} \end{array}$	consistent	$\begin{array}{c} 2.000000\\ 1.000000\\ 4.000002\\ 2.000000\\ 1.000000 \end{array}$	consistent	$\begin{array}{c} 0.200000\\ 0.100000\\ 0.400000\\ 0.200000\\ 0.100000 \end{array}$
$\begin{array}{c} \text{With}\;\text{respect}\;\text{to}\;\text{C7-3:}\\ \begin{array}{cccccc} & x_{1} & x_{2} & x_{3} & x_{4} & x_{5}\\ x_{1} & 1 & 4 & 2 & 1/2 & 4\\ x_{2} & 1/4 & 1 & 1/2 & 1/8 & 1\\ x_{3} & 1/2 & 2 & 1 & 1/4 & 2\\ x_{4} & 2 & 8 & 4 & 1 & 8\\ x_{5} & 1/4 & 1 & 1/2 & 1/8 & 1 \end{array} \end{array}$	consistent	$\begin{array}{c} 4.000002\\ 1.000000\\ 2.000002\\ 8.000005\\ 1.000000 \end{array}$	consistent	$\begin{array}{c} 0.250000\\ 0.062500\\ 0.125000\\ 0.500000\\ 0.062500 \end{array}$

#### 4.1.3 Local weights and global weights

Now, we find the overall priorities of alternatives by implementing the PCbHDM procedure described in Subsection 3.3.1:

- The PCM’s acceptable consistency is tested by the criterion of inequality Eq (1), the local weighs are derived by Eq (2) and are provided in Tables 2, 3 and 4. We should note that the criterion’s local weights are sum-normalized, and, the alternative’s local weights are min-normalized.
- By using the Hierarchy Composition Rule of Eq (3), we obtain the global weights of the terminal criteria, say {C1-1,C1-2,C1-3,C2,C3-1,C3-2,C3-3,C4-1,C4-2,C4-3,C4-4,C5-1,C5-2,C6-1,C6-2,C6-3,C7-1,C7-2,C7-3}, as: 0.088224, 0.088224, 0.044112, 0.148163, 0.049626, 0.049626, 0.066168, 0.039453, 0.026302, 0.026302, 0.039453, 0.022056, 0.088225, 0.044449, 0.074081, 0.029633, 0.030361, 0.022771, 0.022771.
- From the aggregation rule of Eq (4), we obtain the alternatives’ overall priorities as: 2.588777, 2.325968, 2.829265, 2.667508, 2.884724.

Thus, the alternative’s ranking produced by the PCbHDM is:

$$\begin{array}{c} A_{5}≻A_{3}≻A_{4}≻A_{1}≻A_{2}. \end{array}$$ (5-*a*)

#### 4.1.4 Comparison

To highlight the procedure of the suggested model and to make a comparison, we list the results following the Saaty’s original AHP. They are also included in Tables 2–4. By Saaty’s original AHP, the alternatives’ overall priorities are obtained as: 0.176182, 0.182554, 0.207429, 0.236312, 0.197525, which indicate a final ranking

$$\begin{array}{c} A_{4}≻A_{3}≻A_{5}≻A_{2}≻A_{1}. \end{array}$$ (5-*b*)

Now, we further investigate what will happen if we delete an existing alternative while the comparison results between the other ones remain unchanged. For instance, we delete the alternative *A*_4_, and therefore, the PCMs of alternatives with respect to the terminal criteria are obtained by deleting the 4th rows and the 4th columns of the PCMs contained in Table 4.

The PCbHDM gives an overall priorities as: 2.064422, 1.855985, 2.228957, 2.341745, which indicate a final ranking

$$\begin{array}{c} A_{5}≻A_{3}≻A_{1}≻A_{2}. \end{array}$$ (6-*a*)

In contrast, the original AHP produces an overall priorities as: 0.251942, 0.219854, 0.260724, 0.267482, which indicate a final ranking as

$$\begin{array}{c} A_{5}≻A_{3}≻A_{1}≻A_{2}. \end{array}$$ (6-*b*)

Comparing Eqs (5-a) and (6-a), we know that the rank is preserved when the PCbHDM is used. As indicated by Eqs (5-b) and (6-b), rank reversals are observed for the original AHP, since the rank of *A*_1_ and *A*_2_ and the rank of *A*_3_ and *A*_5_ are reversed before and after the alternative *A*_4_ is deleted.

Let us further investigate what happens if repeating the test for each single alternatives, *A*_1_,*A*_2_,*A*_3_ and *A*_5_. We provide the results in Table 5.

**Table 5:** Rank order for each single deletion.

Deleted alternative	AHP		PCbHDM	
	Rank order	Preserve or not	Rank order	Preserve or not
None	*A*_4_≻ *A*_3_≻ *A*_5_≻ *A*_2_≻ *A*_1_	–	*A*_5_≻ *A*_3_≻ *A*_4_≻ *A*_1_≻ *A*_2_	–
*A*_1_	*A*_4_≻ *A*_5_≻ *A*_2_≻ *A*_3_	No	*A*_5_≻ *A*_4_≻ *A*_3_≻ *A*_2_	No
*A*_2_	*A*_4_≻ *A*_5_≻ *A*_3_≻ *A*_1_	No	*A*_5_≻ *A*_4_≻ *A*_3_≻ *A*_1_	No
*A*_3_	*A*_4_≻ *A*_5_≻ *A*_2_≻ *A*_1_	Yes	*A*_5_≻ *A*_4_≻ *A*_1_≻ *A*_2_	Yes
*A*_4_	*A*_5_≻ *A*_3_≻ *A*_1_≻ *A*_2_	No	*A*_5_≻ *A*_3_≻ *A*_1_≻ *A*_2_	Yes
*A*_5_	*A*_4_≻ *A*_3_≻ *A*_2_≻ *A*_1_	Yes	*A*_3_≻ *A*_4_≻ *A*_1_≻ *A*_2_	Yes

As indicated by Table 5, the PCbHDM does not always preserve the rank. The reason lies in that some of the involved PCMs are inconsistent. Indeed, if all the PCMs are consistent, the PCbHDM will, as already proved in subsection 3.3.3, always preserve the rank. We show this below by providing the earlier hierarchy with consistent PCMs.

### 4.2 Rank preservation test with PCMs all consistent

In this subsection, we conduct a rank preservation test on the previous hierarchy with all the PCMs consistent and then we give a discussion. The data are prepared by modifying the inconsistent PCMs based upon their respective first columns. For sake of conciseness, the input data and the intermediate results are relegated to the file of supplementary materials for this paper (S1 and S2 Files) and here we just list the final results and give a discussion.

#### 4.2.1 Results

After converting the inconsistent PCMs into consistent ones based on their respective first columns, and doing the rank preservation test for each single alternative, we obtain the results by the AHP and by the PCbHDM as shown in Tables 6 and 7.

**Table 6:** Rank order with a single alternative deleted (consistent case).

Deleted alternative	AHP		PCbHDM	
	Rank order	Preserve or not	Rank order	Preserve or not
None	*A*_4_≻ *A*_5_≻ *A*_2_≻ *A*_3_≻ *A*_1_	–	*A*_5_≻ *A*_4_≻ *A*_3_≻ *A*_1_≻ *A*_2_	–
*A*_1_	*A*_4_≻ *A*_5_≻ *A*_2_≻ *A*_3_	Yes	*A*_5_≻ *A*_4_≻ *A*_3_≻ *A*_2_	Yes
*A*_2_	*A*_4_≻ *A*_5_≻ *A*_3_≻ *A*_1_	Yes	*A*_5_≻ *A*_4_≻ *A*_3_≻ *A*_1_	Yes
*A*_3_	*A*_4_≻ *A*_5_≻ *A*_2_≻ *A*_1_	Yes	*A*_5_≻ *A*_4_≻ *A*_1_≻ *A*_2_	Yes
*A*_4_	*A*_5_≻ *A*_1_≻ *A*_3_≻ *A*_2_	No	*A*_5_≻ *A*_3_≻ *A*_1_≻ *A*_2_	Yes
*A*_5_	*A*_4_≻ *A*_2_≻ *A*_3_≻ *A*_1_	Yes	*A*_4_≻ *A*_3_≻ *A*_1_≻ *A*_2_	Yes

**Table 7:** Global weights with a single alternative deleted (consistent case).

Deleted alternative	Global weights	
	AHP	PCbHDM
None	*A*_1_ : 0:175333	*A*_1_ : 2:756191
	*A*_2_ : 0:187937	*A*_2_ : 2:601603
	*A*_3_ : 2:807889	*A*_3_ : 2:807889
	*A*_4_ : 0:255357	*A*_4_ : 3:104646
	*A*_5_ : 0:200337	*A*_5_ : 3:214869
*A*_1_	*A*_2_ : 0:229391	*A*_2_ : 2:302583
	*A*_3_ : 0:220757	*A*_3_ : 2:485160
	*A*_4_ : 0:315869	*A*_4_ : 2:747810
	*A*_5_ : 0:233982	*A*_5_ : 2:845364
*A*_2_	*A*_1_ : 0:216874	*A*_1_ : 2:513930
	*A*_3_ : 0:228857	*A*_3_ : 2:561084
	*A*_4_ : 0:287943	*A*_4_ : 2:831758
	*A*_5_ : 0:266325	*A*_5_ : 2:932293
*A*_3_	*A*_1_ : 0:214649	*A*_1_ : 2:756190
	*A*_2_ : 0:233211	*A*_2_ : 2:601601
	*A*_4_ : 0:306330	*A*_4_ : 3:104647
	*A*_5_ : 0:245809	*A*_5_ : 3:214868
*A*_4_	*A*_1_ : 0:257431	*A*_1_ : 2:134666
	*A*_2_ : 0:226750	*A*_2_ : 2:014938
	*A*_3_ : 0:239426	*A*_3_ : 2:174706
	*A*_5_ : 0:276392	*A*_5_ : 2:489912
*A*_5_	*A*_1_ : 0:209352	*A*_1_ : 2:535070
	*A*_2_ : 0:250243	*A*_2_ : 2:392883
	*A*_3_ : 0:228092	*A*_3_ : 2:582620
	*A*_4_ : 0:312312	*A*_4_ : 2:855571

#### 4.2.2 Discussion

As shown by Table 6, the PCbHDM preserves the rank when the PCMs are all consistent. However, the AHP does not preserve the rank. Moreover, as indicated by Table 7, the PCbHDM also preserves the ratios, e.g., the ratios of the global weights of *A*_1_ and *A*_2_ before and after the *A*_4_’s deletion are

$$\begin{array}{c} \frac{2.756191}{2.601603}=1.059420288\;\text{and}\;\frac{2.134666}{2.014938}=1.059420191, \end{array}$$

respectively. These two ratios are identical to each other with a precision of 10^−6^. The observation that the AHP behaves rank reversal even in consistent case confirms what the Dyer’s paper and the Triantaphyllou’s paper already illustrate: ‘the ranking produced by Saaty’s AHP is arbitrary ‘ [4], and ‘we may never know the exact ranking if we use the original AHP’ [5].

## 5 Concluding remarks

This paper has described how a hierarchical decision-making technique, specifically pairwise-comparison-based hierarchical decision model (PCbHDM) could be used to make decisions regarding market competitiveness evaluations of mechanical equipment. The PCbHDM is a hierarchical decision model of the property of rank preservation. This desirable trait has been proved mathematically and shown in illustration through comparison. It is hoped that the PCbHDM finds applications in other multi-criteria decision environments.

When to cope with the AHP’s drawbacks, the PCbHDM concentrates on the rank reversal, the method for extracting local priotities and the criterion for acceptable consistency. There are many improved AHP models to overcome some other deficiencies of the Saaty’s AHP. To compensate for expert subjectivity encountered in factor comparisons, the M-AHP attempts first to set the boundary of the sampling space of the relevant problem based on the maximum factor scores at the initial stage and then to ask the experts to express the instant factor scores within the already set range [29]. Because the AHP uses a discrete scale of one to nine which cannot sufficiently handle the uncertainty and ambiguity when deciding the priorities of different attributes, the fuzzy AHP extends the AHP to the situation where the expert cannot provide an exact number from the given scale by replacing the exact ratio by a fuzzy ratio [30, 31]. The D-AHP model is developed from the AHP method by extension with D numbers preference relations [32]. The D numbers are proposed by Deng [33]as a new effective and feasible tool for representing uncertain information. The D numbers preference relation overcomes the deficiency of fuzzy preference relation (e.g., there are 10 experts to assess two alternatives A and B. If 8 of the experts think that A is preferred to B with a degree of 0.7, while the others also think that A is preferred to B but with a degree of 0.6. In this case, the preference degree of A over B can be described by a D number, that is {(0.7,0.8),(0.6,0.2)}) [32], and thus the D-AHP method can handle the uncertain information more effectively than the traditional AHP and Dempster-Shafer theory. In our future work, the advanced techniques used in these improved AHP models will be incorporated into the PCbHDM to make it compatible with various complex decision situations.

We should emphasize once more that the PCbHDM’s rank preservation is proved under the condition that all the PCMs are consistent. If this requirement is not satisfied, rank reversals may happen. Of course this phenomenon will never impair the PCbHDM’s superiority to Saaty’AHP, since the rank reversal happens to Saaty’s AHP even in the consisten case. Anyway, a problem arises, that is, to preserve the rank, should the input PCMs be rectified as consistent as possible before we use the PCbHDM? We’d like to leave it open.

## Supporting Information

S1 File Data for AHP in Inconsistent cases. (PDF)

S2 File Data for PCbHDM in Inconsistent cases. (PDF)
