# Supplementary material for: Market Competitiveness Evaluation of Mechanical Equipment with a Pairwise Comparisons Hierarchical Model
Source: PLoS One. 2016 Jan 19;11(1):e0146862. doi: 10.1371/journal.pone.0146862 (PMC4718694; doi:10.1371/journal.pone.0146862)
Supplement: S1 File — (PDF) [file pone.0146862.s001.pdf]

The hierarchy structure:

7 T TOP C1 1 C2 0 C3 1 C4 1 C5 1 C6 1 C7 1

3 C1 T C1-1 0 C1-2 0 C1-3 0

3 C3 T C3-1 0 C3-2 0 C3-3 0

4 C4 T C4-1 0 C4-2 0 C4-3 0 C4-4 0

2 C5 T C5-1 0 C5-2 0

3 C6 T C6-1 0 C6-2 0 C6-3 0

3 C7 T C7-1 0 C7-2 0 C7-3 0

The number of alternatives:5

Criterion: T; Sub-criterion: C1 C2 C3 C4 C5 C6 C7

PCM of Sub-criterion w.r.t. Criterion:

1.000000	1.500000	1.333333	1.600000	2.000000	1.500000	3.000000
0.666667	1.000000	0.888889	1.066667	1.333333	1.000000	2.000000
0.750000	1.125000	1.000000	1.200000	1.500000	1.125000	2.250000
0.625000	0.937500	0.833333	1.000000	1.250000	0.937500	1.875000
0.500000	0.750000	0.666667	0.800000	1.000000	0.750000	1.500000
0.666667	1.000000	0.888889	1.066667	1.333333	1.000000	2.000000
0.333333	0.500000	0.444444	0.533333	0.666667	0.500000	1.000000

The principal eigenvalue is: 7.000000

The Consistency Ratio is: 0.000000

The PCM HAS acceptable consistency.

Local weights of the sub-criterion (eigenvector):

0.220183 0.146789 0.165138 0.137615 0.110092 0.146789 0.073394

Criterion: C1; Sub-criterion: C1-1 C1-2 C1-3

PCM of Sub-criterion w.r.t. Criterion:

1.000000	1.000000	2.000000
1.000000	1.000000	2.000000
0.500000	0.500000	1.000000

The principal eigenvalue is: 3.000000

The Consistency Ratio is: 0.000000

The PCM HAS acceptable consistency.

Local weights of the sub-criterion (eigenvector):

0.400000 0.400000 0.200000

Criterion: C3; Sub-criterion: C3-1 C3-2 C3-3

PCM of Sub-criterion w.r.t. Criterion:

1.000000	1.000000	0.750000
1.000000	1.000000	0.750000
1.333333	1.333333	1.000000

The principal eigenvalue is: 3.000000

The Consistency Ratio is: 0.000000

The PCM HAS acceptable consistency.

Local weights of the sub-criterion (eigenvector):

0.300000 0.300000 0.400000

Criterion: C4; Sub-criterion: C4-1 C4-2 C4-3 C4-4

PCM of Sub-criterion w.r.t. Criterion:

1.000000	1.500000	1.500000	1.000000
0.666667	1.000000	1.000000	0.666667
0.666667	1.000000	1.000000	0.666667
1.000000	1.500000	1.500000	1.000000

The principal eigenvalue is: 4.000000

The Consistency Ratio is: 0.000000

The PCM HAS acceptable consistency.

Local weights of the sub-criterion (eigenvector):

0.300000 0.200000 0.200000 0.300000

Criterion: C5; Sub-criterion: C5-1 C5-2

PCM of Sub-criterion w.r.t. Criterion:

1.000000 0.250000

4.000000 1.000000

The principal eigenvalue is: 2.000000

The Consistency Ratio is: 0.000000

The PCM HAS acceptable consistency.

Local weights of the sub-criterion (eigenvector):

0.200000 0.800000

Criterion: C6; Sub-criterion: C6-1 C6-2 C6-3

PCM of Sub-criterion w.r.t. Criterion:

1.000000 0.600000 1.500000

1.666667 1.000000 2.500000

0.666667 0.400000 1.000000

The principal eigenvalue is: 3.000000

The Consistency Ratio is: 0.000000

The PCM HAS acceptable consistency.

Local weights of the sub-criterion (eigenvector):

0.300000 0.500000 0.200000

Criterion: C7; Sub-criterion: C7-1 C7-2 C7-3

PCM of Sub-criterion w.r.t. Criterion:

1.000000 1.333333 1.333333

0.750000 1.000000 1.000000

0.750000 1.000000 1.000000

The principal eigenvalue is: 3.000000

The Consistency Ratio is: 0.000000

The PCM HAS acceptable consistency.

Local weights of the sub-criterion (eigenvector):

0.400000   0.300000   0.300000

Alternatives: A1   A2   A3   A4   A5

Leaf criteria: C2   C1-1   C1-2   C1-3   C3-1   C3-2   C3-3   C4-1   C4-2   C4-3   C4-4   C5-1   C5-2   C6-1   C6-2   C6-3   C7-1   C7-2   C7-3

PCM for Alternatives w.r.t. leaf criterion C2

1.000000	0.500000	0.333333	0.250000	0.250000
2.000000	1.000000	0.666667	0.500000	0.500000
3.000000	1.500000	1.000000	0.750000	0.750000
4.000000	2.000000	1.333333	1.000000	1.000000
4.000000	2.000000	1.333333	1.000000	1.000000

The principal eigenvalue is: 5.000000

The Consistency Ratio is: 0.000000

The PCM HAS acceptable consistency.

Local weights of the alternatives (eigenvector):

0.071429   0.142857   0.214286   0.285714   0.285714

PCM for Alternatives w.r.t. leaf criterion C1-1

1.000000	1.000000	0.666667	1.250000	1.666667
1.000000	1.000000	0.666667	1.250000	1.666667
1.500000	1.500000	1.000000	1.875000	2.500000
0.800000	0.800000	0.533333	1.000000	1.333333
0.600000	0.600000	0.400000	0.750000	1.000000

The principal eigenvalue is: 5.000000

The Consistency Ratio is: 0.000000

The PCM HAS acceptable consistency.

Local weights of the alternatives (eigenvector):

0.204082 0.204082 0.306122 0.163265 0.122449

PCM for Alternatives w.r.t. leaf criterion C1-2

1.000000	2.000000	3.000000	4.000000	5.000000
0.500000	1.000000	1.500000	2.000000	2.500000
0.333333	0.666667	1.000000	1.333333	1.666667
0.250000	0.500000	0.750000	1.000000	1.250000
0.200000	0.400000	0.600000	0.800000	1.000000

The principal eigenvalue is: 5.000000

The Consistency Ratio is: 0.000000

The PCM HAS acceptable consistency.

Local weights of the alternatives (eigenvector):

0.437956   0.218978   0.145985   0.109489   0.087591

PCM for Alternatives w.r.t. leaf criterion C1-3

1.000000	1.500000	0.500000	2.000000	1.250000
0.666667	1.000000	0.333333	1.333333	0.833333
2.000000	3.000000	1.000000	4.000000	2.500000
0.500000	0.750000	0.250000	1.000000	0.625000
0.800000	1.200000	0.400000	1.600000	1.000000

The principal eigenvalue is: 5.000000

The Consistency Ratio is: 0.000000

The PCM HAS acceptable consistency.

Local weights of the alternatives (eigenvector):

0.201342   0.134228   0.402685   0.100671   0.161074

PCM for Alternatives w.r.t. leaf criterion C3-1

1.000000	2.000000	0.333333	0.250000	0.500000
0.500000	1.000000	0.166667	0.125000	0.250000
3.000000	6.000000	1.000000	0.750000	1.500000
4.000000	8.000000	1.333333	1.000000	2.000000
2.000000	4.000000	0.666667	0.500000	1.000000

The principal eigenvalue is: 5.000000

The Consistency Ratio is: 0.000000

The PCM HAS acceptable consistency.

Local weights of the alternatives (eigenvector):

0.095238 0.047619 0.285714 0.380952 0.190476

PCM for Alternatives w.r.t. leaf criterion C3-2

1.000000	0.250000	0.500000	4.000000	0.250000
4.000000	1.000000	2.000000	16.000000	1.000000
2.000000	0.500000	1.000000	8.000000	0.500000
0.250000	0.062500	0.125000	1.000000	0.062500
4.000000	1.000000	2.000000	16.000000	1.000000

The principal eigenvalue is: 5.000000

The Consistency Ratio is: 0.000000

The PCM HAS acceptable consistency.

Local weights of the alternatives (eigenvector):

0.088889 0.355556 0.177778 0.022222 0.355556

PCM for Alternatives w.r.t. leaf criterion C3-3

1.000000	5.000000	2.500000	0.333333	1.000000
0.200000	1.000000	0.500000	0.066667	0.200000
0.400000	2.000000	1.000000	0.133333	0.400000
3.000000	15.000000	7.500000	1.000000	3.000000
1.000000	5.000000	2.500000	0.333333	1.000000

The principal eigenvalue is: 5.000000

The Consistency Ratio is: 0.000000

The PCM HAS acceptable consistency.

Local weights of the alternatives (eigenvector):

0.178571 0.035714 0.071429 0.535714 0.178571

PCM for Alternatives w.r.t. leaf criterion C4-1

1.000000	4.000000	4.000000	0.500000	5.000000
0.250000	1.000000	1.000000	0.125000	1.250000
0.250000	1.000000	1.000000	0.125000	1.250000
2.000000	8.000000	8.000000	1.000000	10.000000
0.200000	0.800000	0.800000	0.100000	1.000000

The principal eigenvalue is: 5.000000

The Consistency Ratio is: 0.000000

The PCM HAS acceptable consistency.

Local weights of the alternatives (eigenvector):

0.270270 0.067568 0.067568 0.540541 0.054054

PCM for Alternatives w.r.t. leaf criterion C4-2

1.000000	0.250000	0.500000	2.000000	0.200000
4.000000	1.000000	2.000000	8.000000	0.800000
2.000000	0.500000	1.000000	4.000000	0.400000
0.500000	0.125000	0.250000	1.000000	0.100000
5.000000	1.250000	2.500000	10.000000	1.000000

The principal eigenvalue is: 5.000000

The Consistency Ratio is: 0.000000

The PCM HAS acceptable consistency.

Local weights of the alternatives (eigenvector):

0.080000 0.320000 0.160000 0.040000 0.400000

PCM for Alternatives w.r.t. leaf criterion C4-3

1.000000	0.500000	0.200000	0.250000	2.000000
2.000000	1.000000	0.400000	0.500000	4.000000
5.000000	2.500000	1.000000	1.250000	10.000000
4.000000	2.000000	0.800000	1.000000	8.000000
0.500000	0.250000	0.100000	0.125000	1.000000

The principal eigenvalue is: 5.000000

The Consistency Ratio is: 0.000000

The PCM HAS acceptable consistency.

Local weights of the alternatives (eigenvector):

0.080000 0.160000 0.400000 0.320000 0.040000

PCM for Alternatives w.r.t. leaf criterion C4-4

1.000000	8.000000	6.000000	0.500000	4.000000

0.125000	1.000000	0.750000	0.062500	0.500000
0.166667	1.333333	1.000000	0.083333	0.666667
2.000000	16.000000	12.000000	1.000000	8.000000
0.250000	2.000000	1.500000	0.125000	1.000000

The principal eigenvalue is: 5.000000

The Consistency Ratio is: 0.000000

The PCM HAS acceptable consistency.

Local weights of the alternatives (eigenvector):

0.282353 0.035294 0.047059 0.564706 0.070588

PCM for Alternatives w.r.t. leaf criterion C5-1

1.000000	5.000000	3.000000	2.000000	8.000000
0.200000	1.000000	0.600000	0.400000	1.600000
0.333333	1.666667	1.000000	0.666667	2.666667
0.500000	2.500000	1.500000	1.000000	4.000000
0.125000	0.625000	0.375000	0.250000	1.000000

The principal eigenvalue is: 5.000000

The Consistency Ratio is: 0.000000

The PCM HAS acceptable consistency.

Local weights of the alternatives (eigenvector):

0.463320 0.092664 0.154440 0.231660 0.057915

PCM for Alternatives w.r.t. leaf criterion C5-2

1.000000	0.200000	0.500000	4.000000	0.250000
5.000000	1.000000	2.500000	20.000000	1.250000
2.000000	0.400000	1.000000	8.000000	0.500000
0.250000	0.050000	0.125000	1.000000	0.062500
4.000000	0.800000	2.000000	16.000000	1.000000

The principal eigenvalue is: 5.000000

The Consistency Ratio is: 0.000000

The PCM HAS acceptable consistency.

Local weights of the alternatives (eigenvector):

0.081633 0.408163 0.163265 0.020408 0.326531

PCM for Alternatives w.r.t. leaf criterion C6-1

1.000000	0.500000	3.000000	4.000000	1.000000
2.000000	1.000000	6.000000	8.000000	2.000000

0.333333	0.166667	1.000000	1.333333	0.333333
0.250000	0.125000	0.750000	1.000000	0.250000
1.000000	0.500000	3.000000	4.000000	1.000000

The principal eigenvalue is: 5.000000

The Consistency Ratio is: 0.000000

The PCM HAS acceptable consistency.

Local weights of the alternatives (eigenvector):

0.218182 0.436364 0.072727 0.054545 0.218182

PCM for Alternatives w.r.t. leaf criterion C6-2

1.000000	4.000000	4.000000	0.250000	0.500000
0.250000	1.000000	1.000000	0.062500	0.125000
0.250000	1.000000	1.000000	0.062500	0.125000
4.000000	16.000000	16.000000	1.000000	2.000000
2.000000	8.000000	8.000000	0.500000	1.000000

The principal eigenvalue is: 5.000000

The Consistency Ratio is: 0.000000

The PCM HAS acceptable consistency.

Local weights of the alternatives (eigenvector):

0.133333 0.033333 0.033333 0.533333 0.266667

PCM for Alternatives w.r.t. leaf criterion C6-3

1.000000	0.250000	0.500000	0.250000	0.500000
4.000000	1.000000	2.000000	1.000000	2.000000
2.000000	0.500000	1.000000	0.500000	1.000000
4.000000	1.000000	2.000000	1.000000	2.000000
2.000000	0.500000	1.000000	0.500000	1.000000

The principal eigenvalue is: 5.000000

The Consistency Ratio is: 0.000000

The PCM HAS acceptable consistency.

Local weights of the alternatives (eigenvector):

0.076923 0.307692 0.153846 0.307692 0.153846

PCM for Alternatives w.r.t. leaf criterion C7-1

1.000000	0.200000	0.500000	2.000000	0.250000
5.000000	1.000000	2.500000	10.000000	1.250000
2.000000	0.400000	1.000000	4.000000	0.500000

0.500000	0.100000	0.250000	1.000000	0.125000
4.000000	0.800000	2.000000	8.000000	1.000000

The principal eigenvalue is: 5.000000

The Consistency Ratio is: 0.000000

The PCM HAS acceptable consistency.

Local weights of the alternatives (eigenvector):

0.080000   0.400000   0.160000   0.040000   0.320000

PCM for Alternatives w.r.t. leaf criterion C7-2

1.000000	2.000000	0.500000	1.000000	2.000000
0.500000	1.000000	0.250000	0.500000	1.000000
2.000000	4.000000	1.000000	2.000000	4.000000
1.000000	2.000000	0.500000	1.000000	2.000000
0.500000	1.000000	0.250000	0.500000	1.000000

The principal eigenvalue is: 5.000000

The Consistency Ratio is: 0.000000

The PCM HAS acceptable consistency.

Local weights of the alternatives (eigenvector):

0.200000   0.100000   0.400000   0.200000   0.100000

PCM for Alternatives w.r.t. leaf criterion C7-3

1.000000	4.000000	2.000000	0.500000	4.000000
0.250000	1.000000	0.500000	0.125000	1.000000
0.500000	2.000000	1.000000	0.250000	2.000000
2.000000	8.000000	4.000000	1.000000	8.000000
0.250000	1.000000	0.500000	0.125000	1.000000

The principal eigenvalue is: 5.000000

The Consistency Ratio is: 0.000000

The PCM HAS acceptable consistency.

Local weights of the alternatives (eigenvector):

0.250000 0.062500 0.125000 0.500000 0.062500

Global weights of the leaf criterion:

0.146789 0.088073 0.088073 0.044037 0.049541 0.049541 0.066055 0.041284
0.027523 0.027523 0.041284 0.022018 0.088074 0.044037 0.073395 0.029358
0.029358 0.022018 0.022018

Alternatives' global weights:

0.175333 0.187937 0.181034 0.255357 0.200337

The hierarchy structure:

7 T TOP C1 1 C2 0 C3 1 C4 1 C5 1 C6 1 C7 1

3 C1 T C1-1 0 C1-2 0 C1-3 0

3 C3 T C3-1 0 C3-2 0 C3-3 0

4 C4 T C4-1 0 C4-2 0 C4-3 0 C4-4 0

2 C5 T C5-1 0 C5-2 0

3 C6 T C6-1 0 C6-2 0 C6-3 0

3 C7 T C7-1 0 C7-2 0 C7-3 0

The number of alternatives:4

Criterion: T; Sub-criterion: C1 C2 C3 C4 C5 C6 C7

PCM of Sub-criterion w.r.t. Criterion:

1.000000	1.500000	1.333333	1.600000	2.000000	1.500000	3.000000
0.666667	1.000000	0.888889	1.066667	1.333333	1.000000	2.000000
0.750000	1.125000	1.000000	1.200000	1.500000	1.125000	2.250000
0.625000	0.937500	0.833333	1.000000	1.250000	0.937500	1.875000
0.500000	0.750000	0.666667	0.800000	1.000000	0.750000	1.500000
0.666667	1.000000	0.888889	1.066667	1.333333	1.000000	2.000000
0.333333	0.500000	0.444444	0.533333	0.666667	0.500000	1.000000

The principal eigenvalue is: 7.000000

The Consistency Ratio is: 0.000000

The PCM HAS acceptable consistency.

Local weights of the sub-criterion (eigenvector):

0.220183 0.146789 0.165138 0.137615 0.110092 0.146789 0.073394

Criterion: C1; Sub-criterion: C1-1 C1-2 C1-3

PCM of Sub-criterion w.r.t. Criterion:

1.000000 1.000000 2.000000

1.000000 1.000000 2.000000

0.500000 0.500000 1.000000

The principal eigenvalue is: 3.000000

The Consistency Ratio is: 0.000000

The PCM HAS acceptable consistency.

Local weights of the sub-criterion (eigenvector):

0.400000 0.400000 0.200000

Criterion: C3;   Sub-criterion: C3-1   C3-2   C3-3

PCM of Sub-criterion w.r.t. Criterion:

1.000000	1.000000	0.750000
1.000000	1.000000	0.750000
1.333333	1.333333	1.000000

The principal eigenvalue is: 3.000000

The Consistency Ratio is: 0.000000

The PCM HAS acceptable consistency.

Local weights of the sub-criterion (eigenvector):

0.300000   0.300000   0.400000

Criterion: C4;   Sub-criterion: C4-1   C4-2   C4-3   C4-4

PCM of Sub-criterion w.r.t. Criterion:

1.000000	1.500000	1.500000	1.000000
0.666667	1.000000	1.000000	0.666667
0.666667	1.000000	1.000000	0.666667
1.000000	1.500000	1.500000	1.000000

The principal eigenvalue is: 4.000000

The Consistency Ratio is: 0.000000

The PCM HAS acceptable consistency.

Local weights of the sub-criterion (eigenvector):

0.300000 0.200000 0.200000 0.300000

Criterion: C5; Sub-criterion: C5-1 C5-2

PCM of Sub-criterion w.r.t. Criterion:

1.000000 0.250000

4.000000 1.000000

The principal eigenvalue is: 2.000000

The Consistency Ratio is: 0.000000

The PCM HAS acceptable consistency.

Local weights of the sub-criterion (eigenvector):

0.200000 0.800000

Criterion: C6; Sub-criterion: C6-1 C6-2 C6-3

PCM of Sub-criterion w.r.t. Criterion:

1.000000   0.600000   1.500000

1.666667   1.000000   2.500000

0.666667   0.400000   1.000000

The principal eigenvalue is: 3.000000

The Consistency Ratio is: 0.000000

The PCM HAS acceptable consistency.

Local weights of the sub-criterion (eigenvector):

0.300000   0.500000   0.200000

Criterion: C7;   Sub-criterion: C7-1   C7-2   C7-3

PCM of Sub-criterion w.r.t. Criterion:

1.000000   1.333333   1.333333

0.750000   1.000000   1.000000

0.750000   1.000000   1.000000

The principal eigenvalue is: 3.000000

The Consistency Ratio is: 0.000000

The PCM HAS acceptable consistency.

Local weights of the sub-criterion (eigenvector):

0.400000   0.300000   0.300000

Alternatives: A2   A3   A4   A5

Leaf criteria: C2   C1-1   C1-2   C1-3   C3-1   C3-2   C3-3   C4-1   C4-2   C4-3   C4-4   C5-1   C5-2   C6-1   C6-2   C6-3   C7-1   C7-2   C7-3

PCM for Alternatives w.r.t. leaf criterion C2

1.000000   0.666667   0.500000   0.500000

1.500000   1.000000   0.750000   0.750000

2.000000   1.333333   1.000000   1.000000

2.000000   1.333333   1.000000   1.000000

The principal eigenvalue is: 4.000000

The Consistency Ratio is: 0.000000

The PCM HAS acceptable consistency.

Local weights of the alternatives (eigenvector):

0.153846   0.230769   0.307692   0.307692

PCM for Alternatives w.r.t. leaf criterion C1-1

1.000000	0.666667	1.250000	1.666667
1.500000	1.000000	1.875000	2.500000
0.800000	0.533333	1.000000	1.333333
0.600000	0.400000	0.750000	1.000000

The principal eigenvalue is: 4.000000

The Consistency Ratio is: 0.000000

The PCM HAS acceptable consistency.

Local weights of the alternatives (eigenvector):

0.256410 0.384615 0.205128 0.153846

PCM for Alternatives w.r.t. leaf criterion C1-2

1.000000	1.500000	2.000000	2.500000
0.666667	1.000000	1.333333	1.666667
0.500000	0.750000	1.000000	1.250000
0.400000	0.600000	0.800000	1.000000

The principal eigenvalue is: 4.000000

The Consistency Ratio is: 0.000000

The PCM HAS acceptable consistency.

Local weights of the alternatives (eigenvector):

0.389610   0.259740   0.194805   0.155844

PCM for Alternatives w.r.t. leaf criterion C1-3

1.000000	0.333333	1.333333	0.833333
3.000000	1.000000	4.000000	2.500000
0.750000	0.250000	1.000000	0.625000
1.200000	0.400000	1.600000	1.000000

The principal eigenvalue is: 4.000000

The Consistency Ratio is: 0.000000

The PCM HAS acceptable consistency.

Local weights of the alternatives (eigenvector):

0.168067   0.504202   0.126050   0.201681

PCM for Alternatives w.r.t. leaf criterion C3-1

1.000000	0.166667	0.125000	0.250000
6.000000	1.000000	0.750000	1.500000
8.000000	1.333333	1.000000	2.000000

4.000000 0.666667 0.500000 1.000000

The principal eigenvalue is: 4.000000

The Consistency Ratio is: 0.000000

The PCM HAS acceptable consistency.

Local weights of the alternatives (eigenvector):

0.052632 0.315789 0.421053 0.210526

PCM for Alternatives w.r.t. leaf criterion C3-2

1.000000 2.000000 16.000000 1.000000

0.500000 1.000000 8.000000 0.500000

0.062500 0.125000 1.000000 0.062500

1.000000 2.000000 16.000000 1.000000

The principal eigenvalue is: 4.000000

The Consistency Ratio is: 0.000000

The PCM HAS acceptable consistency.

Local weights of the alternatives (eigenvector):

0.390244 0.195122 0.024390 0.390244

PCM for Alternatives w.r.t. leaf criterion C3-3

1.000000	0.500000	0.066667	0.200000
2.000000	1.000000	0.133333	0.400000
15.000000	7.500000	1.000000	3.000000
5.000000	2.500000	0.333333	1.000000

The principal eigenvalue is: 4.000000

The Consistency Ratio is: 0.000000

The PCM HAS acceptable consistency.

Local weights of the alternatives (eigenvector):

0.043478   0.086957   0.652174   0.217391

PCM for Alternatives w.r.t. leaf criterion C4-1

1.000000	1.000000	0.125000	1.250000
1.000000	1.000000	0.125000	1.250000
8.000000	8.000000	1.000000	10.000000
0.800000	0.800000	0.100000	1.000000

The principal eigenvalue is: 4.000000

The Consistency Ratio is: 0.000000

The PCM HAS acceptable consistency.

Local weights of the alternatives (eigenvector):

0.092593 0.092593 0.740741 0.074074

PCM for Alternatives w.r.t. leaf criterion C4-2

1.000000	2.000000	8.000000	0.800000
0.500000	1.000000	4.000000	0.400000
0.125000	0.250000	1.000000	0.100000
1.250000	2.500000	10.000000	1.000000

The principal eigenvalue is: 4.000000

The Consistency Ratio is: 0.000000

The PCM HAS acceptable consistency.

Local weights of the alternatives (eigenvector):

0.347826 0.173913 0.043478 0.434783

PCM for Alternatives w.r.t. leaf criterion C4-3

1.000000	0.400000	0.500000	4.000000
2.500000	1.000000	1.250000	10.000000
2.000000	0.800000	1.000000	8.000000

0.250000 0.100000 0.125000 1.000000

The principal eigenvalue is: 4.000000

The Consistency Ratio is: 0.000000

The PCM HAS acceptable consistency.

Local weights of the alternatives (eigenvector):

0.173913 0.434783 0.347826 0.043478

PCM for Alternatives w.r.t. leaf criterion C4-4

1.000000 0.750000 0.062500 0.500000

1.333333 1.000000 0.083333 0.666667

16.000000 12.000000 1.000000 8.000000

2.000000 1.500000 0.125000 1.000000

The principal eigenvalue is: 4.000000

The Consistency Ratio is: 0.000000

The PCM HAS acceptable consistency.

Local weights of the alternatives (eigenvector):

0.049180 0.065574 0.786885 0.098361

PCM for Alternatives w.r.t. leaf criterion C5-1

1.000000	0.600000	0.400000	1.600000
1.666667	1.000000	0.666667	2.666667
2.500000	1.500000	1.000000	4.000000
0.625000	0.375000	0.250000	1.000000

The principal eigenvalue is: 4.000000

The Consistency Ratio is: 0.000000

The PCM HAS acceptable consistency.

Local weights of the alternatives (eigenvector):

0.172662 0.287770 0.431655 0.107914

PCM for Alternatives w.r.t. leaf criterion C5-2

1.000000	2.500000	20.000000	1.250000
0.400000	1.000000	8.000000	0.500000
0.050000	0.125000	1.000000	0.062500
0.800000	2.000000	16.000000	1.000000

The principal eigenvalue is: 4.000000

The Consistency Ratio is: 0.000000

The PCM HAS acceptable consistency.

Local weights of the alternatives (eigenvector):

0.444444 0.177778 0.022222 0.355556

PCM for Alternatives w.r.t. leaf criterion C6-1

1.000000	6.000000	8.000000	2.000000
0.166667	1.000000	1.333333	0.333333
0.125000	0.750000	1.000000	0.250000
0.500000	3.000000	4.000000	1.000000

The principal eigenvalue is: 4.000000

The Consistency Ratio is: 0.000000

The PCM HAS acceptable consistency.

Local weights of the alternatives (eigenvector):

0.558140 0.093023 0.069767 0.279070

PCM for Alternatives w.r.t. leaf criterion C6-2

1.000000	1.000000	0.062500	0.125000
1.000000	1.000000	0.062500	0.125000

16.000000	16.000000	1.000000	2.000000
8.000000	8.000000	0.500000	1.000000

The principal eigenvalue is: 4.000000

The Consistency Ratio is: 0.000000

The PCM HAS acceptable consistency.

Local weights of the alternatives (eigenvector):

0.038462 0.038462 0.615385 0.307692

PCM for Alternatives w.r.t. leaf criterion C6-3

1.000000	2.000000	1.000000	2.000000
0.500000	1.000000	0.500000	1.000000
1.000000	2.000000	1.000000	2.000000
0.500000	1.000000	0.500000	1.000000

The principal eigenvalue is: 4.000000

The Consistency Ratio is: 0.000000

The PCM HAS acceptable consistency.

Local weights of the alternatives (eigenvector):

0.333333 0.166667 0.333333 0.166667

PCM for Alternatives w.r.t. leaf criterion C7-1

1.000000	2.500000	10.000000	1.250000
0.400000	1.000000	4.000000	0.500000
0.100000	0.250000	1.000000	0.125000
0.800000	2.000000	8.000000	1.000000

The principal eigenvalue is: 4.000000

The Consistency Ratio is: 0.000000

The PCM HAS acceptable consistency.

Local weights of the alternatives (eigenvector):

0.434783 0.173913 0.043478 0.347826

PCM for Alternatives w.r.t. leaf criterion C7-2

1.000000	0.250000	0.500000	1.000000
4.000000	1.000000	2.000000	4.000000
2.000000	0.500000	1.000000	2.000000
1.000000	0.250000	0.500000	1.000000

The principal eigenvalue is: 4.000000

The Consistency Ratio is: 0.000000

The PCM HAS acceptable consistency.

Local weights of the alternatives (eigenvector):

0.125000 0.500000 0.250000 0.125000

PCM for Alternatives w.r.t. leaf criterion C7-3

1.000000	0.500000	0.125000	1.000000
2.000000	1.000000	0.250000	2.000000
8.000000	4.000000	1.000000	8.000000
1.000000	0.500000	0.125000	1.000000

The principal eigenvalue is: 4.000000

The Consistency Ratio is: 0.000000

The PCM HAS acceptable consistency.

Local weights of the alternatives (eigenvector):

0.083333 0.166667 0.666667 0.083333

Global weights of the leaf criterion:

0.146789 0.088073 0.088073 0.044037 0.049541 0.049541 0.066055 0.041284
0.027523 0.027523 0.041284 0.022018 0.088074 0.044037 0.073395 0.029358
0.029358 0.022018 0.022018

Alternatives' global weights:

0.229391 0.220757 0.315869 0.233982

The hierarchy structure:

7 T TOP C1 1 C2 0 C3 1 C4 1 C5 1 C6 1 C7 1

3 C1 T C1-1 0 C1-2 0 C1-3 0

3 C3 T C3-1 0 C3-2 0 C3-3 0

4 C4 T C4-1 0 C4-2 0 C4-3 0 C4-4 0

2 C5 T C5-1 0 C5-2 0

3 C6 T C6-1 0 C6-2 0 C6-3 0

3 C7 T C7-1 0 C7-2 0 C7-3 0

The number of alternatives:4

Criterion: T; Sub-criterion: C1 C2 C3 C4 C5 C6 C7

PCM of Sub-criterion w.r.t. Criterion:

1.000000	1.500000	1.333333	1.600000	2.000000	1.500000	3.000000
0.666667	1.000000	0.888889	1.066667	1.333333	1.000000	2.000000
0.750000	1.125000	1.000000	1.200000	1.500000	1.125000	2.250000
0.625000	0.937500	0.833333	1.000000	1.250000	0.937500	1.875000
0.500000	0.750000	0.666667	0.800000	1.000000	0.750000	1.500000
0.666667	1.000000	0.888889	1.066667	1.333333	1.000000	2.000000
0.333333	0.500000	0.444444	0.533333	0.666667	0.500000	1.000000

The principal eigenvalue is: 7.000000

The Consistency Ratio is: 0.000000

The PCM HAS acceptable consistency.

Local weights of the sub-criterion (eigenvector):

0.220183 0.146789 0.165138 0.137615 0.110092 0.146789 0.073394

Criterion: C1; Sub-criterion: C1-1 C1-2 C1-3

PCM of Sub-criterion w.r.t. Criterion:

1.000000 1.000000 2.000000

1.000000 1.000000 2.000000

0.500000 0.500000 1.000000

The principal eigenvalue is: 3.000000

The Consistency Ratio is: 0.000000

The PCM HAS acceptable consistency.

Local weights of the sub-criterion (eigenvector):

0.400000 0.400000 0.200000

Criterion: C3;   Sub-criterion: C3-1   C3-2   C3-3

PCM of Sub-criterion w.r.t. Criterion:

1.000000	1.000000	0.750000
1.000000	1.000000	0.750000
1.333333	1.333333	1.000000

The principal eigenvalue is: 3.000000

The Consistency Ratio is: 0.000000

The PCM HAS acceptable consistency.

Local weights of the sub-criterion (eigenvector):

0.300000   0.300000   0.400000

Criterion: C4;   Sub-criterion: C4-1   C4-2   C4-3   C4-4

PCM of Sub-criterion w.r.t. Criterion:

1.000000	1.500000	1.500000	1.000000
0.666667	1.000000	1.000000	0.666667
0.666667	1.000000	1.000000	0.666667
1.000000	1.500000	1.500000	1.000000

The principal eigenvalue is: 4.000000

The Consistency Ratio is: 0.000000

The PCM HAS acceptable consistency.

Local weights of the sub-criterion (eigenvector):

0.300000 0.200000 0.200000 0.300000

Criterion: C5; Sub-criterion: C5-1 C5-2

PCM of Sub-criterion w.r.t. Criterion:

1.000000 0.250000

4.000000 1.000000

The principal eigenvalue is: 2.000000

The Consistency Ratio is: 0.000000

The PCM HAS acceptable consistency.

Local weights of the sub-criterion (eigenvector):

0.200000 0.800000

Criterion: C6; Sub-criterion: C6-1 C6-2 C6-3

PCM of Sub-criterion w.r.t. Criterion:

1.000000 0.600000 1.500000

1.666667 1.000000 2.500000

0.666667 0.400000 1.000000

The principal eigenvalue is: 3.000000

The Consistency Ratio is: 0.000000

The PCM HAS acceptable consistency.

Local weights of the sub-criterion (eigenvector):

0.300000 0.500000 0.200000

Criterion: C7; Sub-criterion: C7-1 C7-2 C7-3

PCM of Sub-criterion w.r.t. Criterion:

1.000000 1.333333 1.333333

0.750000 1.000000 1.000000

0.750000 1.000000 1.000000

The principal eigenvalue is: 3.000000

The Consistency Ratio is: 0.000000

The PCM HAS acceptable consistency.

Local weights of the sub-criterion (eigenvector):

0.400000 0.300000 0.300000

Alternatives: A1 A3 A4 A5

Leaf criteria: C2 C1-1 C1-2 C1-3 C3-1 C3-2 C3-3 C4-1 C4-2 C4-3 C4-4 C5-1 C5-2 C6-1 C6-2 C6-3 C7-1 C7-2 C7-3

PCM for Alternatives w.r.t. leaf criterion C2

1.000000	0.333333	0.250000	0.250000
3.000000	1.000000	0.750000	0.750000
4.000000	1.333333	1.000000	1.000000
4.000000	1.333333	1.000000	1.000000

The principal eigenvalue is: 4.000000

The Consistency Ratio is: 0.000000

The PCM HAS acceptable consistency.

Local weights of the alternatives (eigenvector):

0.083333 0.250000 0.333333 0.333333

PCM for Alternatives w.r.t. leaf criterion C1-1

1.000000	0.666667	1.250000	1.666667
1.500000	1.000000	1.875000	2.500000
0.800000	0.533333	1.000000	1.333333
0.600000	0.400000	0.750000	1.000000

The principal eigenvalue is: 4.000000

The Consistency Ratio is: 0.000000

The PCM HAS acceptable consistency.

Local weights of the alternatives (eigenvector):

0.256410 0.384615 0.205128 0.153846

PCM for Alternatives w.r.t. leaf criterion C1-2

1.000000	3.000000	4.000000	5.000000
0.333333	1.000000	1.333333	1.666667
0.250000	0.750000	1.000000	1.250000
0.200000	0.600000	0.800000	1.000000

The principal eigenvalue is: 4.000000

The Consistency Ratio is: 0.000000

The PCM HAS acceptable consistency.

Local weights of the alternatives (eigenvector):

0.560748 0.186916 0.140187 0.112150

PCM for Alternatives w.r.t. leaf criterion C1-3

1.000000	0.500000	2.000000	1.250000
2.000000	1.000000	4.000000	2.500000
0.500000	0.250000	1.000000	0.625000
0.800000	0.400000	1.600000	1.000000

The principal eigenvalue is: 4.000000

The Consistency Ratio is: 0.000000

The PCM HAS acceptable consistency.

Local weights of the alternatives (eigenvector):

0.232558 0.465116 0.116279 0.186047

PCM for Alternatives w.r.t. leaf criterion C3-1

1.000000	0.333333	0.250000	0.500000
3.000000	1.000000	0.750000	1.500000
4.000000	1.333333	1.000000	2.000000

2.000000 0.666667 0.500000 1.000000

The principal eigenvalue is: 4.000000

The Consistency Ratio is: 0.000000

The PCM HAS acceptable consistency.

Local weights of the alternatives (eigenvector):

0.100000 0.300000 0.400000 0.200000

PCM for Alternatives w.r.t. leaf criterion C3-2

1.000000	0.500000	4.000000	0.250000
2.000000	1.000000	8.000000	0.500000
0.250000	0.125000	1.000000	0.062500
4.000000	2.000000	16.000000	1.000000

The principal eigenvalue is: 4.000000

The Consistency Ratio is: 0.000000

The PCM HAS acceptable consistency.

Local weights of the alternatives (eigenvector):

0.137931 0.275862 0.034483 0.551724

PCM for Alternatives w.r.t. leaf criterion C3-3

1.000000	2.500000	0.333333	1.000000
0.400000	1.000000	0.133333	0.400000
3.000000	7.500000	1.000000	3.000000
1.000000	2.500000	0.333333	1.000000

The principal eigenvalue is: 4.000000

The Consistency Ratio is: 0.000000

The PCM HAS acceptable consistency.

Local weights of the alternatives (eigenvector):

0.185185 0.074074 0.555556 0.185185

PCM for Alternatives w.r.t. leaf criterion C4-1

1.000000	4.000000	0.500000	5.000000
0.250000	1.000000	0.125000	1.250000
2.000000	8.000000	1.000000	10.000000
0.200000	0.800000	0.100000	1.000000

The principal eigenvalue is: 4.000000

The Consistency Ratio is: 0.000000

The PCM HAS acceptable consistency.

Local weights of the alternatives (eigenvector):

0.289855 0.072464 0.579710 0.057971

PCM for Alternatives w.r.t. leaf criterion C4-2

1.000000	0.500000	2.000000	0.200000
2.000000	1.000000	4.000000	0.400000
0.500000	0.250000	1.000000	0.100000
5.000000	2.500000	10.000000	1.000000

The principal eigenvalue is: 4.000000

The Consistency Ratio is: 0.000000

The PCM HAS acceptable consistency.

Local weights of the alternatives (eigenvector):

0.117647 0.235294 0.058824 0.588235

PCM for Alternatives w.r.t. leaf criterion C4-3

1.000000	0.200000	0.250000	2.000000
5.000000	1.000000	1.250000	10.000000
4.000000	0.800000	1.000000	8.000000

0.500000 0.100000 0.125000 1.000000

The principal eigenvalue is: 4.000000

The Consistency Ratio is: 0.000000

The PCM HAS acceptable consistency.

Local weights of the alternatives (eigenvector):

0.095238 0.476190 0.380952 0.047619

PCM for Alternatives w.r.t. leaf criterion C4-4

1.000000	6.000000	0.500000	4.000000
0.166667	1.000000	0.083333	0.666667
2.000000	12.000000	1.000000	8.000000
0.250000	1.500000	0.125000	1.000000

The principal eigenvalue is: 4.000000

The Consistency Ratio is: 0.000000

The PCM HAS acceptable consistency.

Local weights of the alternatives (eigenvector):

0.292683 0.048780 0.585366 0.073171

PCM for Alternatives w.r.t. leaf criterion C5-1

1.000000	3.000000	2.000000	8.000000
0.333333	1.000000	0.666667	2.666667
0.500000	1.500000	1.000000	4.000000
0.125000	0.375000	0.250000	1.000000

The principal eigenvalue is: 4.000000

The Consistency Ratio is: 0.000000

The PCM HAS acceptable consistency.

Local weights of the alternatives (eigenvector):

0.510638 0.170213 0.255319 0.063830

PCM for Alternatives w.r.t. leaf criterion C5-2

1.000000	0.500000	4.000000	0.250000
2.000000	1.000000	8.000000	0.500000
0.250000	0.125000	1.000000	0.062500
4.000000	2.000000	16.000000	1.000000

The principal eigenvalue is: 4.000000

The Consistency Ratio is: 0.000000

The PCM HAS acceptable consistency.

Local weights of the alternatives (eigenvector):

0.137931 0.275862 0.034483 0.551724

PCM for Alternatives w.r.t. leaf criterion C6-1

1.000000	3.000000	4.000000	1.000000
0.333333	1.000000	1.333333	0.333333
0.250000	0.750000	1.000000	0.250000
1.000000	3.000000	4.000000	1.000000

The principal eigenvalue is: 4.000000

The Consistency Ratio is: 0.000000

The PCM HAS acceptable consistency.

Local weights of the alternatives (eigenvector):

0.387097 0.129032 0.096774 0.387097

PCM for Alternatives w.r.t. leaf criterion C6-2

1.000000	4.000000	0.250000	0.500000
0.250000	1.000000	0.062500	0.125000

4.000000 16.000000 1.000000 2.000000

2.000000 8.000000 0.500000 1.000000

The principal eigenvalue is: 4.000000

The Consistency Ratio is: 0.000000

The PCM HAS acceptable consistency.

Local weights of the alternatives (eigenvector):

0.137931 0.034483 0.551724 0.275862

PCM for Alternatives w.r.t. leaf criterion C6-3

1.000000 0.500000 0.250000 0.500000

2.000000 1.000000 0.500000 1.000000

4.000000 2.000000 1.000000 2.000000

2.000000 1.000000 0.500000 1.000000

The principal eigenvalue is: 4.000000

The Consistency Ratio is: 0.000000

The PCM HAS acceptable consistency.

Local weights of the alternatives (eigenvector):

0.111111 0.222222 0.444444 0.222222

PCM for Alternatives w.r.t. leaf criterion C7-1

1.000000	0.500000	2.000000	0.250000
2.000000	1.000000	4.000000	0.500000
0.500000	0.250000	1.000000	0.125000
4.000000	2.000000	8.000000	1.000000

The principal eigenvalue is: 4.000000

The Consistency Ratio is: 0.000000

The PCM HAS acceptable consistency.

Local weights of the alternatives (eigenvector):

0.133333 0.266667 0.066667 0.533333

PCM for Alternatives w.r.t. leaf criterion C7-2

1.000000	0.500000	1.000000	2.000000
2.000000	1.000000	2.000000	4.000000
1.000000	0.500000	1.000000	2.000000
0.500000	0.250000	0.500000	1.000000

The principal eigenvalue is: 4.000000

The Consistency Ratio is: 0.000000

The PCM HAS acceptable consistency.

Local weights of the alternatives (eigenvector):

0.222222 0.444444 0.222222 0.111111

PCM for Alternatives w.r.t. leaf criterion C7-3

1.000000	2.000000	0.500000	4.000000
0.500000	1.000000	0.250000	2.000000
2.000000	4.000000	1.000000	8.000000
0.250000	0.500000	0.125000	1.000000

The principal eigenvalue is: 4.000000

The Consistency Ratio is: 0.000000

The PCM HAS acceptable consistency.

Local weights of the alternatives (eigenvector):

0.266667 0.133333 0.533333 0.066667

Global weights of the leaf criterion:

0.146789 0.088073 0.088073 0.044037 0.049541 0.049541 0.066055 0.041284
0.027523 0.027523 0.041284 0.022018 0.088074 0.044037 0.073395 0.029358
0.029358 0.022018 0.022018

Alternatives' global weights:

0.216874 0.228857 0.287943 0.266325

The hierarchy structure:

7 T TOP C1 1 C2 0 C3 1 C4 1 C5 1 C6 1 C7 1

3 C1 T C1-1 0 C1-2 0 C1-3 0

3 C3 T C3-1 0 C3-2 0 C3-3 0

4 C4 T C4-1 0 C4-2 0 C4-3 0 C4-4 0

2 C5 T C5-1 0 C5-2 0

3 C6 T C6-1 0 C6-2 0 C6-3 0

3 C7 T C7-1 0 C7-2 0 C7-3 0

The number of alternatives:4

Criterion: T; Sub-criterion: C1 C2 C3 C4 C5 C6 C7

PCM of Sub-criterion w.r.t. Criterion:

1.000000	1.500000	1.333333	1.600000	2.000000	1.500000	3.000000
0.666667	1.000000	0.888889	1.066667	1.333333	1.000000	2.000000
0.750000	1.125000	1.000000	1.200000	1.500000	1.125000	2.250000
0.625000	0.937500	0.833333	1.000000	1.250000	0.937500	1.875000
0.500000	0.750000	0.666667	0.800000	1.000000	0.750000	1.500000
0.666667	1.000000	0.888889	1.066667	1.333333	1.000000	2.000000
0.333333	0.500000	0.444444	0.533333	0.666667	0.500000	1.000000

The principal eigenvalue is: 7.000000

The Consistency Ratio is: 0.000000

The PCM HAS acceptable consistency.

Local weights of the sub-criterion (eigenvector):

0.220183   0.146789   0.165138   0.137615   0.110092   0.146789   0.073394

Criterion: C1;   Sub-criterion: C1-1   C1-2   C1-3

PCM of Sub-criterion w.r.t. Criterion:

1.000000	1.000000	2.000000
1.000000	1.000000	2.000000
0.500000	0.500000	1.000000

The principal eigenvalue is: 3.000000

The Consistency Ratio is: 0.000000

The PCM HAS acceptable consistency.

Local weights of the sub-criterion (eigenvector):

0.400000   0.400000   0.200000

Criterion: C3;   Sub-criterion: C3-1   C3-2   C3-3

PCM of Sub-criterion w.r.t. Criterion:

1.000000	1.000000	0.750000
1.000000	1.000000	0.750000
1.333333	1.333333	1.000000

The principal eigenvalue is: 3.000000

The Consistency Ratio is: 0.000000

The PCM HAS acceptable consistency.

Local weights of the sub-criterion (eigenvector):

0.300000   0.300000   0.400000

Criterion: C4;   Sub-criterion: C4-1   C4-2   C4-3   C4-4

PCM of Sub-criterion w.r.t. Criterion:

1.000000	1.500000	1.500000	1.000000
0.666667	1.000000	1.000000	0.666667
0.666667	1.000000	1.000000	0.666667
1.000000	1.500000	1.500000	1.000000

The principal eigenvalue is: 4.000000

The Consistency Ratio is: 0.000000

The PCM HAS acceptable consistency.

Local weights of the sub-criterion (eigenvector):

0.300000   0.200000   0.200000   0.300000

Criterion: C5;   Sub-criterion: C5-1   C5-2

PCM of Sub-criterion w.r.t. Criterion:

1.000000   0.250000

4.000000   1.000000

The principal eigenvalue is: 2.000000

The Consistency Ratio is: 0.000000

The PCM HAS acceptable consistency.

Local weights of the sub-criterion (eigenvector):

0.200000   0.800000

Criterion: C6;   Sub-criterion: C6-1   C6-2   C6-3

PCM of Sub-criterion w.r.t. Criterion:

1.000000 0.600000 1.500000

1.666667 1.000000 2.500000

0.666667 0.400000 1.000000

The principal eigenvalue is: 3.000000

The Consistency Ratio is: 0.000000

The PCM HAS acceptable consistency.

Local weights of the sub-criterion (eigenvector):

0.300000 0.500000 0.200000

Criterion: C7; Sub-criterion: C7-1 C7-2 C7-3

PCM of Sub-criterion w.r.t. Criterion:

1.000000 1.333333 1.333333

0.750000 1.000000 1.000000

0.750000 1.000000 1.000000

The principal eigenvalue is: 3.000000

The Consistency Ratio is: 0.000000

The PCM HAS acceptable consistency.

Local weights of the sub-criterion (eigenvector):

0.400000   0.300000   0.300000

Alternatives: A1   A2   A4   A5

Leaf criteria: C2   C1-1   C1-2   C1-3   C3-1   C3-2   C3-3   C4-1   C4-2   C4-3   C4-4   C5-1   C5-2   C6-1   C6-2   C6-3   C7-1   C7-2   C7-3

PCM for Alternatives w.r.t. leaf criterion C2

1.000000	0.500000	0.250000	0.250000
2.000000	1.000000	0.500000	0.500000
4.000000	2.000000	1.000000	1.000000
4.000000	2.000000	1.000000	1.000000

The principal eigenvalue is: 4.000000

The Consistency Ratio is: 0.000000

The PCM HAS acceptable consistency.

Local weights of the alternatives (eigenvector):

0.090909   0.181818   0.363636   0.363636

PCM for Alternatives w.r.t. leaf criterion C1-1

1.000000	1.000000	1.250000	1.666667
1.000000	1.000000	1.250000	1.666667
0.800000	0.800000	1.000000	1.333333
0.600000	0.600000	0.750000	1.000000

The principal eigenvalue is: 4.000000

The Consistency Ratio is: 0.000000

The PCM HAS acceptable consistency.

Local weights of the alternatives (eigenvector):

0.294118 0.294118 0.235294 0.176471

PCM for Alternatives w.r.t. leaf criterion C1-2

1.000000	2.000000	4.000000	5.000000
0.500000	1.000000	2.000000	2.500000
0.250000	0.500000	1.000000	1.250000
0.200000	0.400000	0.800000	1.000000

The principal eigenvalue is: 4.000000

The Consistency Ratio is: 0.000000

The PCM HAS acceptable consistency.

Local weights of the alternatives (eigenvector):

0.512821 0.256410 0.128205 0.102564

PCM for Alternatives w.r.t. leaf criterion C1-3

1.000000	1.500000	2.000000	1.250000
0.666667	1.000000	1.333333	0.833333
0.500000	0.750000	1.000000	0.625000
0.800000	1.200000	1.600000	1.000000

The principal eigenvalue is: 4.000000

The Consistency Ratio is: 0.000000

The PCM HAS acceptable consistency.

Local weights of the alternatives (eigenvector):

0.337079 0.224719 0.168539 0.269663

PCM for Alternatives w.r.t. leaf criterion C3-1

1.000000	2.000000	0.250000	0.500000
0.500000	1.000000	0.125000	0.250000
4.000000	8.000000	1.000000	2.000000

2.000000 4.000000 0.500000 1.000000

The principal eigenvalue is: 4.000000

The Consistency Ratio is: 0.000000

The PCM HAS acceptable consistency.

Local weights of the alternatives (eigenvector):

0.133333 0.066667 0.533333 0.266667

PCM for Alternatives w.r.t. leaf criterion C3-2

1.000000	0.250000	4.000000	0.250000
4.000000	1.000000	16.000000	1.000000
0.250000	0.062500	1.000000	0.062500
4.000000	1.000000	16.000000	1.000000

The principal eigenvalue is: 4.000000

The Consistency Ratio is: 0.000000

The PCM HAS acceptable consistency.

Local weights of the alternatives (eigenvector):

0.108108 0.432432 0.027027 0.432432

PCM for Alternatives w.r.t. leaf criterion C3-3

1.000000	5.000000	0.333333	1.000000
0.200000	1.000000	0.066667	0.200000
3.000000	15.000000	1.000000	3.000000
1.000000	5.000000	0.333333	1.000000

The principal eigenvalue is: 4.000000

The Consistency Ratio is: 0.000000

The PCM HAS acceptable consistency.

Local weights of the alternatives (eigenvector):

0.192308 0.038462 0.576923 0.192308

PCM for Alternatives w.r.t. leaf criterion C4-1

1.000000	4.000000	0.500000	5.000000
0.250000	1.000000	0.125000	1.250000
2.000000	8.000000	1.000000	10.000000
0.200000	0.800000	0.100000	1.000000

The principal eigenvalue is: 4.000000

The Consistency Ratio is: 0.000000

The PCM HAS acceptable consistency.

Local weights of the alternatives (eigenvector):

0.289855 0.072464 0.579710 0.057971

PCM for Alternatives w.r.t. leaf criterion C4-2

1.000000	0.250000	2.000000	0.200000
4.000000	1.000000	8.000000	0.800000
0.500000	0.125000	1.000000	0.100000
5.000000	1.250000	10.000000	1.000000

The principal eigenvalue is: 4.000000

The Consistency Ratio is: 0.000000

The PCM HAS acceptable consistency.

Local weights of the alternatives (eigenvector):

0.095238 0.380952 0.047619 0.476190

PCM for Alternatives w.r.t. leaf criterion C4-3

1.000000	0.500000	0.250000	2.000000
2.000000	1.000000	0.500000	4.000000
4.000000	2.000000	1.000000	8.000000

0.500000 0.250000 0.125000 1.000000

The principal eigenvalue is: 4.000000

The Consistency Ratio is: 0.000000

The PCM HAS acceptable consistency.

Local weights of the alternatives (eigenvector):

0.133333 0.266667 0.533333 0.066667

PCM for Alternatives w.r.t. leaf criterion C4-4

1.000000 8.000000 0.500000 4.000000

0.125000 1.000000 0.062500 0.500000

2.000000 16.000000 1.000000 8.000000

0.250000 2.000000 0.125000 1.000000

The principal eigenvalue is: 4.000000

The Consistency Ratio is: 0.000000

The PCM HAS acceptable consistency.

Local weights of the alternatives (eigenvector):

0.296296 0.037037 0.592593 0.074074

PCM for Alternatives w.r.t. leaf criterion C5-1

1.000000	5.000000	2.000000	8.000000
0.200000	1.000000	0.400000	1.600000
0.500000	2.500000	1.000000	4.000000
0.125000	0.625000	0.250000	1.000000

The principal eigenvalue is: 4.000000

The Consistency Ratio is: 0.000000

The PCM HAS acceptable consistency.

Local weights of the alternatives (eigenvector):

0.547945 0.109589 0.273973 0.068493

PCM for Alternatives w.r.t. leaf criterion C5-2

1.000000	0.200000	4.000000	0.250000
5.000000	1.000000	20.000000	1.250000
0.250000	0.050000	1.000000	0.062500
4.000000	0.800000	16.000000	1.000000

The principal eigenvalue is: 4.000000

The Consistency Ratio is: 0.000000

The PCM HAS acceptable consistency.

Local weights of the alternatives (eigenvector):

0.097561 0.487805 0.024390 0.390244

PCM for Alternatives w.r.t. leaf criterion C6-1

1.000000	0.500000	4.000000	1.000000
2.000000	1.000000	8.000000	2.000000
0.250000	0.125000	1.000000	0.250000
1.000000	0.500000	4.000000	1.000000

The principal eigenvalue is: 4.000000

The Consistency Ratio is: 0.000000

The PCM HAS acceptable consistency.

Local weights of the alternatives (eigenvector):

0.235294 0.470588 0.058824 0.235294

PCM for Alternatives w.r.t. leaf criterion C6-2

1.000000	4.000000	0.250000	0.500000
0.250000	1.000000	0.062500	0.125000

4.000000 16.000000 1.000000 2.000000

2.000000 8.000000 0.500000 1.000000

The principal eigenvalue is: 4.000000

The Consistency Ratio is: 0.000000

The PCM HAS acceptable consistency.

Local weights of the alternatives (eigenvector):

0.137931 0.034483 0.551724 0.275862

PCM for Alternatives w.r.t. leaf criterion C6-3

1.000000 0.250000 0.250000 0.500000

4.000000 1.000000 1.000000 2.000000

4.000000 1.000000 1.000000 2.000000

2.000000 0.500000 0.500000 1.000000

The principal eigenvalue is: 4.000000

The Consistency Ratio is: 0.000000

The PCM HAS acceptable consistency.

Local weights of the alternatives (eigenvector):

0.090909 0.363636 0.363636 0.181818

PCM for Alternatives w.r.t. leaf criterion C7-1

1.000000	0.200000	2.000000	0.250000
5.000000	1.000000	10.000000	1.250000
0.500000	0.100000	1.000000	0.125000
4.000000	0.800000	8.000000	1.000000

The principal eigenvalue is: 4.000000

The Consistency Ratio is: 0.000000

The PCM HAS acceptable consistency.

Local weights of the alternatives (eigenvector):

0.095238 0.476190 0.047619 0.380952

PCM for Alternatives w.r.t. leaf criterion C7-2

1.000000	2.000000	1.000000	2.000000
0.500000	1.000000	0.500000	1.000000
1.000000	2.000000	1.000000	2.000000
0.500000	1.000000	0.500000	1.000000

The principal eigenvalue is: 4.000000

The Consistency Ratio is: 0.000000

The PCM HAS acceptable consistency.

Local weights of the alternatives (eigenvector):

0.333333   0.166667   0.333333   0.166667

PCM for Alternatives w.r.t. leaf criterion C7-3

1.000000	4.000000	0.500000	4.000000
0.250000	1.000000	0.125000	1.000000
2.000000	8.000000	1.000000	8.000000
0.250000	1.000000	0.125000	1.000000

The principal eigenvalue is: 4.000000

The Consistency Ratio is: 0.000000

The PCM HAS acceptable consistency.

Local weights of the alternatives (eigenvector):

0.285714   0.071429   0.571429   0.071429

Global weights of the leaf criterion:

0.146789   0.088073   0.088073   0.044037   0.049541   0.049541   0.066055   0.041284
0.027523   0.027523   0.041284   0.022018   0.088074   0.044037   0.073395   0.029358
0.029358   0.022018   0.022018

Alternatives' global weights:

0.214649 0.233211 0.306330 0.245809

The hierarchy structure:

7 T TOP C1 1 C2 0 C3 1 C4 1 C5 1 C6 1 C7 1

3 C1 T C1-1 0 C1-2 0 C1-3 0

3 C3 T C3-1 0 C3-2 0 C3-3 0

4 C4 T C4-1 0 C4-2 0 C4-3 0 C4-4 0

2 C5 T C5-1 0 C5-2 0

3 C6 T C6-1 0 C6-2 0 C6-3 0

3 C7 T C7-1 0 C7-2 0 C7-3 0

The number of alternatives:4

Criterion: T; Sub-criterion: C1 C2 C3 C4 C5 C6 C7

PCM of Sub-criterion w.r.t. Criterion:

1.000000	1.500000	1.333333	1.600000	2.000000	1.500000	3.000000
0.666667	1.000000	0.888889	1.066667	1.333333	1.000000	2.000000
0.750000	1.125000	1.000000	1.200000	1.500000	1.125000	2.250000
0.625000	0.937500	0.833333	1.000000	1.250000	0.937500	1.875000
0.500000	0.750000	0.666667	0.800000	1.000000	0.750000	1.500000
0.666667	1.000000	0.888889	1.066667	1.333333	1.000000	2.000000
0.333333	0.500000	0.444444	0.533333	0.666667	0.500000	1.000000

The principal eigenvalue is: 7.000000

The Consistency Ratio is: 0.000000

The PCM HAS acceptable consistency.

Local weights of the sub-criterion (eigenvector):

0.220183 0.146789 0.165138 0.137615 0.110092 0.146789 0.073394

Criterion: C1; Sub-criterion: C1-1 C1-2 C1-3

PCM of Sub-criterion w.r.t. Criterion:

1.000000	1.000000	2.000000
1.000000	1.000000	2.000000
0.500000	0.500000	1.000000

The principal eigenvalue is: 3.000000

The Consistency Ratio is: 0.000000

The PCM HAS acceptable consistency.

Local weights of the sub-criterion (eigenvector):

0.400000 0.400000 0.200000

Criterion: C3;   Sub-criterion: C3-1   C3-2   C3-3

PCM of Sub-criterion w.r.t. Criterion:

1.000000   1.000000   0.750000

1.000000   1.000000   0.750000

1.333333   1.333333   1.000000

The principal eigenvalue is: 3.000000

The Consistency Ratio is: 0.000000

The PCM HAS acceptable consistency.

Local weights of the sub-criterion (eigenvector):

0.300000   0.300000   0.400000

Criterion: C4;   Sub-criterion: C4-1   C4-2   C4-3   C4-4

PCM of Sub-criterion w.r.t. Criterion:

1.000000   1.500000   1.500000   1.000000

0.666667   1.000000   1.000000   0.666667

0.666667   1.000000   1.000000   0.666667

1.000000   1.500000   1.500000   1.000000

The principal eigenvalue is: 4.000000

The Consistency Ratio is: 0.000000

The PCM HAS acceptable consistency.

Local weights of the sub-criterion (eigenvector):

0.300000 0.200000 0.200000 0.300000

Criterion: C5; Sub-criterion: C5-1 C5-2

PCM of Sub-criterion w.r.t. Criterion:

1.000000 0.250000

4.000000 1.000000

The principal eigenvalue is: 2.000000

The Consistency Ratio is: 0.000000

The PCM HAS acceptable consistency.

Local weights of the sub-criterion (eigenvector):

0.200000 0.800000

Criterion: C6; Sub-criterion: C6-1 C6-2 C6-3

PCM of Sub-criterion w.r.t. Criterion:

1.000000 0.600000 1.500000

1.666667 1.000000 2.500000

0.666667 0.400000 1.000000

The principal eigenvalue is: 3.000000

The Consistency Ratio is: 0.000000

The PCM HAS acceptable consistency.

Local weights of the sub-criterion (eigenvector):

0.300000 0.500000 0.200000

Criterion: C7; Sub-criterion: C7-1 C7-2 C7-3

PCM of Sub-criterion w.r.t. Criterion:

1.000000 1.333333 1.333333

0.750000 1.000000 1.000000

0.750000 1.000000 1.000000

The principal eigenvalue is: 3.000000

The Consistency Ratio is: 0.000000

The PCM HAS acceptable consistency.

Local weights of the sub-criterion (eigenvector):

0.400000   0.300000   0.300000

Alternatives: A1   A2   A3   A5

Leaf criteria: C2   C1-1   C1-2   C1-3   C3-1   C3-2   C3-3   C4-1   C4-2   C4-3   C4-4   C5-1   C5-2   C6-1   C6-2   C6-3   C7-1   C7-2   C7-3

PCM for Alternatives w.r.t. leaf criterion C2

1.000000	0.500000	0.333333	0.250000
2.000000	1.000000	0.666667	0.500000
3.000000	1.500000	1.000000	0.750000
4.000000	2.000000	1.333333	1.000000

The principal eigenvalue is: 4.000000

The Consistency Ratio is: 0.000000

The PCM HAS acceptable consistency.

Local weights of the alternatives (eigenvector):

0.100000   0.200000   0.300000   0.400000

PCM for Alternatives w.r.t. leaf criterion C1-1

1.000000	1.000000	0.666667	1.666667
1.000000	1.000000	0.666667	1.666667
1.500000	1.500000	1.000000	2.500000
0.600000	0.600000	0.400000	1.000000

The principal eigenvalue is: 4.000000

The Consistency Ratio is: 0.000000

The PCM HAS acceptable consistency.

Local weights of the alternatives (eigenvector):

0.243902 0.243902 0.365854 0.146341

PCM for Alternatives w.r.t. leaf criterion C1-2

1.000000	2.000000	3.000000	5.000000
0.500000	1.000000	1.500000	2.500000
0.333333	0.666667	1.000000	1.666667
0.200000	0.400000	0.600000	1.000000

The principal eigenvalue is: 4.000000

The Consistency Ratio is: 0.000000

The PCM HAS acceptable consistency.

Local weights of the alternatives (eigenvector):

0.491803 0.245902 0.163934 0.098361

PCM for Alternatives w.r.t. leaf criterion C1-3

1.000000	1.500000	0.500000	1.250000
0.666667	1.000000	0.333333	0.833333
2.000000	3.000000	1.000000	2.500000
0.800000	1.200000	0.400000	1.000000

The principal eigenvalue is: 4.000000

The Consistency Ratio is: 0.000000

The PCM HAS acceptable consistency.

Local weights of the alternatives (eigenvector):

0.223881 0.149254 0.447761 0.179104

PCM for Alternatives w.r.t. leaf criterion C3-1

1.000000	2.000000	0.333333	0.500000
0.500000	1.000000	0.166667	0.250000
3.000000	6.000000	1.000000	1.500000

2.000000 4.000000 0.666667 1.000000

The principal eigenvalue is: 4.000000

The Consistency Ratio is: 0.000000

The PCM HAS acceptable consistency.

Local weights of the alternatives (eigenvector):

0.153846 0.076923 0.461538 0.307692

PCM for Alternatives w.r.t. leaf criterion C3-2

1.000000 0.250000 0.500000 0.250000

4.000000 1.000000 2.000000 1.000000

2.000000 0.500000 1.000000 0.500000

4.000000 1.000000 2.000000 1.000000

The principal eigenvalue is: 4.000000

The Consistency Ratio is: 0.000000

The PCM HAS acceptable consistency.

Local weights of the alternatives (eigenvector):

0.090909 0.363636 0.181818 0.363636

PCM for Alternatives w.r.t. leaf criterion C3-3

1.000000	5.000000	2.500000	1.000000
0.200000	1.000000	0.500000	0.200000
0.400000	2.000000	1.000000	0.400000
1.000000	5.000000	2.500000	1.000000

The principal eigenvalue is: 4.000000

The Consistency Ratio is: 0.000000

The PCM HAS acceptable consistency.

Local weights of the alternatives (eigenvector):

0.384615   0.076923   0.153846   0.384615

PCM for Alternatives w.r.t. leaf criterion C4-1

1.000000	4.000000	4.000000	5.000000
0.250000	1.000000	1.000000	1.250000
0.250000	1.000000	1.000000	1.250000
0.200000	0.800000	0.800000	1.000000

The principal eigenvalue is: 4.000000

The Consistency Ratio is: 0.000000

The PCM HAS acceptable consistency.

Local weights of the alternatives (eigenvector):

0.588235 0.147059 0.147059 0.117647

PCM for Alternatives w.r.t. leaf criterion C4-2

1.000000	0.250000	0.500000	0.200000
4.000000	1.000000	2.000000	0.800000
2.000000	0.500000	1.000000	0.400000
5.000000	1.250000	2.500000	1.000000

The principal eigenvalue is: 4.000000

The Consistency Ratio is: 0.000000

The PCM HAS acceptable consistency.

Local weights of the alternatives (eigenvector):

0.083333 0.333333 0.166667 0.416667

PCM for Alternatives w.r.t. leaf criterion C4-3

1.000000	0.500000	0.200000	2.000000
2.000000	1.000000	0.400000	4.000000
5.000000	2.500000	1.000000	10.000000

0.500000 0.250000 0.100000 1.000000

The principal eigenvalue is: 4.000000

The Consistency Ratio is: 0.000000

The PCM HAS acceptable consistency.

Local weights of the alternatives (eigenvector):

0.117647 0.235294 0.588235 0.058824

PCM for Alternatives w.r.t. leaf criterion C4-4

1.000000	8.000000	6.000000	4.000000
0.125000	1.000000	0.750000	0.500000
0.166667	1.333333	1.000000	0.666667
0.250000	2.000000	1.500000	1.000000

The principal eigenvalue is: 4.000000

The Consistency Ratio is: 0.000000

The PCM HAS acceptable consistency.

Local weights of the alternatives (eigenvector):

0.648649 0.081081 0.108108 0.162162

PCM for Alternatives w.r.t. leaf criterion C5-1

1.000000	5.000000	3.000000	8.000000
0.200000	1.000000	0.600000	1.600000
0.333333	1.666667	1.000000	2.666667
0.125000	0.625000	0.375000	1.000000

The principal eigenvalue is: 4.000000

The Consistency Ratio is: 0.000000

The PCM HAS acceptable consistency.

Local weights of the alternatives (eigenvector):

0.603015 0.120603 0.201005 0.075377

PCM for Alternatives w.r.t. leaf criterion C5-2

1.000000	0.200000	0.500000	0.250000
5.000000	1.000000	2.500000	1.250000
2.000000	0.400000	1.000000	0.500000
4.000000	0.800000	2.000000	1.000000

The principal eigenvalue is: 4.000000

The Consistency Ratio is: 0.000000

The PCM HAS acceptable consistency.

Local weights of the alternatives (eigenvector):

0.083333 0.416667 0.166667 0.333333

PCM for Alternatives w.r.t. leaf criterion C6-1

1.000000	0.500000	3.000000	1.000000
2.000000	1.000000	6.000000	2.000000
0.333333	0.166667	1.000000	0.333333
1.000000	0.500000	3.000000	1.000000

The principal eigenvalue is: 4.000000

The Consistency Ratio is: 0.000000

The PCM HAS acceptable consistency.

Local weights of the alternatives (eigenvector):

0.230769 0.461538 0.076923 0.230769

PCM for Alternatives w.r.t. leaf criterion C6-2

1.000000	4.000000	4.000000	0.500000
0.250000	1.000000	1.000000	0.125000

0.250000 1.000000 1.000000 0.125000

2.000000 8.000000 8.000000 1.000000

The principal eigenvalue is: 4.000000

The Consistency Ratio is: 0.000000

The PCM HAS acceptable consistency.

Local weights of the alternatives (eigenvector):

0.285714 0.071429 0.071429 0.571429

PCM for Alternatives w.r.t. leaf criterion C6-3

1.000000 0.250000 0.500000 0.500000

4.000000 1.000000 2.000000 2.000000

2.000000 0.500000 1.000000 1.000000

2.000000 0.500000 1.000000 1.000000

The principal eigenvalue is: 4.000000

The Consistency Ratio is: 0.000000

The PCM HAS acceptable consistency.

Local weights of the alternatives (eigenvector):

0.111111 0.444444 0.222222 0.222222

PCM for Alternatives w.r.t. leaf criterion C7-1

1.000000	0.200000	0.500000	0.250000
5.000000	1.000000	2.500000	1.250000
2.000000	0.400000	1.000000	0.500000
4.000000	0.800000	2.000000	1.000000

The principal eigenvalue is: 4.000000

The Consistency Ratio is: 0.000000

The PCM HAS acceptable consistency.

Local weights of the alternatives (eigenvector):

0.083333 0.416667 0.166667 0.333333

PCM for Alternatives w.r.t. leaf criterion C7-2

1.000000	2.000000	0.500000	2.000000
0.500000	1.000000	0.250000	1.000000
2.000000	4.000000	1.000000	4.000000
0.500000	1.000000	0.250000	1.000000

The principal eigenvalue is: 4.000000

The Consistency Ratio is: 0.000000

The PCM HAS acceptable consistency.

Local weights of the alternatives (eigenvector):

0.250000   0.125000   0.500000   0.125000

PCM for Alternatives w.r.t. leaf criterion C7-3

1.000000	4.000000	2.000000	4.000000
0.250000	1.000000	0.500000	1.000000
0.500000	2.000000	1.000000	2.000000
0.250000	1.000000	0.500000	1.000000

The principal eigenvalue is: 4.000000

The Consistency Ratio is: 0.000000

The PCM HAS acceptable consistency.

Local weights of the alternatives (eigenvector):

0.500000   0.125000   0.250000   0.125000

Global weights of the leaf criterion:

0.146789   0.088073   0.088073   0.044037   0.049541   0.049541   0.066055   0.041284
0.027523   0.027523   0.041284   0.022018   0.088074   0.044037   0.073395   0.029358
0.029358   0.022018   0.022018

Alternatives' global weights:

0.257431 0.226750 0.239426 0.276392

The hierarchy structure:

7 T TOP C1 1 C2 0 C3 1 C4 1 C5 1 C6 1 C7 1

3 C1 T C1-1 0 C1-2 0 C1-3 0

3 C3 T C3-1 0 C3-2 0 C3-3 0

4 C4 T C4-1 0 C4-2 0 C4-3 0 C4-4 0

2 C5 T C5-1 0 C5-2 0

3 C6 T C6-1 0 C6-2 0 C6-3 0

3 C7 T C7-1 0 C7-2 0 C7-3 0

The number of alternatives:4

Criterion: T; Sub-criterion: C1 C2 C3 C4 C5 C6 C7

PCM of Sub-criterion w.r.t. Criterion:

1.000000	1.500000	1.333333	1.600000	2.000000	1.500000	3.000000
0.666667	1.000000	0.888889	1.066667	1.333333	1.000000	2.000000
0.750000	1.125000	1.000000	1.200000	1.500000	1.125000	2.250000
0.625000	0.937500	0.833333	1.000000	1.250000	0.937500	1.875000
0.500000	0.750000	0.666667	0.800000	1.000000	0.750000	1.500000
0.666667	1.000000	0.888889	1.066667	1.333333	1.000000	2.000000
0.333333	0.500000	0.444444	0.533333	0.666667	0.500000	1.000000

The principal eigenvalue is: 7.000000

The Consistency Ratio is: 0.000000

The PCM HAS acceptable consistency.

Local weights of the sub-criterion (eigenvector):

0.220183 0.146789 0.165138 0.137615 0.110092 0.146789 0.073394

Criterion: C1; Sub-criterion: C1-1 C1-2 C1-3

PCM of Sub-criterion w.r.t. Criterion:

1.000000	1.000000	2.000000
1.000000	1.000000	2.000000
0.500000	0.500000	1.000000

The principal eigenvalue is: 3.000000

The Consistency Ratio is: 0.000000

The PCM HAS acceptable consistency.

Local weights of the sub-criterion (eigenvector):

0.400000 0.400000 0.200000

Criterion: C3; Sub-criterion: C3-1 C3-2 C3-3

PCM of Sub-criterion w.r.t. Criterion:

1.000000 1.000000 0.750000

1.000000 1.000000 0.750000

1.333333 1.333333 1.000000

The principal eigenvalue is: 3.000000

The Consistency Ratio is: 0.000000

The PCM HAS acceptable consistency.

Local weights of the sub-criterion (eigenvector):

0.300000 0.300000 0.400000

Criterion: C4; Sub-criterion: C4-1 C4-2 C4-3 C4-4

PCM of Sub-criterion w.r.t. Criterion:

1.000000 1.500000 1.500000 1.000000

0.666667 1.000000 1.000000 0.666667

0.666667 1.000000 1.000000 0.666667

1.000000 1.500000 1.500000 1.000000

The principal eigenvalue is: 4.000000

The Consistency Ratio is: 0.000000

The PCM HAS acceptable consistency.

Local weights of the sub-criterion (eigenvector):

0.300000 0.200000 0.200000 0.300000

Criterion: C5; Sub-criterion: C5-1 C5-2

PCM of Sub-criterion w.r.t. Criterion:

1.000000 0.250000

4.000000 1.000000

The principal eigenvalue is: 2.000000

The Consistency Ratio is: 0.000000

The PCM HAS acceptable consistency.

Local weights of the sub-criterion (eigenvector):

0.200000 0.800000

Criterion: C6; Sub-criterion: C6-1 C6-2 C6-3

PCM of Sub-criterion w.r.t. Criterion:

1.000000 0.600000 1.500000

1.666667 1.000000 2.500000

0.666667 0.400000 1.000000

The principal eigenvalue is: 3.000000

The Consistency Ratio is: 0.000000

The PCM HAS acceptable consistency.

Local weights of the sub-criterion (eigenvector):

0.300000 0.500000 0.200000

Criterion: C7; Sub-criterion: C7-1 C7-2 C7-3

PCM of Sub-criterion w.r.t. Criterion:

1.000000 1.333333 1.333333

0.750000 1.000000 1.000000

0.750000 1.000000 1.000000

The principal eigenvalue is: 3.000000

The Consistency Ratio is: 0.000000

The PCM HAS acceptable consistency.

Local weights of the sub-criterion (eigenvector):

0.400000   0.300000   0.300000

Alternatives: A1   A2   A3   A4

Leaf criteria: C2   C1-1   C1-2   C1-3   C3-1   C3-2   C3-3   C4-1   C4-2   C4-3   C4-4   C5-1   C5-2   C6-1   C6-2   C6-3   C7-1   C7-2   C7-3

PCM for Alternatives w.r.t. leaf criterion C2

1.000000	0.500000	0.333333	0.250000
2.000000	1.000000	0.666667	0.500000
3.000000	1.500000	1.000000	0.750000
4.000000	2.000000	1.333333	1.000000

The principal eigenvalue is: 4.000000

The Consistency Ratio is: 0.000000

The PCM HAS acceptable consistency.

Local weights of the alternatives (eigenvector):

0.100000   0.200000   0.300000   0.400000

PCM for Alternatives w.r.t. leaf criterion C1-1

1.000000	1.000000	0.666667	1.250000
1.000000	1.000000	0.666667	1.250000
1.500000	1.500000	1.000000	1.875000
0.800000	0.800000	0.533333	1.000000

The principal eigenvalue is: 4.000000

The Consistency Ratio is: 0.000000

The PCM HAS acceptable consistency.

Local weights of the alternatives (eigenvector):

0.232558 0.232558 0.348837 0.186047

PCM for Alternatives w.r.t. leaf criterion C1-2

1.000000	2.000000	3.000000	4.000000
0.500000	1.000000	1.500000	2.000000
0.333333	0.666667	1.000000	1.333333
0.250000	0.500000	0.750000	1.000000

The principal eigenvalue is: 4.000000

The Consistency Ratio is: 0.000000

The PCM HAS acceptable consistency.

Local weights of the alternatives (eigenvector):

0.480000 0.240000 0.160000 0.120000

PCM for Alternatives w.r.t. leaf criterion C1-3

1.000000	1.500000	0.500000	2.000000
0.666667	1.000000	0.333333	1.333333
2.000000	3.000000	1.000000	4.000000
0.500000	0.750000	0.250000	1.000000

The principal eigenvalue is: 4.000000

The Consistency Ratio is: 0.000000

The PCM HAS acceptable consistency.

Local weights of the alternatives (eigenvector):

0.240000 0.160000 0.480000 0.120000

PCM for Alternatives w.r.t. leaf criterion C3-1

1.000000	2.000000	0.333333	0.250000
0.500000	1.000000	0.166667	0.125000
3.000000	6.000000	1.000000	0.750000

4.000000   8.000000   1.333333   1.000000

The principal eigenvalue is: 4.000000

The Consistency Ratio is: 0.000000

The PCM HAS acceptable consistency.

Local weights of the alternatives (eigenvector):

0.117647   0.058824   0.352941   0.470588

PCM for Alternatives w.r.t. leaf criterion C3-2

1.000000   0.250000   0.500000   4.000000

4.000000   1.000000   2.000000   16.000000

2.000000   0.500000   1.000000   8.000000

0.250000   0.062500   0.125000   1.000000

The principal eigenvalue is: 4.000000

The Consistency Ratio is: 0.000000

The PCM HAS acceptable consistency.

Local weights of the alternatives (eigenvector):

0.137931   0.551724   0.275862   0.034483

PCM for Alternatives w.r.t. leaf criterion C3-3

1.000000	5.000000	2.500000	0.333333
0.200000	1.000000	0.500000	0.066667
0.400000	2.000000	1.000000	0.133333
3.000000	15.000000	7.500000	1.000000

The principal eigenvalue is: 4.000000

The Consistency Ratio is: 0.000000

The PCM HAS acceptable consistency.

Local weights of the alternatives (eigenvector):

0.217391 0.043478 0.086957 0.652174

PCM for Alternatives w.r.t. leaf criterion C4-1

1.000000	4.000000	4.000000	0.500000
0.250000	1.000000	1.000000	0.125000
0.250000	1.000000	1.000000	0.125000
2.000000	8.000000	8.000000	1.000000

The principal eigenvalue is: 4.000000

The Consistency Ratio is: 0.000000

The PCM HAS acceptable consistency.

Local weights of the alternatives (eigenvector):

0.285714 0.071429 0.071429 0.571429

PCM for Alternatives w.r.t. leaf criterion C4-2

1.000000	0.250000	0.500000	2.000000
4.000000	1.000000	2.000000	8.000000
2.000000	0.500000	1.000000	4.000000
0.500000	0.125000	0.250000	1.000000

The principal eigenvalue is: 4.000000

The Consistency Ratio is: 0.000000

The PCM HAS acceptable consistency.

Local weights of the alternatives (eigenvector):

0.133333 0.533333 0.266667 0.066667

PCM for Alternatives w.r.t. leaf criterion C4-3

1.000000	0.500000	0.200000	0.250000
2.000000	1.000000	0.400000	0.500000
5.000000	2.500000	1.000000	1.250000

4.000000 2.000000 0.800000 1.000000

The principal eigenvalue is: 4.000000

The Consistency Ratio is: 0.000000

The PCM HAS acceptable consistency.

Local weights of the alternatives (eigenvector):

0.083333 0.166667 0.416667 0.333333

PCM for Alternatives w.r.t. leaf criterion C4-4

1.000000 8.000000 6.000000 0.500000

0.125000 1.000000 0.750000 0.062500

0.166667 1.333333 1.000000 0.083333

2.000000 16.000000 12.000000 1.000000

The principal eigenvalue is: 4.000000

The Consistency Ratio is: 0.000000

The PCM HAS acceptable consistency.

Local weights of the alternatives (eigenvector):

0.303797 0.037975 0.050633 0.607595

PCM for Alternatives w.r.t. leaf criterion C5-1

1.000000	5.000000	3.000000	2.000000
0.200000	1.000000	0.600000	0.400000
0.333333	1.666667	1.000000	0.666667
0.500000	2.500000	1.500000	1.000000

The principal eigenvalue is: 4.000000

The Consistency Ratio is: 0.000000

The PCM HAS acceptable consistency.

Local weights of the alternatives (eigenvector):

0.491803 0.098361 0.163934 0.245902

PCM for Alternatives w.r.t. leaf criterion C5-2

1.000000	0.200000	0.500000	4.000000
5.000000	1.000000	2.500000	20.000000
2.000000	0.400000	1.000000	8.000000
0.250000	0.050000	0.125000	1.000000

The principal eigenvalue is: 4.000000

The Consistency Ratio is: 0.000000

The PCM HAS acceptable consistency.

Local weights of the alternatives (eigenvector):

0.121212 0.606061 0.242424 0.030303

PCM for Alternatives w.r.t. leaf criterion C6-1

1.000000	0.500000	3.000000	4.000000
2.000000	1.000000	6.000000	8.000000
0.333333	0.166667	1.000000	1.333333
0.250000	0.125000	0.750000	1.000000

The principal eigenvalue is: 4.000000

The Consistency Ratio is: 0.000000

The PCM HAS acceptable consistency.

Local weights of the alternatives (eigenvector):

0.279070 0.558140 0.093023 0.069767

PCM for Alternatives w.r.t. leaf criterion C6-2

1.000000	4.000000	4.000000	0.250000
0.250000	1.000000	1.000000	0.062500

0.250000 1.000000 1.000000 0.062500

4.000000 16.000000 16.000000 1.000000

The principal eigenvalue is: 4.000000

The Consistency Ratio is: 0.000000

The PCM HAS acceptable consistency.

Local weights of the alternatives (eigenvector):

0.181818 0.045455 0.045455 0.727273

PCM for Alternatives w.r.t. leaf criterion C6-3

1.000000 0.250000 0.500000 0.250000

4.000000 1.000000 2.000000 1.000000

2.000000 0.500000 1.000000 0.500000

4.000000 1.000000 2.000000 1.000000

The principal eigenvalue is: 4.000000

The Consistency Ratio is: 0.000000

The PCM HAS acceptable consistency.

Local weights of the alternatives (eigenvector):

0.090909 0.363636 0.181818 0.363636

PCM for Alternatives w.r.t. leaf criterion C7-1

1.000000	0.200000	0.500000	2.000000
5.000000	1.000000	2.500000	10.000000
2.000000	0.400000	1.000000	4.000000
0.500000	0.100000	0.250000	1.000000

The principal eigenvalue is: 4.000000

The Consistency Ratio is: 0.000000

The PCM HAS acceptable consistency.

Local weights of the alternatives (eigenvector):

0.117647 0.588235 0.235294 0.058824

PCM for Alternatives w.r.t. leaf criterion C7-2

1.000000	2.000000	0.500000	1.000000
0.500000	1.000000	0.250000	0.500000
2.000000	4.000000	1.000000	2.000000
1.000000	2.000000	0.500000	1.000000

The principal eigenvalue is: 4.000000

The Consistency Ratio is: 0.000000

The PCM HAS acceptable consistency.

Local weights of the alternatives (eigenvector):

0.222222   0.111111   0.444444   0.222222

PCM for Alternatives w.r.t. leaf criterion C7-3

1.000000	4.000000	2.000000	0.500000
0.250000	1.000000	0.500000	0.125000
0.500000	2.000000	1.000000	0.250000
2.000000	8.000000	4.000000	1.000000

The principal eigenvalue is: 4.000000

The Consistency Ratio is: 0.000000

The PCM HAS acceptable consistency.

Local weights of the alternatives (eigenvector):

0.266667   0.066667   0.133333   0.533333

Global weights of the leaf criterion:

0.146789   0.088073   0.088073   0.044037   0.049541   0.049541   0.066055   0.041284
0.027523   0.027523   0.041284   0.022018   0.088074   0.044037   0.073395   0.029358
0.029358   0.022018   0.022018

Alternatives' global weights:

0.209352 0.250243 0.228092 0.312312
