# Supplementary material for: Market Competitiveness Evaluation of Mechanical Equipment with a Pairwise Comparisons Hierarchical Model
Source: PLoS One. 2016 Jan 19;11(1):e0146862. doi: 10.1371/journal.pone.0146862 (PMC4718694; doi:10.1371/journal.pone.0146862)
Supplement: S2 File — (PDF) [file pone.0146862.s002.pdf]

The hierarchy structure:

7 T TOP C1 1 C2 0 C3 1 C4 1 C5 1 C6 1 C7 1

3 C1 T C1-1 0 C1-2 0 C1-3 0

3 C3 T C3-1 0 C3-2 0 C3-3 0

4 C4 T C4-1 0 C4-2 0 C4-3 0 C4-4 0

2 C5 T C5-1 0 C5-2 0

3 C6 T C6-1 0 C6-2 0 C6-3 0

3 C7 T C7-1 0 C7-2 0 C7-3 0

The number of alternatives:5

Criterion: T; Sub-criterion: C1 C2 C3 C4 C5 C6 C7

PCM of Sub-criterion w.r.t. Criterion:

1.000000	1.500000	1.333333	1.600000	2.000000	1.500000	3.000000
0.666667	1.000000	0.888889	1.066667	1.333333	1.000000	2.000000
0.750000	1.125000	1.000000	1.200000	1.500000	1.125000	2.250000
0.625000	0.937500	0.833333	1.000000	1.250000	0.937500	1.875000
0.500000	0.750000	0.666667	0.800000	1.000000	0.750000	1.500000
0.666667	1.000000	0.888889	1.066667	1.333333	1.000000	2.000000
0.333333	0.500000	0.444444	0.533333	0.666667	0.500000	1.000000

The PCM HAS acceptable consistency.

Local weights of the sub-criterion:

0.220183 0.146789 0.165138 0.137615 0.110092 0.146789 0.073394

Criterion: C1; Sub-criterion: C1-1 C1-2 C1-3

PCM of Sub-criterion w.r.t. Criterion:

1.000000 1.000000 2.000000

1.000000 1.000000 2.000000

0.500000 0.500000 1.000000

The PCM HAS acceptable consistency.

Local weights of the sub-criterion:

0.400000 0.400000 0.200000

Criterion: C3; Sub-criterion: C3-1 C3-2 C3-3

PCM of Sub-criterion w.r.t. Criterion:

1.000000 1.000000 0.750000

1.000000 1.000000 0.750000

1.333333 1.333333 1.000000

The PCM HAS acceptable consistency.

Local weights of the sub-criterion:

0.300000 0.300000 0.400000

Criterion: C4; Sub-criterion: C4-1 C4-2 C4-3 C4-4

PCM of Sub-criterion w.r.t. Criterion:

1.000000	1.500000	1.500000	1.000000
0.666667	1.000000	1.000000	0.666667
0.666667	1.000000	1.000000	0.666667
1.000000	1.500000	1.500000	1.000000

The PCM HAS acceptable consistency.

Local weights of the sub-criterion:

0.300000 0.200000 0.200000 0.300000

Criterion: C5; Sub-criterion: C5-1 C5-2

PCM of Sub-criterion w.r.t. Criterion:

1.000000	0.250000
4.000000	1.000000

The PCM HAS acceptable consistency.

Local weights of the sub-criterion:

0.200000 0.800000

Criterion: C6; Sub-criterion: C6-1 C6-2 C6-3

PCM of Sub-criterion w.r.t. Criterion:

1.000000	0.600000	1.500000
1.666667	1.000000	2.500000
0.666667	0.400000	1.000000

The PCM HAS acceptable consistency.

Local weights of the sub-criterion:

0.300000 0.500000 0.200000

Criterion: C7; Sub-criterion: C7-1 C7-2 C7-3

PCM of Sub-criterion w.r.t. Criterion:

1.000000	1.333333	1.333333
0.750000	1.000000	1.000000
0.750000	1.000000	1.000000

The PCM HAS acceptable consistency.

Local weights of the sub-criterion:

0.400000 0.300000 0.300000

Alternatives: A1 A2 A3 A4 A5

Leaf criteria: C2 C1-1 C1-2 C1-3 C3-1 C3-2 C3-3 C4-1 C4-2 C4-3 C4-4 C5-1 C5-2 C6-1 C6-2 C6-3 C7-1 C7-2 C7-3

PCM for Alternatives w.r.t. leaf criterion C2

1.000000	0.500000	0.333333	0.250000	0.250000
2.000000	1.000000	0.666667	0.500000	0.500000
3.000000	1.500000	1.000000	0.750000	0.750000
4.000000	2.000000	1.333333	1.000000	1.000000
4.000000	2.000000	1.333333	1.000000	1.000000

The PCM HAS acceptable consistency.

Local weights of the alternatives:

1.000000 2.000000 2.999998 3.999998 3.999998

PCM for Alternatives w.r.t. leaf criterion C1-1

1.000000	1.000000	0.666667	1.250000	1.666667
1.000000	1.000000	0.666667	1.250000	1.666667
1.500000	1.500000	1.000000	1.875000	2.500000
0.800000	0.800000	0.533333	1.000000	1.333333
0.600000	0.600000	0.400000	0.750000	1.000000

The PCM HAS acceptable consistency.

Local weights of the alternatives:

1.666667   1.666667   2.500002   1.333334   1.000000

PCM for Alternatives w.r.t. leaf criterion C1-2

1.000000	2.000000	3.000000	4.000000	5.000000
0.500000	1.000000	1.500000	2.000000	2.500000
0.333333	0.666667	1.000000	1.333333	1.666667
0.250000	0.500000	0.750000	1.000000	1.250000
0.200000	0.400000	0.600000	0.800000	1.000000

The PCM HAS acceptable consistency.

Local weights of the alternatives:

5.000002   2.500002   1.666667   1.250001   1.000000

PCM for Alternatives w.r.t. leaf criterion C1-3

1.000000	1.500000	0.500000	2.000000	1.250000
0.666667	1.000000	0.333333	1.333333	0.833333
2.000000	3.000000	1.000000	4.000000	2.500000
0.500000	0.750000	0.250000	1.000000	0.625000
0.800000	1.200000	0.400000	1.600000	1.000000

The PCM HAS acceptable consistency.

Local weights of the alternatives:

2.000002 1.333335 4.000002 1.000000 1.600000

PCM for Alternatives w.r.t. leaf criterion C3-1

1.000000	2.000000	0.333333	0.250000	0.500000
0.500000	1.000000	0.166667	0.125000	0.250000
3.000000	6.000000	1.000000	0.750000	1.500000
4.000000	8.000000	1.333333	1.000000	2.000000
2.000000	4.000000	0.666667	0.500000	1.000000

The PCM HAS acceptable consistency.

Local weights of the alternatives:

2.000000 1.000000 6.000003 8.000003 4.000003

PCM for Alternatives w.r.t. leaf criterion C3-2

1.000000	0.250000	0.500000	4.000000	0.250000
4.000000	1.000000	2.000000	16.000000	1.000000
2.000000	0.500000	1.000000	8.000000	0.500000
0.250000	0.062500	0.125000	1.000000	0.062500
4.000000	1.000000	2.000000	16.000000	1.000000

The PCM HAS acceptable consistency.

Local weights of the alternatives:

4.000012 16.000049 8.000024 1.000000 16.000049

PCM for Alternatives w.r.t. leaf criterion C3-3

1.000000	5.000000	2.500000	0.333333	1.000000
0.200000	1.000000	0.500000	0.066667	0.200000
0.400000	2.000000	1.000000	0.133333	0.400000
3.000000	15.000000	7.500000	1.000000	3.000000
1.000000	5.000000	2.500000	0.333333	1.000000

The PCM HAS acceptable consistency.

Local weights of the alternatives:

5.000000 1.000000 2.000000 15.000000 5.000000

PCM for Alternatives w.r.t. leaf criterion C4-1

1.000000	4.000000	4.000000	0.500000	5.000000
0.250000	1.000000	1.000000	0.125000	1.250000
0.250000	1.000000	1.000000	0.125000	1.250000
2.000000	8.000000	8.000000	1.000000	10.000000
0.200000	0.800000	0.800000	0.100000	1.000000

The PCM HAS acceptable consistency.

Local weights of the alternatives:

4.999998 1.250000 1.250000 9.999995 1.000000

PCM for Alternatives w.r.t. leaf criterion C4-2

1.000000	0.250000	0.500000	2.000000	0.200000
4.000000	1.000000	2.000000	8.000000	0.800000
2.000000	0.500000	1.000000	4.000000	0.400000
0.500000	0.125000	0.250000	1.000000	0.100000
5.000000	1.250000	2.500000	10.000000	1.000000

The PCM HAS acceptable consistency.

Local weights of the alternatives:

2.000000 8.000004 4.000004 1.000000 10.000004

PCM for Alternatives w.r.t. leaf criterion C4-3

1.000000	0.500000	0.200000	0.250000	2.000000
2.000000	1.000000	0.400000	0.500000	4.000000
5.000000	2.500000	1.000000	1.250000	10.000000
4.000000	2.000000	0.800000	1.000000	8.000000
0.500000	0.250000	0.100000	0.125000	1.000000

The PCM HAS acceptable consistency.

Local weights of the alternatives:

2.000000 4.000004 10.000004 8.000004 1.000000

PCM for Alternatives w.r.t. leaf criterion C4-4

1.000000	8.000000	6.000000	0.500000	4.000000
0.125000	1.000000	0.750000	0.062500	0.500000
0.166667	1.333333	1.000000	0.083333	0.666667
2.000000	16.000000	12.000000	1.000000	8.000000
0.250000	2.000000	1.500000	0.125000	1.000000

The PCM HAS acceptable consistency.

Local weights of the alternatives:

7.999994 1.000000 1.333333 15.999987 1.999997

PCM for Alternatives w.r.t. leaf criterion C5-1

1.000000	5.000000	3.000000	2.000000	8.000000
0.200000	1.000000	0.600000	0.400000	1.600000
0.333333	1.666667	1.000000	0.666667	2.666667
0.500000	2.500000	1.500000	1.000000	4.000000
0.125000	0.625000	0.375000	0.250000	1.000000

The PCM HAS acceptable consistency.

Local weights of the alternatives:

8.000011 1.600002 2.666671 4.000005 1.000000

PCM for Alternatives w.r.t. leaf criterion C5-2

1.000000	0.200000	0.500000	4.000000	0.250000
5.000000	1.000000	2.500000	20.000000	1.250000
2.000000	0.400000	1.000000	8.000000	0.500000
0.250000	0.050000	0.125000	1.000000	0.062500
4.000000	0.800000	2.000000	16.000000	1.000000

The PCM HAS acceptable consistency.

Local weights of the alternatives:

4.000006 20.000044 8.000019 1.000000 16.000032

PCM for Alternatives w.r.t. leaf criterion C6-1

1.000000	0.500000	3.000000	4.000000	1.000000
2.000000	1.000000	6.000000	8.000000	2.000000
0.333333	0.166667	1.000000	1.333333	0.333333
0.250000	0.125000	0.750000	1.000000	0.250000
1.000000	0.500000	3.000000	4.000000	1.000000

The PCM HAS acceptable consistency.

Local weights of the alternatives:

4.000003 8.000006 1.333335 1.000000 4.000003

PCM for Alternatives w.r.t. leaf criterion C6-2

1.000000	4.000000	4.000000	0.250000	0.500000
0.250000	1.000000	1.000000	0.062500	0.125000
0.250000	1.000000	1.000000	0.062500	0.125000
4.000000	16.000000	16.000000	1.000000	2.000000
2.000000	8.000000	8.000000	0.500000	1.000000

The PCM HAS acceptable consistency.

Local weights of the alternatives:

3.999993 1.000000 1.000000 15.999976 7.999990

PCM for Alternatives w.r.t. leaf criterion C6-3

1.000000	0.250000	0.500000	0.250000	0.500000
4.000000	1.000000	2.000000	1.000000	2.000000
2.000000	0.500000	1.000000	0.500000	1.000000
4.000000	1.000000	2.000000	1.000000	2.000000
2.000000	0.500000	1.000000	0.500000	1.000000

The PCM HAS acceptable consistency.

Local weights of the alternatives:

1.000000 4.000002 2.000002 4.000002 2.000002

PCM for Alternatives w.r.t. leaf criterion C7-1

1.000000	0.200000	0.500000	2.000000	0.250000
5.000000	1.000000	2.500000	10.000000	1.250000
2.000000	0.400000	1.000000	4.000000	0.500000
0.500000	0.100000	0.250000	1.000000	0.125000
4.000000	0.800000	2.000000	8.000000	1.000000

The PCM HAS acceptable consistency.

Local weights of the alternatives:

2.000000 10.000004 4.000004 1.000000 8.000004

PCM for Alternatives w.r.t. leaf criterion C7-2

1.000000	2.000000	0.500000	1.000000	2.000000
0.500000	1.000000	0.250000	0.500000	1.000000
2.000000	4.000000	1.000000	2.000000	4.000000
1.000000	2.000000	0.500000	1.000000	2.000000
0.500000	1.000000	0.250000	0.500000	1.000000

The PCM HAS acceptable consistency.

Local weights of the alternatives:

2.000000 1.000000 4.000002 2.000000 1.000000

PCM for Alternatives w.r.t. leaf criterion C7-3

1.000000	4.000000	2.000000	0.500000	4.000000
0.250000	1.000000	0.500000	0.125000	1.000000
0.500000	2.000000	1.000000	0.250000	2.000000
2.000000	8.000000	4.000000	1.000000	8.000000
0.250000	1.000000	0.500000	0.125000	1.000000

The PCM HAS acceptable consistency.

Local weights of the alternatives:

4.000002 1.000000 2.000002 8.000005 1.000000

Global weights of the leaf criterion:

0.146789 0.088073 0.088073 0.044037 0.049541 0.049541 0.066055 0.041284
0.027523 0.027523 0.041284 0.022018 0.088074 0.044037 0.073395 0.029358
0.029358 0.022018 0.022018

Alternatives' global weights:

2.756191 2.601603 2.807889 3.104646 3.214869

The hierarchy structure:

7 T TOP C1 1 C2 0 C3 1 C4 1 C5 1 C6 1 C7 1

3 C1 T C1-1 0 C1-2 0 C1-3 0

3 C3 T C3-1 0 C3-2 0 C3-3 0

4 C4 T C4-1 0 C4-2 0 C4-3 0 C4-4 0

2 C5 T C5-1 0 C5-2 0

3 C6 T C6-1 0 C6-2 0 C6-3 0

3 C7 T C7-1 0 C7-2 0 C7-3 0

The number of alternatives:4

Criterion: T; Sub-criterion: C1 C2 C3 C4 C5 C6 C7

PCM of Sub-criterion w.r.t. Criterion:

1.000000	1.500000	1.333333	1.600000	2.000000	1.500000	3.000000
0.666667	1.000000	0.888889	1.066667	1.333333	1.000000	2.000000
0.750000	1.125000	1.000000	1.200000	1.500000	1.125000	2.250000
0.625000	0.937500	0.833333	1.000000	1.250000	0.937500	1.875000
0.500000	0.750000	0.666667	0.800000	1.000000	0.750000	1.500000
0.666667	1.000000	0.888889	1.066667	1.333333	1.000000	2.000000
0.333333	0.500000	0.444444	0.533333	0.666667	0.500000	1.000000

The PCM HAS acceptable consistency.

Local weights of the sub-criterion:

0.220183 0.146789 0.165138 0.137615 0.110092 0.146789 0.073394

Criterion: C1; Sub-criterion: C1-1 C1-2 C1-3

PCM of Sub-criterion w.r.t. Criterion:

1.000000 1.000000 2.000000

1.000000 1.000000 2.000000

0.500000 0.500000 1.000000

The PCM HAS acceptable consistency.

Local weights of the sub-criterion:

0.400000 0.400000 0.200000

Criterion: C3; Sub-criterion: C3-1 C3-2 C3-3

PCM of Sub-criterion w.r.t. Criterion:

1.000000 1.000000 0.750000

1.000000 1.000000 0.750000

1.333333 1.333333 1.000000

The PCM HAS acceptable consistency.

Local weights of the sub-criterion:

0.300000 0.300000 0.400000

Criterion: C4; Sub-criterion: C4-1 C4-2 C4-3 C4-4

PCM of Sub-criterion w.r.t. Criterion:

1.000000	1.500000	1.500000	1.000000
0.666667	1.000000	1.000000	0.666667
0.666667	1.000000	1.000000	0.666667
1.000000	1.500000	1.500000	1.000000

The PCM HAS acceptable consistency.

Local weights of the sub-criterion:

0.300000 0.200000 0.200000 0.300000

Criterion: C5; Sub-criterion: C5-1 C5-2

PCM of Sub-criterion w.r.t. Criterion:

1.000000	0.250000
4.000000	1.000000

The PCM HAS acceptable consistency.

Local weights of the sub-criterion:

0.200000 0.800000

Criterion: C6; Sub-criterion: C6-1 C6-2 C6-3

PCM of Sub-criterion w.r.t. Criterion:

1.000000 0.600000 1.500000

1.666667 1.000000 2.500000

0.666667 0.400000 1.000000

The PCM HAS acceptable consistency.

Local weights of the sub-criterion:

0.300000 0.500000 0.200000

Criterion: C7; Sub-criterion: C7-1 C7-2 C7-3

PCM of Sub-criterion w.r.t. Criterion:

1.000000 1.333333 1.333333

0.750000 1.000000 1.000000

0.750000 1.000000 1.000000

The PCM HAS acceptable consistency.

Local weights of the sub-criterion:

0.400000 0.300000 0.300000

Alternatives: A2 A3 A4 A5

Leaf criteria: C2 C1-1 C1-2 C1-3 C3-1 C3-2 C3-3 C4-1 C4-2 C4-3 C4-4 C5-1 C5-2 C6-1 C6-2 C6-3 C7-1 C7-2 C7-3

PCM for Alternatives w.r.t. leaf criterion C2

1.000000 0.666667 0.500000 0.500000

1.500000 1.000000 0.750000 0.750000

2.000000 1.333333 1.000000 1.000000

2.000000 1.333333 1.000000 1.000000

The PCM HAS acceptable consistency.

Local weights of the alternatives:

1.000000 1.500001 2.000000 2.000000

PCM for Alternatives w.r.t. leaf criterion C1-1

1.000000 0.666667 1.250000 1.666667

1.500000	1.000000	1.875000	2.500000
0.800000	0.533333	1.000000	1.333333
0.600000	0.400000	0.750000	1.000000

The PCM HAS acceptable consistency.

Local weights of the alternatives:

1.666666 2.499998 1.333332 1.000000

PCM for Alternatives w.r.t. leaf criterion C1-2

1.000000	1.500000	2.000000	2.500000
0.666667	1.000000	1.333333	1.666667
0.500000	0.750000	1.000000	1.250000
0.400000	0.600000	0.800000	1.000000

The PCM HAS acceptable consistency.

Local weights of the alternatives:

2.500000 1.666667 1.250001 1.000000

PCM for Alternatives w.r.t. leaf criterion C1-3

1.000000	0.333333	1.333333	0.833333
3.000000	1.000000	4.000000	2.500000

0.750000	0.250000	1.000000	0.625000
1.200000	0.400000	1.600000	1.000000

The PCM HAS acceptable consistency.

Local weights of the alternatives:

1.333333 3.999998 1.000000 1.600000

PCM for Alternatives w.r.t. leaf criterion C3-1

1.000000	0.166667	0.125000	0.250000
6.000000	1.000000	0.750000	1.500000
8.000000	1.333333	1.000000	2.000000
4.000000	0.666667	0.500000	1.000000

The PCM HAS acceptable consistency.

Local weights of the alternatives:

1.000000 6.000011 8.000015 4.000007

PCM for Alternatives w.r.t. leaf criterion C3-2

1.000000	2.000000	16.000000	1.000000
0.500000	1.000000	8.000000	0.500000
0.062500	0.125000	1.000000	0.062500
1.000000	2.000000	16.000000	1.000000

The PCM HAS acceptable consistency.

Local weights of the alternatives:

15.999987 7.999993 1.000000 15.999987

PCM for Alternatives w.r.t. leaf criterion C3-3

1.000000	0.500000	0.066667	0.200000
2.000000	1.000000	0.133333	0.400000
15.000000	7.500000	1.000000	3.000000
5.000000	2.500000	0.333333	1.000000

The PCM HAS acceptable consistency.

Local weights of the alternatives:

1.000000 2.000000 15.000003 5.000000

PCM for Alternatives w.r.t. leaf criterion C4-1

1.000000	1.000000	0.125000	1.250000
1.000000	1.000000	0.125000	1.250000
8.000000	8.000000	1.000000	10.000000
0.800000	0.800000	0.100000	1.000000

The PCM HAS acceptable consistency.

Local weights of the alternatives:

1.250001 1.250001 10.000008 1.000000

PCM for Alternatives w.r.t. leaf criterion C4-2

1.000000	2.000000	8.000000	0.800000
0.500000	1.000000	4.000000	0.400000
0.125000	0.250000	1.000000	0.100000
1.250000	2.500000	10.000000	1.000000

The PCM HAS acceptable consistency.

Local weights of the alternatives:

8.000013 4.000008 1.000000 10.000017

PCM for Alternatives w.r.t. leaf criterion C4-3

1.000000	0.400000	0.500000	4.000000
2.500000	1.000000	1.250000	10.000000
2.000000	0.800000	1.000000	8.000000
0.250000	0.100000	0.125000	1.000000

The PCM HAS acceptable consistency.

Local weights of the alternatives:

4.000008 10.000017 8.000013 1.000000

PCM for Alternatives w.r.t. leaf criterion C4-4

1.000000 0.750000 0.062500 0.500000

1.333333 1.000000 0.083333 0.666667

16.000000 12.000000 1.000000 8.000000

2.000000 1.500000 0.125000 1.000000

The PCM HAS acceptable consistency.

Local weights of the alternatives:

1.000000 1.333334 16.000005 2.000000

PCM for Alternatives w.r.t. leaf criterion C5-1

1.000000 0.600000 0.400000 1.600000

1.666667 1.000000 0.666667 2.666667

2.500000 1.500000 1.000000 4.000000

0.625000 0.375000 0.250000 1.000000

The PCM HAS acceptable consistency.

Local weights of the alternatives:

1.600002 2.666669 4.000004 1.000000

PCM for Alternatives w.r.t. leaf criterion C5-2

1.000000	2.500000	20.000000	1.250000
0.400000	1.000000	8.000000	0.500000
0.050000	0.125000	1.000000	0.062500
0.800000	2.000000	16.000000	1.000000

The PCM HAS acceptable consistency.

Local weights of the alternatives:

20.000050 8.000021 1.000000 16.000036

PCM for Alternatives w.r.t. leaf criterion C6-1

1.000000	6.000000	8.000000	2.000000
0.166667	1.000000	1.333333	0.333333
0.125000	0.750000	1.000000	0.250000
0.500000	3.000000	4.000000	1.000000

The PCM HAS acceptable consistency.

Local weights of the alternatives:

8.000003 1.333334 1.000000 4.000003

PCM for Alternatives w.r.t. leaf criterion C6-2

1.000000 1.000000 0.062500 0.125000

1.000000 1.000000 0.062500 0.125000

16.000000 16.000000 1.000000 2.000000

8.000000 8.000000 0.500000 1.000000

The PCM HAS acceptable consistency.

Local weights of the alternatives:

1.000000 1.000000 15.999987 7.999993

PCM for Alternatives w.r.t. leaf criterion C6-3

1.000000 2.000000 1.000000 2.000000

0.500000 1.000000 0.500000 1.000000

1.000000 2.000000 1.000000 2.000000

0.500000 1.000000 0.500000 1.000000

The PCM HAS acceptable consistency.

Local weights of the alternatives:

2.000000 1.000000 2.000000 1.000000

PCM for Alternatives w.r.t. leaf criterion C7-1

1.000000 2.500000 10.000000 1.250000

0.400000 1.000000 4.000000 0.500000

0.100000 0.250000 1.000000 0.125000

0.800000 2.000000 8.000000 1.000000

The PCM HAS acceptable consistency.

Local weights of the alternatives:

10.000017 4.000008 1.000000 8.000013

PCM for Alternatives w.r.t. leaf criterion C7-2

1.000000 0.250000 0.500000 1.000000

4.000000 1.000000 2.000000 4.000000

2.000000 0.500000 1.000000 2.000000

1.000000 0.250000 0.500000 1.000000

The PCM HAS acceptable consistency.

Local weights of the alternatives:

1.000000 3.999997 1.999998 1.000000

PCM for Alternatives w.r.t. leaf criterion C7-3

1.000000 0.500000 0.125000 1.000000

2.000000 1.000000 0.250000 2.000000

8.000000 4.000000 1.000000 8.000000

1.000000 0.500000 0.125000 1.000000

The PCM HAS acceptable consistency.

Local weights of the alternatives:

1.000000 2.000000 8.000000 1.000000

Global weights of the leaf criterion:

0.146789 0.088073 0.088073 0.044037 0.049541 0.049541 0.066055 0.041284
0.027523 0.027523 0.041284 0.022018 0.088074 0.044037 0.073395 0.029358
0.029358 0.022018 0.022018

Alternatives' global weights:

2.302583 2.485160 2.747810 2.845364

The hierarchy structure:

7 T TOP C1 1 C2 0 C3 1 C4 1 C5 1 C6 1 C7 1

3 C1 T C1-1 0 C1-2 0 C1-3 0

3 C3 T C3-1 0 C3-2 0 C3-3 0

4 C4 T C4-1 0 C4-2 0 C4-3 0 C4-4 0

2 C5 T C5-1 0 C5-2 0

3 C6 T C6-1 0 C6-2 0 C6-3 0

3 C7 T C7-1 0 C7-2 0 C7-3 0

The number of alternatives:4

Criterion: T; Sub-criterion: C1 C2 C3 C4 C5 C6 C7

PCM of Sub-criterion w.r.t. Criterion:

1.000000	1.500000	1.333333	1.600000	2.000000	1.500000	3.000000
0.666667	1.000000	0.888889	1.066667	1.333333	1.000000	2.000000
0.750000	1.125000	1.000000	1.200000	1.500000	1.125000	2.250000
0.625000	0.937500	0.833333	1.000000	1.250000	0.937500	1.875000
0.500000	0.750000	0.666667	0.800000	1.000000	0.750000	1.500000
0.666667	1.000000	0.888889	1.066667	1.333333	1.000000	2.000000
0.333333	0.500000	0.444444	0.533333	0.666667	0.500000	1.000000

The PCM HAS acceptable consistency.

Local weights of the sub-criterion:

0.220183 0.146789 0.165138 0.137615 0.110092 0.146789 0.073394

Criterion: C1; Sub-criterion: C1-1 C1-2 C1-3

PCM of Sub-criterion w.r.t. Criterion:

1.000000 1.000000 2.000000

1.000000 1.000000 2.000000

0.500000 0.500000 1.000000

The PCM HAS acceptable consistency.

Local weights of the sub-criterion:

0.400000 0.400000 0.200000

Criterion: C3; Sub-criterion: C3-1 C3-2 C3-3

PCM of Sub-criterion w.r.t. Criterion:

1.000000 1.000000 0.750000

1.000000 1.000000 0.750000

1.333333 1.333333 1.000000

The PCM HAS acceptable consistency.

Local weights of the sub-criterion:

0.300000 0.300000 0.400000

Criterion: C4; Sub-criterion: C4-1 C4-2 C4-3 C4-4

PCM of Sub-criterion w.r.t. Criterion:

1.000000	1.500000	1.500000	1.000000
0.666667	1.000000	1.000000	0.666667
0.666667	1.000000	1.000000	0.666667
1.000000	1.500000	1.500000	1.000000

The PCM HAS acceptable consistency.

Local weights of the sub-criterion:

0.300000 0.200000 0.200000 0.300000

Criterion: C5; Sub-criterion: C5-1 C5-2

PCM of Sub-criterion w.r.t. Criterion:

1.000000	0.250000
4.000000	1.000000

The PCM HAS acceptable consistency.

Local weights of the sub-criterion:

0.200000 0.800000

Criterion: C6; Sub-criterion: C6-1 C6-2 C6-3

PCM of Sub-criterion w.r.t. Criterion:

1.000000	0.600000	1.500000
1.666667	1.000000	2.500000
0.666667	0.400000	1.000000

The PCM HAS acceptable consistency.

Local weights of the sub-criterion:

0.300000 0.500000 0.200000

Criterion: C7; Sub-criterion: C7-1 C7-2 C7-3

PCM of Sub-criterion w.r.t. Criterion:

1.000000	1.333333	1.333333
0.750000	1.000000	1.000000
0.750000	1.000000	1.000000

The PCM HAS acceptable consistency.

Local weights of the sub-criterion:

0.400000 0.300000 0.300000

Alternatives: A1 A3 A4 A5

Leaf criteria: C2 C1-1 C1-2 C1-3 C3-1 C3-2 C3-3 C4-1 C4-2 C4-3 C4-4 C5-1 C5-2 C6-1 C6-2 C6-3 C7-1 C7-2 C7-3

PCM for Alternatives w.r.t. leaf criterion C2

1.000000	0.333333	0.250000	0.250000
3.000000	1.000000	0.750000	0.750000
4.000000	1.333333	1.000000	1.000000
4.000000	1.333333	1.000000	1.000000

The PCM HAS acceptable consistency.

Local weights of the alternatives:

1.000000 3.000000 3.999997 3.999997

PCM for Alternatives w.r.t. leaf criterion C1-1

1.000000	0.666667	1.250000	1.666667

1.500000	1.000000	1.875000	2.500000
0.800000	0.533333	1.000000	1.333333
0.600000	0.400000	0.750000	1.000000

The PCM HAS acceptable consistency.

Local weights of the alternatives:

1.666666 2.499998 1.333332 1.000000

PCM for Alternatives w.r.t. leaf criterion C1-2

1.000000	3.000000	4.000000	5.000000
0.333333	1.000000	1.333333	1.666667
0.250000	0.750000	1.000000	1.250000
0.200000	0.600000	0.800000	1.000000

The PCM HAS acceptable consistency.

Local weights of the alternatives:

4.999996 1.666665 1.249998 1.000000

PCM for Alternatives w.r.t. leaf criterion C1-3

1.000000	0.500000	2.000000	1.250000
2.000000	1.000000	4.000000	2.500000

0.500000 0.250000 1.000000 0.625000

0.800000 0.400000 1.600000 1.000000

The PCM HAS acceptable consistency.

Local weights of the alternatives:

1.999998 3.999998 1.000000 1.599999

PCM for Alternatives w.r.t. leaf criterion C3-1

1.000000 0.333333 0.250000 0.500000

3.000000 1.000000 0.750000 1.500000

4.000000 1.333333 1.000000 2.000000

2.000000 0.666667 0.500000 1.000000

The PCM HAS acceptable consistency.

Local weights of the alternatives:

1.000000 3.000000 4.000000 2.000000

PCM for Alternatives w.r.t. leaf criterion C3-2

1.000000 0.500000 4.000000 0.250000

2.000000 1.000000 8.000000 0.500000

0.250000 0.125000 1.000000 0.062500

4.000000 2.000000 16.000000 1.000000

The PCM HAS acceptable consistency.

Local weights of the alternatives:

4.000000 8.000005 1.000000 16.000010

PCM for Alternatives w.r.t. leaf criterion C3-3

1.000000	2.500000	0.333333	1.000000
0.400000	1.000000	0.133333	0.400000
3.000000	7.500000	1.000000	3.000000
1.000000	2.500000	0.333333	1.000000

The PCM HAS acceptable consistency.

Local weights of the alternatives:

2.500001 1.000000 7.500001 2.500001

PCM for Alternatives w.r.t. leaf criterion C4-1

1.000000	4.000000	0.500000	5.000000
0.250000	1.000000	0.125000	1.250000
2.000000	8.000000	1.000000	10.000000
0.200000	0.800000	0.100000	1.000000

The PCM HAS acceptable consistency.

Local weights of the alternatives:

4.999997 1.250000 9.999997 1.000000

PCM for Alternatives w.r.t. leaf criterion C4-2

1.000000	0.500000	2.000000	0.200000
2.000000	1.000000	4.000000	0.400000
0.500000	0.250000	1.000000	0.100000
5.000000	2.500000	10.000000	1.000000

The PCM HAS acceptable consistency.

Local weights of the alternatives:

2.000000 4.000003 1.000000 10.000006

PCM for Alternatives w.r.t. leaf criterion C4-3

1.000000	0.200000	0.250000	2.000000
5.000000	1.000000	1.250000	10.000000
4.000000	0.800000	1.000000	8.000000
0.500000	0.100000	0.125000	1.000000

The PCM HAS acceptable consistency.

Local weights of the alternatives:

1.999996 9.999989 7.999989 1.000000

PCM for Alternatives w.r.t. leaf criterion C4-4

1.000000	6.000000	0.500000	4.000000
0.166667	1.000000	0.083333	0.666667
2.000000	12.000000	1.000000	8.000000
0.250000	1.500000	0.125000	1.000000

The PCM HAS acceptable consistency.

Local weights of the alternatives:

5.999994 1.000000 11.999984 1.499997

PCM for Alternatives w.r.t. leaf criterion C5-1

1.000000	3.000000	2.000000	8.000000
0.333333	1.000000	0.666667	2.666667
0.500000	1.500000	1.000000	4.000000
0.125000	0.375000	0.250000	1.000000

The PCM HAS acceptable consistency.

Local weights of the alternatives:

7.999988 2.666664 3.999994 1.000000

PCM for Alternatives w.r.t. leaf criterion C5-2

1.000000	0.500000	4.000000	0.250000
2.000000	1.000000	8.000000	0.500000
0.250000	0.125000	1.000000	0.062500
4.000000	2.000000	16.000000	1.000000

The PCM HAS acceptable consistency.

Local weights of the alternatives:

4.000000 8.000005 1.000000 16.000010

PCM for Alternatives w.r.t. leaf criterion C6-1

1.000000	3.000000	4.000000	1.000000
0.333333	1.000000	1.333333	0.333333
0.250000	0.750000	1.000000	0.250000
1.000000	3.000000	4.000000	1.000000

The PCM HAS acceptable consistency.

Local weights of the alternatives:

4.000004 1.333334 1.000000 4.000004

PCM for Alternatives w.r.t. leaf criterion C6-2

1.000000 4.000000 0.250000 0.500000

0.250000 1.000000 0.062500 0.125000

4.000000 16.000000 1.000000 2.000000

2.000000 8.000000 0.500000 1.000000

The PCM HAS acceptable consistency.

Local weights of the alternatives:

4.000000 1.000000 16.000010 8.000005

PCM for Alternatives w.r.t. leaf criterion C6-3

1.000000 0.500000 0.250000 0.500000

2.000000 1.000000 0.500000 1.000000

4.000000 2.000000 1.000000 2.000000

2.000000 1.000000 0.500000 1.000000

The PCM HAS acceptable consistency.

Local weights of the alternatives:

1.000000 2.000000 4.000000 2.000000

PCM for Alternatives w.r.t. leaf criterion C7-1

1.000000 0.500000 2.000000 0.250000

2.000000 1.000000 4.000000 0.500000

0.500000 0.250000 1.000000 0.125000

4.000000 2.000000 8.000000 1.000000

The PCM HAS acceptable consistency.

Local weights of the alternatives:

2.000003 4.000006 1.000000 8.000008

PCM for Alternatives w.r.t. leaf criterion C7-2

1.000000 0.500000 1.000000 2.000000

2.000000 1.000000 2.000000 4.000000

1.000000 0.500000 1.000000 2.000000

0.500000 0.250000 0.500000 1.000000

The PCM HAS acceptable consistency.

Local weights of the alternatives:

2.000000 4.000000 2.000000 1.000000

PCM for Alternatives w.r.t. leaf criterion C7-3

1.000000 2.000000 0.500000 4.000000

0.500000 1.000000 0.250000 2.000000

2.000000 4.000000 1.000000 8.000000

0.250000 0.500000 0.125000 1.000000

The PCM HAS acceptable consistency.

Local weights of the alternatives:

4.000006 2.000003 8.000008 1.000000

Global weights of the leaf criterion:

0.146789 0.088073 0.088073 0.044037 0.049541 0.049541 0.066055 0.041284
0.027523 0.027523 0.041284 0.022018 0.088074 0.044037 0.073395 0.029358
0.029358 0.022018 0.022018

Alternatives' global weights:

2.513930 2.561084 2.831758 2.932293

The hierarchy structure:

7 T TOP C1 1 C2 0 C3 1 C4 1 C5 1 C6 1 C7 1

3 C1 T C1-1 0 C1-2 0 C1-3 0

3 C3 T C3-1 0 C3-2 0 C3-3 0

4 C4 T C4-1 0 C4-2 0 C4-3 0 C4-4 0

2 C5 T C5-1 0 C5-2 0

3 C6 T C6-1 0 C6-2 0 C6-3 0

3 C7 T C7-1 0 C7-2 0 C7-3 0

The number of alternatives:4

Criterion: T; Sub-criterion: C1 C2 C3 C4 C5 C6 C7

PCM of Sub-criterion w.r.t. Criterion:

1.000000	1.500000	1.333333	1.600000	2.000000	1.500000	3.000000
0.666667	1.000000	0.888889	1.066667	1.333333	1.000000	2.000000
0.750000	1.125000	1.000000	1.200000	1.500000	1.125000	2.250000
0.625000	0.937500	0.833333	1.000000	1.250000	0.937500	1.875000
0.500000	0.750000	0.666667	0.800000	1.000000	0.750000	1.500000
0.666667	1.000000	0.888889	1.066667	1.333333	1.000000	2.000000
0.333333	0.500000	0.444444	0.533333	0.666667	0.500000	1.000000

The PCM HAS acceptable consistency.

Local weights of the sub-criterion:

0.220183 0.146789 0.165138 0.137615 0.110092 0.146789 0.073394

Criterion: C1; Sub-criterion: C1-1 C1-2 C1-3

PCM of Sub-criterion w.r.t. Criterion:

1.000000 1.000000 2.000000

1.000000 1.000000 2.000000

0.500000 0.500000 1.000000

The PCM HAS acceptable consistency.

Local weights of the sub-criterion:

0.400000 0.400000 0.200000

Criterion: C3; Sub-criterion: C3-1 C3-2 C3-3

PCM of Sub-criterion w.r.t. Criterion:

1.000000 1.000000 0.750000

1.000000 1.000000 0.750000

1.333333 1.333333 1.000000

The PCM HAS acceptable consistency.

Local weights of the sub-criterion:

0.300000 0.300000 0.400000

Criterion: C4; Sub-criterion: C4-1 C4-2 C4-3 C4-4

PCM of Sub-criterion w.r.t. Criterion:

1.000000 1.500000 1.500000 1.000000

0.666667 1.000000 1.000000 0.666667

0.666667 1.000000 1.000000 0.666667

1.000000 1.500000 1.500000 1.000000

The PCM HAS acceptable consistency.

Local weights of the sub-criterion:

0.300000 0.200000 0.200000 0.300000

Criterion: C5; Sub-criterion: C5-1 C5-2

PCM of Sub-criterion w.r.t. Criterion:

1.000000 0.250000

4.000000 1.000000

The PCM HAS acceptable consistency.

Local weights of the sub-criterion:

0.200000 0.800000

Criterion: C6; Sub-criterion: C6-1 C6-2 C6-3

PCM of Sub-criterion w.r.t. Criterion:

1.000000	0.600000	1.500000
1.666667	1.000000	2.500000
0.666667	0.400000	1.000000

The PCM HAS acceptable consistency.

Local weights of the sub-criterion:

0.300000 0.500000 0.200000

Criterion: C7; Sub-criterion: C7-1 C7-2 C7-3

PCM of Sub-criterion w.r.t. Criterion:

1.000000	1.333333	1.333333
0.750000	1.000000	1.000000
0.750000	1.000000	1.000000

The PCM HAS acceptable consistency.

Local weights of the sub-criterion:

0.400000   0.300000   0.300000

Alternatives: A1   A2   A4   A5

Leaf criteria: C2   C1-1   C1-2   C1-3   C3-1   C3-2   C3-3   C4-1   C4-2   C4-3   C4-4   C5-1   C5-2   C6-1   C6-2   C6-3   C7-1   C7-2   C7-3

PCM for Alternatives w.r.t. leaf criterion C2

1.000000   0.500000   0.250000   0.250000

2.000000   1.000000   0.500000   0.500000

4.000000   2.000000   1.000000   1.000000

4.000000   2.000000   1.000000   1.000000

The PCM HAS acceptable consistency.

Local weights of the alternatives:

1.000000   2.000000   4.000002   4.000002

PCM for Alternatives w.r.t. leaf criterion C1-1

1.000000   1.000000   1.250000   1.666667

1.000000 1.000000 1.250000 1.666667

0.800000 0.800000 1.000000 1.333333

0.600000 0.600000 0.750000 1.000000

The PCM HAS acceptable consistency.

Local weights of the alternatives:

1.666668 1.666668 1.333335 1.000000

PCM for Alternatives w.r.t. leaf criterion C1-2

1.000000 2.000000 4.000000 5.000000

0.500000 1.000000 2.000000 2.500000

0.250000 0.500000 1.000000 1.250000

0.200000 0.400000 0.800000 1.000000

The PCM HAS acceptable consistency.

Local weights of the alternatives:

5.000004 2.500001 1.250001 1.000000

PCM for Alternatives w.r.t. leaf criterion C1-3

1.000000 1.500000 2.000000 1.250000

0.666667 1.000000 1.333333 0.833333

0.500000 0.750000 1.000000 0.625000

0.800000 1.200000 1.600000 1.000000

The PCM HAS acceptable consistency.

Local weights of the alternatives:

2.000001 1.333334 1.000000 1.600001

PCM for Alternatives w.r.t. leaf criterion C3-1

1.000000 2.000000 0.250000 0.500000

0.500000 1.000000 0.125000 0.250000

4.000000 8.000000 1.000000 2.000000

2.000000 4.000000 0.500000 1.000000

The PCM HAS acceptable consistency.

Local weights of the alternatives:

2.000003 1.000000 8.000008 4.000006

PCM for Alternatives w.r.t. leaf criterion C3-2

1.000000 0.250000 4.000000 0.250000

4.000000 1.000000 16.000000 1.000000

0.250000 0.062500 1.000000 0.062500

4.000000 1.000000 16.000000 1.000000

The PCM HAS acceptable consistency.

Local weights of the alternatives:

3.999994 15.999972 1.000000 15.999972

PCM for Alternatives w.r.t. leaf criterion C3-3

1.000000	5.000000	0.333333	1.000000
0.200000	1.000000	0.066667	0.200000
3.000000	15.000000	1.000000	3.000000
1.000000	5.000000	0.333333	1.000000

The PCM HAS acceptable consistency.

Local weights of the alternatives:

4.999996 1.000000 14.999991 4.999996

PCM for Alternatives w.r.t. leaf criterion C4-1

1.000000	4.000000	0.500000	5.000000
0.250000	1.000000	0.125000	1.250000
2.000000	8.000000	1.000000	10.000000
0.200000	0.800000	0.100000	1.000000

The PCM HAS acceptable consistency.

Local weights of the alternatives:

4.999997 1.250000 9.999997 1.000000

PCM for Alternatives w.r.t. leaf criterion C4-2

1.000000	0.250000	2.000000	0.200000
4.000000	1.000000	8.000000	0.800000
0.500000	0.125000	1.000000	0.100000
5.000000	1.250000	10.000000	1.000000

The PCM HAS acceptable consistency.

Local weights of the alternatives:

1.999996 7.999989 1.000000 9.999989

PCM for Alternatives w.r.t. leaf criterion C4-3

1.000000	0.500000	0.250000	2.000000
2.000000	1.000000	0.500000	4.000000
4.000000	2.000000	1.000000	8.000000
0.500000	0.250000	0.125000	1.000000

The PCM HAS acceptable consistency.

Local weights of the alternatives:

2.000003 4.000006 8.000008 1.000000

PCM for Alternatives w.r.t. leaf criterion C4-4

1.000000	8.000000	0.500000	4.000000
0.125000	1.000000	0.062500	0.500000
2.000000	16.000000	1.000000	8.000000
0.250000	2.000000	0.125000	1.000000

The PCM HAS acceptable consistency.

Local weights of the alternatives:

8.000000 1.000000 16.000000 2.000000

PCM for Alternatives w.r.t. leaf criterion C5-1

1.000000	5.000000	2.000000	8.000000
0.200000	1.000000	0.400000	1.600000
0.500000	2.500000	1.000000	4.000000
0.125000	0.625000	0.250000	1.000000

The PCM HAS acceptable consistency.

Local weights of the alternatives:

8.000005 1.600002 4.000003 1.000000

PCM for Alternatives w.r.t. leaf criterion C5-2

1.000000	0.200000	4.000000	0.250000
5.000000	1.000000	20.000000	1.250000
0.250000	0.050000	1.000000	0.062500
4.000000	0.800000	16.000000	1.000000

The PCM HAS acceptable consistency.

Local weights of the alternatives:

4.000000 20.000012 1.000000 16.000006

PCM for Alternatives w.r.t. leaf criterion C6-1

1.000000	0.500000	4.000000	1.000000
2.000000	1.000000	8.000000	2.000000
0.250000	0.125000	1.000000	0.250000
1.000000	0.500000	4.000000	1.000000

The PCM HAS acceptable consistency.

Local weights of the alternatives:

3.999997 7.999993 1.000000 3.999997

PCM for Alternatives w.r.t. leaf criterion C6-2

1.000000 4.000000 0.250000 0.500000

0.250000 1.000000 0.062500 0.125000

4.000000 16.000000 1.000000 2.000000

2.000000 8.000000 0.500000 1.000000

The PCM HAS acceptable consistency.

Local weights of the alternatives:

4.000000 1.000000 16.000010 8.000005

PCM for Alternatives w.r.t. leaf criterion C6-3

1.000000 0.250000 0.250000 0.500000

4.000000 1.000000 1.000000 2.000000

4.000000 1.000000 1.000000 2.000000

2.000000 0.500000 0.500000 1.000000

The PCM HAS acceptable consistency.

Local weights of the alternatives:

1.000000 4.000002 4.000002 2.000000

PCM for Alternatives w.r.t. leaf criterion C7-1

1.000000 0.200000 2.000000 0.250000

5.000000 1.000000 10.000000 1.250000

0.500000 0.100000 1.000000 0.125000

4.000000 0.800000 8.000000 1.000000

The PCM HAS acceptable consistency.

Local weights of the alternatives:

1.999996 9.999989 1.000000 7.999989

PCM for Alternatives w.r.t. leaf criterion C7-2

1.000000 2.000000 1.000000 2.000000

0.500000 1.000000 0.500000 1.000000

1.000000 2.000000 1.000000 2.000000

0.500000 1.000000 0.500000 1.000000

The PCM HAS acceptable consistency.

Local weights of the alternatives:

2.000000 1.000000 2.000000 1.000000

PCM for Alternatives w.r.t. leaf criterion C7-3

1.000000 4.000000 0.500000 4.000000

0.250000 1.000000 0.125000 1.000000

2.000000 8.000000 1.000000 8.000000

0.250000 1.000000 0.125000 1.000000

The PCM HAS acceptable consistency.

Local weights of the alternatives:

4.000002 1.000000 8.000005 1.000000

Global weights of the leaf criterion:

0.146789 0.088073 0.088073 0.044037 0.049541 0.049541 0.066055 0.041284
0.027523 0.027523 0.041284 0.022018 0.088074 0.044037 0.073395 0.029358
0.029358 0.022018 0.022018

Alternatives' global weights:

2.756190 2.601601 3.104647 3.214868

The hierarchy structure:

7	T	TOP	C1	1	C2	0	C3	1	C4	1	C5	1	C6	1	C7	1
3	C1	T	C1-1	0	C1-2	0	C1-3	0								
3	C3	T	C3-1	0	C3-2	0	C3-3	0								
4	C4	T	C4-1	0	C4-2	0	C4-3	0	C4-4	0						
2	C5	T	C5-1	0	C5-2	0										
3	C6	T	C6-1	0	C6-2	0	C6-3	0								
3	C7	T	C7-1	0	C7-2	0	C7-3	0								

The number of alternatives:4

Criterion: T;   Sub-criterion: C1   C2   C3   C4   C5   C6   C7

PCM of Sub-criterion w.r.t. Criterion:

1.000000	1.500000	1.333333	1.600000	2.000000	1.500000	3.000000
0.666667	1.000000	0.888889	1.066667	1.333333	1.000000	2.000000
0.750000	1.125000	1.000000	1.200000	1.500000	1.125000	2.250000
0.625000	0.937500	0.833333	1.000000	1.250000	0.937500	1.875000
0.500000	0.750000	0.666667	0.800000	1.000000	0.750000	1.500000
0.666667	1.000000	0.888889	1.066667	1.333333	1.000000	2.000000
0.333333	0.500000	0.444444	0.533333	0.666667	0.500000	1.000000

The PCM HAS acceptable consistency.

Local weights of the sub-criterion:

0.220183 0.146789 0.165138 0.137615 0.110092 0.146789 0.073394

Criterion: C1; Sub-criterion: C1-1 C1-2 C1-3

PCM of Sub-criterion w.r.t. Criterion:

1.000000 1.000000 2.000000

1.000000 1.000000 2.000000

0.500000 0.500000 1.000000

The PCM HAS acceptable consistency.

Local weights of the sub-criterion:

0.400000 0.400000 0.200000

Criterion: C3; Sub-criterion: C3-1 C3-2 C3-3

PCM of Sub-criterion w.r.t. Criterion:

1.000000 1.000000 0.750000

1.000000 1.000000 0.750000

1.333333 1.333333 1.000000

The PCM HAS acceptable consistency.

Local weights of the sub-criterion:

0.300000 0.300000 0.400000

Criterion: C4; Sub-criterion: C4-1 C4-2 C4-3 C4-4

PCM of Sub-criterion w.r.t. Criterion:

1.000000	1.500000	1.500000	1.000000
0.666667	1.000000	1.000000	0.666667
0.666667	1.000000	1.000000	0.666667
1.000000	1.500000	1.500000	1.000000

The PCM HAS acceptable consistency.

Local weights of the sub-criterion:

0.300000 0.200000 0.200000 0.300000

Criterion: C5; Sub-criterion: C5-1 C5-2

PCM of Sub-criterion w.r.t. Criterion:

1.000000	0.250000
4.000000	1.000000

The PCM HAS acceptable consistency.

Local weights of the sub-criterion:

0.200000   0.800000

Criterion: C6;   Sub-criterion: C6-1   C6-2   C6-3

PCM of Sub-criterion w.r.t. Criterion:

1.000000   0.600000   1.500000

1.666667   1.000000   2.500000

0.666667   0.400000   1.000000

The PCM HAS acceptable consistency.

Local weights of the sub-criterion:

0.300000   0.500000   0.200000

Criterion: C7;   Sub-criterion: C7-1   C7-2   C7-3

PCM of Sub-criterion w.r.t. Criterion:

1.000000   1.333333   1.333333

0.750000   1.000000   1.000000

0.750000   1.000000   1.000000

The PCM HAS acceptable consistency.

Local weights of the sub-criterion:

0.400000 0.300000 0.300000

Alternatives: A1 A2 A3 A5

Leaf criteria: C2 C1-1 C1-2 C1-3 C3-1 C3-2 C3-3 C4-1 C4-2 C4-3 C4-4 C5-1 C5-2 C6-1 C6-2 C6-3 C7-1 C7-2 C7-3

PCM for Alternatives w.r.t. leaf criterion C2

1.000000 0.500000 0.333333 0.250000

2.000000 1.000000 0.666667 0.500000

3.000000 1.500000 1.000000 0.750000

4.000000 2.000000 1.333333 1.000000

The PCM HAS acceptable consistency.

Local weights of the alternatives:

1.000000 2.000000 3.000000 4.000000

PCM for Alternatives w.r.t. leaf criterion C1-1

1.000000 1.000000 0.666667 1.666667

1.000000 1.000000 0.666667 1.666667

1.500000 1.500000 1.000000 2.500000

0.600000 0.600000 0.400000 1.000000

The PCM HAS acceptable consistency.

Local weights of the alternatives:

1.666667 1.666667 2.500000 1.000000

PCM for Alternatives w.r.t. leaf criterion C1-2

1.000000 2.000000 3.000000 5.000000

0.500000 1.000000 1.500000 2.500000

0.333333 0.666667 1.000000 1.666667

0.200000 0.400000 0.600000 1.000000

The PCM HAS acceptable consistency.

Local weights of the alternatives:

5.000004 2.500003 1.666669 1.000000

PCM for Alternatives w.r.t. leaf criterion C1-3

1.000000 1.500000 0.500000 1.250000

0.666667 1.000000 0.333333 0.833333

2.000000	3.000000	1.000000	2.500000
0.800000	1.200000	0.400000	1.000000

The PCM HAS acceptable consistency.

Local weights of the alternatives:

1.499999 1.000000 2.999998 1.199999

PCM for Alternatives w.r.t. leaf criterion C3-1

1.000000	2.000000	0.333333	0.500000
0.500000	1.000000	0.166667	0.250000
3.000000	6.000000	1.000000	1.500000
2.000000	4.000000	0.666667	1.000000

The PCM HAS acceptable consistency.

Local weights of the alternatives:

2.000000 1.000000 5.999997 3.999997

PCM for Alternatives w.r.t. leaf criterion C3-2

1.000000	0.250000	0.500000	0.250000
4.000000	1.000000	2.000000	1.000000
2.000000	0.500000	1.000000	0.500000
4.000000	1.000000	2.000000	1.000000

The PCM HAS acceptable consistency.

Local weights of the alternatives:

1.000000 4.000002 2.000000 4.000002

PCM for Alternatives w.r.t. leaf criterion C3-3

1.000000	5.000000	2.500000	1.000000
0.200000	1.000000	0.500000	0.200000
0.400000	2.000000	1.000000	0.400000
1.000000	5.000000	2.500000	1.000000

The PCM HAS acceptable consistency.

Local weights of the alternatives:

5.000005 1.000000 2.000003 5.000005

PCM for Alternatives w.r.t. leaf criterion C4-1

1.000000	4.000000	4.000000	5.000000
0.250000	1.000000	1.000000	1.250000
0.250000	1.000000	1.000000	1.250000
0.200000	0.800000	0.800000	1.000000

The PCM HAS acceptable consistency.

Local weights of the alternatives:

4.999997 1.249998 1.249998 1.000000

PCM for Alternatives w.r.t. leaf criterion C4-2

1.000000	0.250000	0.500000	0.200000
4.000000	1.000000	2.000000	0.800000
2.000000	0.500000	1.000000	0.400000
5.000000	1.250000	2.500000	1.000000

The PCM HAS acceptable consistency.

Local weights of the alternatives:

1.000000 4.000003 2.000003 5.000005

PCM for Alternatives w.r.t. leaf criterion C4-3

1.000000	0.500000	0.200000	2.000000
2.000000	1.000000	0.400000	4.000000
5.000000	2.500000	1.000000	10.000000
0.500000	0.250000	0.100000	1.000000

The PCM HAS acceptable consistency.

Local weights of the alternatives:

2.000000 4.000003 10.000006 1.000000

PCM for Alternatives w.r.t. leaf criterion C4-4

1.000000	8.000000	6.000000	4.000000
0.125000	1.000000	0.750000	0.500000
0.166667	1.333333	1.000000	0.666667
0.250000	2.000000	1.500000	1.000000

The PCM HAS acceptable consistency.

Local weights of the alternatives:

8.000006 1.000000 1.333334 2.000002

PCM for Alternatives w.r.t. leaf criterion C5-1

1.000000	5.000000	3.000000	8.000000
0.200000	1.000000	0.600000	1.600000
0.333333	1.666667	1.000000	2.666667
0.125000	0.625000	0.375000	1.000000

The PCM HAS acceptable consistency.

Local weights of the alternatives:

7.999998 1.599999 2.666665 1.000000

PCM for Alternatives w.r.t. leaf criterion C5-2

1.000000	0.200000	0.500000	0.250000
5.000000	1.000000	2.500000	1.250000
2.000000	0.400000	1.000000	0.500000
4.000000	0.800000	2.000000	1.000000

The PCM HAS acceptable consistency.

Local weights of the alternatives:

1.000000   5.000005   2.000003   4.000003

PCM for Alternatives w.r.t. leaf criterion C6-1

1.000000	0.500000	3.000000	1.000000
2.000000	1.000000	6.000000	2.000000
0.333333	0.166667	1.000000	0.333333
1.000000	0.500000	3.000000	1.000000

The PCM HAS acceptable consistency.

Local weights of the alternatives:

3.000000   6.000000   1.000000   3.000000

PCM for Alternatives w.r.t. leaf criterion C6-2

1.000000 4.000000 4.000000 0.500000

0.250000 1.000000 1.000000 0.125000

0.250000 1.000000 1.000000 0.125000

2.000000 8.000000 8.000000 1.000000

The PCM HAS acceptable consistency.

Local weights of the alternatives:

4.000002 1.000000 1.000000 8.000005

PCM for Alternatives w.r.t. leaf criterion C6-3

1.000000 0.250000 0.500000 0.500000

4.000000 1.000000 2.000000 2.000000

2.000000 0.500000 1.000000 1.000000

2.000000 0.500000 1.000000 1.000000

The PCM HAS acceptable consistency.

Local weights of the alternatives:

1.000000 4.000000 2.000000 2.000000

PCM for Alternatives w.r.t. leaf criterion C7-1

1.000000 0.200000 0.500000 0.250000

5.000000 1.000000 2.500000 1.250000

2.000000 0.400000 1.000000 0.500000

4.000000 0.800000 2.000000 1.000000

The PCM HAS acceptable consistency.

Local weights of the alternatives:

1.000000 5.000005 2.000003 4.000003

PCM for Alternatives w.r.t. leaf criterion C7-2

1.000000 2.000000 0.500000 2.000000

0.500000 1.000000 0.250000 1.000000

2.000000 4.000000 1.000000 4.000000

0.500000 1.000000 0.250000 1.000000

The PCM HAS acceptable consistency.

Local weights of the alternatives:

1.999998 1.000000 3.999997 1.000000

PCM for Alternatives w.r.t. leaf criterion C7-3

1.000000 4.000000 2.000000 4.000000

0.250000 1.000000 0.500000 1.000000

0.500000 2.000000 1.000000 2.000000

0.250000 1.000000 0.500000 1.000000

The PCM HAS acceptable consistency.

Local weights of the alternatives:

3.999997 1.000000 1.999998 1.000000

Global weights of the leaf criterion:

0.146789 0.088073 0.088073 0.044037 0.049541 0.049541 0.066055 0.041284
0.027523 0.027523 0.041284 0.022018 0.088074 0.044037 0.073395 0.029358
0.029358 0.022018 0.022018

Alternatives' global weights:

2.134666 2.014938 2.174706 2.489912

The hierarchy structure:

7 T TOP C1 1 C2 0 C3 1 C4 1 C5 1 C6 1 C7 1

3 C1 T C1-1 0 C1-2 0 C1-3 0

3 C3 T C3-1 0 C3-2 0 C3-3 0

4 C4 T C4-1 0 C4-2 0 C4-3 0 C4-4 0

2 C5 T C5-1 0 C5-2 0

3 C6 T C6-1 0 C6-2 0 C6-3 0

3 C7 T C7-1 0 C7-2 0 C7-3 0

The number of alternatives:4

Criterion: T; Sub-criterion: C1 C2 C3 C4 C5 C6 C7

PCM of Sub-criterion w.r.t. Criterion:

1.000000	1.500000	1.333333	1.600000	2.000000	1.500000	3.000000
0.666667	1.000000	0.888889	1.066667	1.333333	1.000000	2.000000
0.750000	1.125000	1.000000	1.200000	1.500000	1.125000	2.250000
0.625000	0.937500	0.833333	1.000000	1.250000	0.937500	1.875000
0.500000	0.750000	0.666667	0.800000	1.000000	0.750000	1.500000
0.666667	1.000000	0.888889	1.066667	1.333333	1.000000	2.000000
0.333333	0.500000	0.444444	0.533333	0.666667	0.500000	1.000000

The PCM HAS acceptable consistency.

Local weights of the sub-criterion:

0.220183 0.146789 0.165138 0.137615 0.110092 0.146789 0.073394

Criterion: C1; Sub-criterion: C1-1 C1-2 C1-3

PCM of Sub-criterion w.r.t. Criterion:

1.000000 1.000000 2.000000

1.000000 1.000000 2.000000

0.500000 0.500000 1.000000

The PCM HAS acceptable consistency.

Local weights of the sub-criterion:

0.400000 0.400000 0.200000

Criterion: C3; Sub-criterion: C3-1 C3-2 C3-3

PCM of Sub-criterion w.r.t. Criterion:

1.000000 1.000000 0.750000

1.000000 1.000000 0.750000

1.333333 1.333333 1.000000

The PCM HAS acceptable consistency.

Local weights of the sub-criterion:

0.300000   0.300000   0.400000

Criterion: C4;   Sub-criterion: C4-1   C4-2   C4-3   C4-4

PCM of Sub-criterion w.r.t. Criterion:

1.000000   1.500000   1.500000   1.000000

0.666667   1.000000   1.000000   0.666667

0.666667   1.000000   1.000000   0.666667

1.000000   1.500000   1.500000   1.000000

The PCM HAS acceptable consistency.

Local weights of the sub-criterion:

0.300000   0.200000   0.200000   0.300000

Criterion: C5;   Sub-criterion: C5-1   C5-2

PCM of Sub-criterion w.r.t. Criterion:

1.000000   0.250000

4.000000   1.000000

The PCM HAS acceptable consistency.

Local weights of the sub-criterion:

0.200000 0.800000

Criterion: C6; Sub-criterion: C6-1 C6-2 C6-3

PCM of Sub-criterion w.r.t. Criterion:

1.000000 0.600000 1.500000

1.666667 1.000000 2.500000

0.666667 0.400000 1.000000

The PCM HAS acceptable consistency.

Local weights of the sub-criterion:

0.300000 0.500000 0.200000

Criterion: C7; Sub-criterion: C7-1 C7-2 C7-3

PCM of Sub-criterion w.r.t. Criterion:

1.000000 1.333333 1.333333

0.750000 1.000000 1.000000

0.750000 1.000000 1.000000

The PCM HAS acceptable consistency.

Local weights of the sub-criterion:

0.400000 0.300000 0.300000

Alternatives: A1 A2 A3 A4

Leaf criteria: C2 C1-1 C1-2 C1-3 C3-1 C3-2 C3-3 C4-1 C4-2 C4-3 C4-4 C5-1 C5-2 C6-1 C6-2 C6-3 C7-1 C7-2 C7-3

PCM for Alternatives w.r.t. leaf criterion C2

1.000000 0.500000 0.333333 0.250000

2.000000 1.000000 0.666667 0.500000

3.000000 1.500000 1.000000 0.750000

4.000000 2.000000 1.333333 1.000000

The PCM HAS acceptable consistency.

Local weights of the alternatives:

1.000000 2.000000 3.000000 4.000000

PCM for Alternatives w.r.t. leaf criterion C1-1

1.000000 1.000000 0.666667 1.250000

1.000000 1.000000 0.666667 1.250000

1.500000 1.500000 1.000000 1.875000

0.800000 0.800000 0.533333 1.000000

The PCM HAS acceptable consistency.

Local weights of the alternatives:

1.250001 1.250001 1.875000 1.000000

PCM for Alternatives w.r.t. leaf criterion C1-2

1.000000 2.000000 3.000000 4.000000

0.500000 1.000000 1.500000 2.000000

0.333333 0.666667 1.000000 1.333333

0.250000 0.500000 0.750000 1.000000

The PCM HAS acceptable consistency.

Local weights of the alternatives:

4.000000 2.000000 1.333333 1.000000

PCM for Alternatives w.r.t. leaf criterion C1-3

1.000000 1.500000 0.500000 2.000000

0.666667 1.000000 0.333333 1.333333

2.000000 3.000000 1.000000 4.000000

0.500000 0.750000 0.250000 1.000000

The PCM HAS acceptable consistency.

Local weights of the alternatives:

2.000000 1.333333 4.000000 1.000000

PCM for Alternatives w.r.t. leaf criterion C3-1

1.000000 2.000000 0.333333 0.250000

0.500000 1.000000 0.166667 0.125000

3.000000 6.000000 1.000000 0.750000

4.000000 8.000000 1.333333 1.000000

The PCM HAS acceptable consistency.

Local weights of the alternatives:

1.999997 1.000000 5.999991 7.999987

PCM for Alternatives w.r.t. leaf criterion C3-2

1.000000 0.250000 0.500000 4.000000

4.000000 1.000000 2.000000 16.000000

2.000000 0.500000 1.000000 8.000000

0.250000 0.062500 0.125000 1.000000

The PCM HAS acceptable consistency.

Local weights of the alternatives:

4.000000 16.000010 8.000005 1.000000

PCM for Alternatives w.r.t. leaf criterion C3-3

1.000000 5.000000 2.500000 0.333333

0.200000 1.000000 0.500000 0.066667

0.400000 2.000000 1.000000 0.133333

3.000000 15.000000 7.500000 1.000000

The PCM HAS acceptable consistency.

Local weights of the alternatives:

5.000000 1.000000 2.000000 15.000003

PCM for Alternatives w.r.t. leaf criterion C4-1

1.000000 4.000000 4.000000 0.500000

0.250000 1.000000 1.000000 0.125000

0.250000 1.000000 1.000000 0.125000

2.000000 8.000000 8.000000 1.000000

The PCM HAS acceptable consistency.

Local weights of the alternatives:

4.000002 1.000000 1.000000 8.000005

PCM for Alternatives w.r.t. leaf criterion C4-2

1.000000	0.250000	0.500000	2.000000
4.000000	1.000000	2.000000	8.000000
2.000000	0.500000	1.000000	4.000000
0.500000	0.125000	0.250000	1.000000

The PCM HAS acceptable consistency.

Local weights of the alternatives:

2.000003 8.000008 4.000006 1.000000

PCM for Alternatives w.r.t. leaf criterion C4-3

1.000000	0.500000	0.200000	0.250000
2.000000	1.000000	0.400000	0.500000
5.000000	2.500000	1.000000	1.250000
4.000000	2.000000	0.800000	1.000000

The PCM HAS acceptable consistency.

Local weights of the alternatives:

1.000000 2.000003 5.000005 4.000003

PCM for Alternatives w.r.t. leaf criterion C4-4

1.000000	8.000000	6.000000	0.500000
0.125000	1.000000	0.750000	0.062500
0.166667	1.333333	1.000000	0.083333
2.000000	16.000000	12.000000	1.000000

The PCM HAS acceptable consistency.

Local weights of the alternatives:

8.000014 1.000000 1.333336 16.000029

PCM for Alternatives w.r.t. leaf criterion C5-1

1.000000	5.000000	3.000000	2.000000
0.200000	1.000000	0.600000	0.400000
0.333333	1.666667	1.000000	0.666667
0.500000	2.500000	1.500000	1.000000

The PCM HAS acceptable consistency.

Local weights of the alternatives:

5.000004 1.000000 1.666669 2.500003

PCM for Alternatives w.r.t. leaf criterion C5-2

1.000000	0.200000	0.500000	4.000000
5.000000	1.000000	2.500000	20.000000
2.000000	0.400000	1.000000	8.000000
0.250000	0.050000	0.125000	1.000000

The PCM HAS acceptable consistency.

Local weights of the alternatives:

3.999995   19.999970   7.999985   1.000000

PCM for Alternatives w.r.t. leaf criterion C6-1

1.000000	0.500000	3.000000	4.000000
2.000000	1.000000	6.000000	8.000000
0.333333	0.166667	1.000000	1.333333
0.250000	0.125000	0.750000	1.000000

The PCM HAS acceptable consistency.

Local weights of the alternatives:

4.000003   8.000003   1.333334   1.000000

PCM for Alternatives w.r.t. leaf criterion C6-2

1.000000 4.000000 4.000000 0.250000

0.250000 1.000000 1.000000 0.062500

0.250000 1.000000 1.000000 0.062500

4.000000 16.000000 16.000000 1.000000

The PCM HAS acceptable consistency.

Local weights of the alternatives:

4.000006 1.000000 1.000000 16.000017

PCM for Alternatives w.r.t. leaf criterion C6-3

1.000000 0.250000 0.500000 0.250000

4.000000 1.000000 2.000000 1.000000

2.000000 0.500000 1.000000 0.500000

4.000000 1.000000 2.000000 1.000000

The PCM HAS acceptable consistency.

Local weights of the alternatives:

1.000000 4.000002 2.000000 4.000002

PCM for Alternatives w.r.t. leaf criterion C7-1

1.000000 0.200000 0.500000 2.000000

5.000000 1.000000 2.500000 10.000000

2.000000 0.400000 1.000000 4.000000

0.500000 0.100000 0.250000 1.000000

The PCM HAS acceptable consistency.

Local weights of the alternatives:

2.000000 10.000006 4.000003 1.000000

PCM for Alternatives w.r.t. leaf criterion C7-2

1.000000 2.000000 0.500000 1.000000

0.500000 1.000000 0.250000 0.500000

2.000000 4.000000 1.000000 2.000000

1.000000 2.000000 0.500000 1.000000

The PCM HAS acceptable consistency.

Local weights of the alternatives:

2.000000 1.000000 4.000000 2.000000

PCM for Alternatives w.r.t. leaf criterion C7-3

1.000000 4.000000 2.000000 0.500000

0.250000 1.000000 0.500000 0.125000

0.500000 2.000000 1.000000 0.250000

2.000000 8.000000 4.000000 1.000000

The PCM HAS acceptable consistency.

Local weights of the alternatives:

4.000006 1.000000 2.000003 8.000008

Global weights of the leaf criterion:

0.146789 0.088073 0.088073 0.044037 0.049541 0.049541 0.066055 0.041284
0.027523 0.027523 0.041284 0.022018 0.088074 0.044037 0.073395 0.029358
0.029358 0.022018 0.022018

Alternatives' global weights:

2.535070 2.392883 2.582620 2.855571
